# A non-symmetrical p97 conformation initiates a multistep recruitment of Ufd1/Npl4

**DOI:** 10.1016/j.isci.2024.110061

**Published:** 2024-05-21

**Authors:** Michal Arie, Donna Matzov, Rotem Karmona, Natalia Szenkier, Ariel Stanhill, Ami Navon

**Affiliations:** 1 Department of Immunology and Regenerative Biology, The Weizmann Institute of Science, Rehovot 7610001, Israel; 2 Department of Chemical and Structural Biology, The Weizmann Institute of Science, Rehovot 7610001, Israel; 3 Department of Natural Sciences, The Open University of Israel, Raanana 4353701, Israel

**Keywords:** Molecular Structure, Structural Biology, Proteomics

## Abstract

*In vitro* experiments and cryo-EM structures of p97 and its cofactor, Ufd1/Npl4 (UN), elucidated substrate processing. Yet, the structural transitions and the related ATPase cycle upon UN binding remain unresolved. We captured two discrete conformations: One in which D1 protomers are ATP bound, while the D2 subunits are in the ADP state, presumably required for substrate engagement with the D2 pore; and a heterologous nucleotide state within the D1 ring in which only two NTDs are in the “up” ATP state that favors UN binding. Further analysis suggests that initially, UN binds p97’s non-symmetrical conformation, this association promotes a structural transition upon which five NTDs shift to an “up” state and are poised to bind ATP. The UBXL domain of Npl4 was captured bound to an NTD in the ADP state, demonstrating a conformation that may provide directionality to incoming substrate and introduce the flexibility needed for substrate processing.

## Highlights

• p97 NTDs assume a heterogeneous configuration that promotes cofactor recruitment
• Post-recruitment, five NTDs transition to an ATP state
• The remaining downed NTD may introduce directionality to substrate processing

## Graphical abstract

**Figure:**
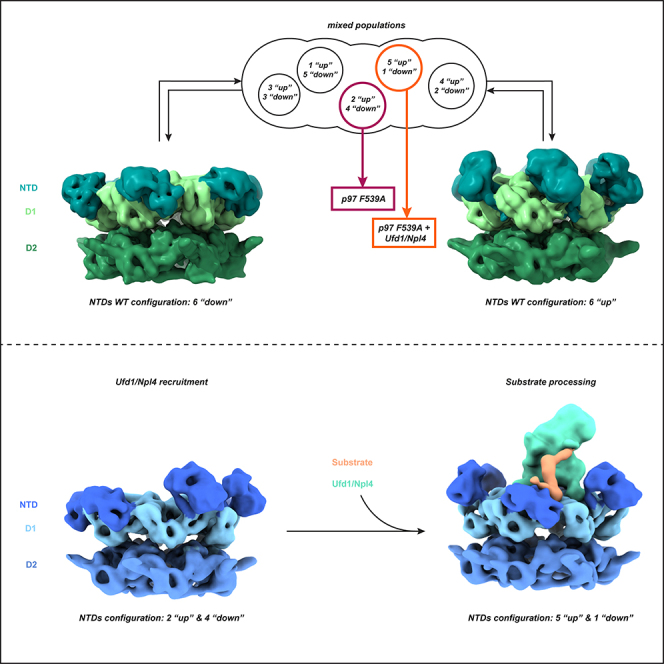

## Introduction

In eukaryotes p97, also known as valosin-containing protein (VCP), or Cdc48 in *S. Cerevisiae*, is one of the most conserved and abundant AAA (ATPases associated with a variety of cellular activities) family member. Many of the processes promoted by p97 are related to the ubiquitin-proteasome system (UPS).^1^^,^^2^^,^^3^ In fact, p97/Cdc48 acts as an unfoldase that couples the extraction of proteins from membranes and complexes to ATP hydrolysis.^4^^,^^5^ The capacity of p97 to unfold and disaggregate is regulated by a large number of cofactors, allowing it to act in multiple cellular pathways.^6^^,^^7^^,^^8^^,^^9^^,^^10^

p97/Cdc48 is classified as type II AAA ATPase, as each protomer contains two consecutive ATPase modules and assembles to a homohexamer with a two stacked rings architecture. Each p97 monomer is traditionally divided into three domains^11^: the amino terminal domain (NTD), the D1 ATPase domain and the D2 ATPase domain. The NTDs are essential for the recruitment of most cofactors, as well as for substrate binding,^12^ whereas the ATPase domains regulate ATP binding and hydrolysis, controlling cofactors binding and substrate processing. Although the D1 and D2 rings share high sequence similarity, the contribution of their ATPase activity is different. *In vitro* assays showed that D2 mutations significantly decrease the ATPase activity of the entire complex, while similar mutations in D1 results in a lower effect.^13^^,^^14^ Therefore, like in many additional type II AAA proteins, the D1 and D2 domains of p97/Cdc48 are not functionally similar. While the D1 ring is responsible for oligomerization and substrate recruitment (Wang et al., 2003), the D2 ring accounts for the major ATPase activity.^13^^,^^15^ Thus, the functional activity of p97 relies on coordination between the D1 and D2 rings.^15^ This is achieved through their nucleotide states, which govern the conformation transitions within the D1 and D2 rings, as well as between the two rings. The current notion is that nucleotide binding to the D1 domain is required for ATP binding to the D2 domain, while nucleotide hydrolysis in D2 is required for the D1 domain to hydrolyze ATP.^14^ However, to date, the precise mechanism supporting the communication between both rings during the nucleotide cycle is still mostly unclear.

Previously reported cryo-EM structures showed two major conformations of the NTDs, which are coupled to specific nucleotide state of the D1 ring: an “up” ATP bound state and a “down” ADP bound conformation.^11^ Symmetry within p97/Cdc48 may be addressed from two related but distinct perspectives (Figure S1): (1) the planarity within the D1 and the D2 rings around the central channel versus a spiral staircase architecture. (2) Uniformity (or symmetry) in regard to the conformation of the individual protomers. In the absence of translocating substrates, most p97/Cdc48 structures determined thus far adopt a 6-fold planar and symmetric conformation of all subunits in each ring (D1 or D2), many times of both rings. In contrast, p97/Cdc48 structures which contain translocating substrates adopt a staircase configuration. The symmetrical arrangements observed in the D1 ring might be related to the fact that traditionally the structures of p97 and its homologues were obtained in the presence of a specific nucleotide or intermediate state analogues, resulting in homogeneous and possibly imposed conformations. Non-symmetrical conformations in which different nucleotides occupy neighboring protomers in the same ring have been captured in other AAA ATPases involved in proteolysis, such as the eukaryotic 19S ATPases.^16^^,^^17^ In contrast, in the absence of translocating substrates, structures of p97/Cdc48 were typically captured in a uniform conformation (all NTDs in the “up” or “down” state), although heterogeneity in respect to the NTDs has been reported in the presence of ADP^18^ (Figure S1). Yet under these conditions p97 NTDs are found dispersed in multiple conformations.

To date about 40 cofactors of p97 have been identified.^9^ The majority of which bind to p97’s NTDs, while a small subset interacts with the C-terminal tail. Additional interacting motifs or cryptic binding domains may also exist, as certain p97 cofactors were found to interact via different sites.^19^^,^^20^^,^^21^ Even though p97 has a myriad of cofactors, it is still unclear how their recruitment is regulated and if that process is ATP dependent.^8^ One of the most studied cofactors is the heterodimer Ufd1-Npl4 (UN) that takes part in the majority of p97/Cdc48 dependent pathways, such as endoplasmic reticulum associated degradation (ERAD) and preemptive quality control (pQC) processes.^2^^,^^22^^,^^23^^,^^24^ In these processes, UN is responsible for binding poly-ubiquitinated substrates, mediating their processing via the association with p97/Cdc48.^25^^,^^26^ At any given time only one UN heterodimer is bound to a hexameric p97 complex.^27^ UN binds p97/Cdc48 through the NTDs in an ATP dependent manner,^28^ that is, in an “up” conformation.^4^^,^^29^^,^^30^ In fact, *in vitro* experiments, and cryo-EM structures show that when UN binds p97/Cdc48, all six NTDs are found in the “up” state.^4^^,^^25^^,^^29^ This raises the question of whether UN binding requires all six NTDs to be in the “up” state or whether it is simply an outcome of the experimental conditions, which included nonhydrolyzable nucleotides or transition state analogues. In contrast to the notion that all NTDs are in a homogeneous conformation, a recent Nuclear Magnetic Resonance (NMR) study demonstrated mixed combinations of “up” and “down” states in the D1 ring (including the NTDs).^31^ This is further supported by structural and biochemical analyses suggesting that the binding of UN heterodimer to p97/Cdc48 may entail engagement with only two adjacent NTDs.^4^^,^^30^

By identifying phenylalanine to alanine p97/Cdc48 mutants that stabilize (but not trap) intermediate conformations, we present biochemical analysis and cryo-EM structures that reveal discrete functional states, captured without the addition of exogenous nucleotide. The fact that the expressing bacteria (*E. coli*) lacks the natural p97 interacting cofactors serves as a useful tool for exploring conformational dependent interactions between p97/Cdc48 and its partners. We present a cryo-EM structure of p97 in which two of the D1 ring NTDs are in the “up” (ATP) state and four are in the “down” (ADP) state. p97 stabilized in this conformation has an elevated affinity toward UN (independent of the addition of exogenous nucleotide). The addition of the cofactor induced a structural transition upon which five of the NTDs are captured in the “up” ATP state. Thus, in line with our Surface Plasmon Resonance (SPR) analysis, we conclude that p97-UN binding entails a two-step reaction mechanism that coordinates UN binding with specific nucleotide state and structural transitions within the D1 ring. We propose that these structural transitions are important for substrate binding, commitment and the necessary hierarchy and coordination between D1 & D2 rings to promote substrate processing. In support of this proposal, a unique conformation, in which the D1 ring protomers are in the ATP state and the D2 ring protomers are all bound to ADP, was also captured and is presumably necessary for the initial substrate entry to the D2 ring pore residues.

## Results

### D1 F266A and D2 F539A mutations capture stable intermediates along p97 nucleotide cycle

**Figure 1:**
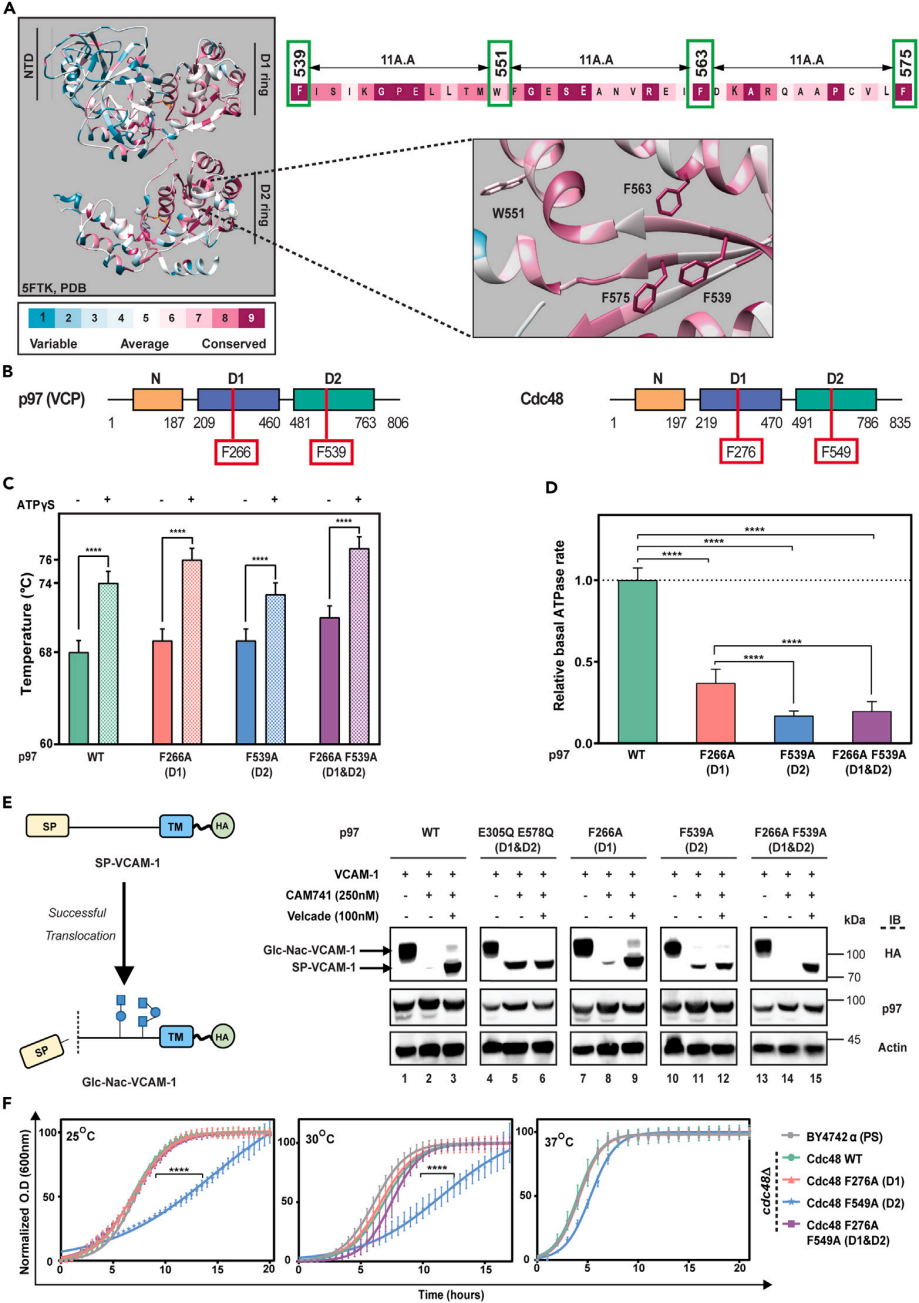
Characterization of p97 phenylalanine mutants (A) The four-phenylalanine signature conservation map overlaid on a human p97 D2 ring protomer, generated using ConSurf.^32^ See also Figure S2. (B) Schematic illustration of the phenylalanine residues studied. (C) p97 phenylalanine mutants are stable and retain the ability to bind ATP. p97 and its mutants were incubated at room temperature in the absence and presence of ATPγS (2mM) and subjected to thermal shift assay (35°C–95°C). The data are represented as mean ± SD. (D) The phenylalanine mutants display lower ATP hydrolysis rate. The indicated p97 species were incubated (0.3 μg each) at 37°C in reaction buffer containing 2 mM ATP; equal samples were collected at the indicated time pointes and the ATPase activity was measured and normalized to wild type p97. The data are represented as mean ± SD. (E) p97 F266A, p97 F539A, and p97 F266A F539A are functional in pQC substrate processing. Left: schematic illustration of the VCAM-1 substrate used before and upon glycosylation and signal sequence removal. Right: western blot analysis of HEK293T cells lysate expressing VCAM-1 and the indicated p97 variants, treated as indicated. Actin served as loading control. (F) Growth rate of yeast harboring Cdc48 D1, or the D1 & D2 double mutant resembles that of wild type, while the D2 mutant gains wild type-like growth rate at elevated temperatures. Growth rate of each strain was normalized to 100%, and the data were fitted using a logistic model. The data are represented as mean ± SD. See also Figure S3.

Previously, we performed an alanine scan on the archaeal proteasome activating nucleotidase (PAN) and identified a mutant (F268A) with low basal ATPase activity in the absence of the 20S proteasome and a normal ATPase activity when proteasomes are present (manuscript in preparation). We have identified PAN F268 as part of a conserved motif, F-11X-Ω-11X-F-11X-F, in which four phenylalanine residues, (aromatic in the second position), are separated by exactly 11 residues (Figure S2). This is illustrated by 150 AAA family members conservation map, overlaid on the p97 3D structure (PDB ID:5FTK), using the ConSurf analysis platform (Figure 1A).^32^ This region is confined between the Walker A and B motifs, which is responsible for ATP binding and hydrolysis, respectively. The current study focuses on the first phenylalanine residue of this signature F266 (D1) and F539 (D2) in human p97, and F276 (D1) and F549 (D2) in yeast Cdc48 (Figure 1B). We generated three mutants of the human p97: F266A (D1), F539A (D2) and the double mutant F266A F539A (D1 & D2). The structural integrity of these mutants was verified by gel filtration, native gel, and negative stain EM, which demonstrated similar properties to the wild type p97 (data not shown). Thermal shift assay (TSA) was used to measure their denaturation temperature (Tm) in the absence or presence of exogenous nucleotide. As shown in Figure 1C, all three mutants had similar Tm values to wild type p97, which was elevated upon ATPγS addition. As shown in Figure 1D, the basal ATPase activities of all three phenylalanine mutants are significantly lower than that of the wild type p97. While F266A (D1) displayed a 2-fold decrease, the D2 F539A and double mutant (F266A F539A) exhibit approximately a 4-fold reduction in their ATPase activity. These finding are in line with the notion that the D2 ring accounts for the major ATPase activity of p97.^13^^,^^14^

To test if these mutants are dominant, we expressed the three p97 mutants alongside the endogenous p97 in mammalian cells and monitored their impact on a p97 dependent processing of a pQC substrate.^22^ As a model substrate, we used the vascular cell adhesion molecule 1 (VCAM-1) that is normally detected in its high-molecular weight form due to glycosylation, which depends on efficient translocation into the ER (Figure 1E, lane 1). Upon CAM741 treatment, a selective ER translocation inhibitor of VCAM-1,^33^ mis-localized VCAM-1 remains in the cytosol and is rerouted by p97 to proteasomal degradation via the pQC pathway (Figure 1E, lane 2). Accordingly, mis-localized VCAM-1 accumulates upon proteasomal inhibition (CAM741+velcade); (Figure 1E, lane 3) or upon p97 inactivation when using a Walker B mutant (p97 QQ: E305Q E578Q; Figure 1E, lane 5).^14^ Using this cellular assay, we evaluate the impact of the indicated p97 variants (F266A, F539A, and the double mutant F266A F539A) on the pQC substrate. The glycosylated isoform is detected in Figure 1E in lanes 1, 4, 7, 10, and 13, as Glc-Nac-VCAM-1, regardless of the transfected p97 variant. In wild type p97 expressing cells, CAM741 treatment eliminated the presence of VCAM-1 (Figure 1E, lane 2), as p97 efficiently reroutes VCAM-1 to proteasomal degradation. As expected, velcade and CAM741 treatment resulted in the accumulation of the non-glycosylated VCAM-1 (SP-VCAM-1, Figure 1E, lane 3). As a dominant negative control, we performed the same experiment in the presence of p97 QQ that is incapable of hydrolyzing ATP, but retains substrate binding capacity, even when co-expressed with wild type p97.^14^ As shown in Figure 1E lane 5, p97 QQ stabilized the non-translocated form of VCAM-1 (SP-VCAM-1) upon CAM741 treatment even in the absence of velcade. Co-expression of the D2 (F539A) mutant resulted in minor attenuation of non-glycosylated VCAM-1 processing (Figure 1E, lane 11). In cells expressing the D1 (F266A) and the D1-D2 (F266A F539A) mutants non-glycosylated VCAM-1 profiles resembled that of the wild type (Figure 1E, lanes 8 and 14). In summary, co-expression of the phenylalanine mutants did not lead to substantial *in vivo* attenuation of p97 dependent process.

To demonstrate that the mutants are functional even in the absence of wild type p97, we reverted to the yeast (*Saccharomyces cerevisiae*) model system (Figure S3). We generated an expression vector (pRS41H) containing CDC48 versions with the complementary Cdc48 D1, D2, and D1&D2 double mutations (Cdc48 F276A, F549A, and F276A F549A). The Cdc48 mutants were placed under the yeast native CDC48 promoter. We then knocked out the endogenous CDC48 gene resulting in haploid strains relying on the ectopic expressed version of CDC48. As evident from Figures 1F and S3, all three derivatives of Cdc48 are viable. While F276A and the double mutant (F276A F549A) grew in all temperatures at a similar rate to the wild type, Cdc48 F549A mutant’s growth was dramatically reduced. However, as seen in Figure 1F, increased temperature restored the growth rate of the D2 (F549A) variant.

Taken together, two conclusions may be inferred from these findings: (1) the phenotype of the mutated Cdc48 does not escalate with increased temperature. In fact, the growth slowdown associated with the D2 mutation diminished, with elevated temperature; (2) the D1 mutation is dominating the D2 mutation in compensating for the growth slowdown. The fact that the growth slowdown is diminished as the temperature increases may imply that the D2 mutation increases the energy barrier between intermediate states, which is reduced upon elevation of the temperature.

### p97 F539A assumes an Ufd1-Npl4 favorable binding conformation independent of exogenous ATP

**Figure 2:**
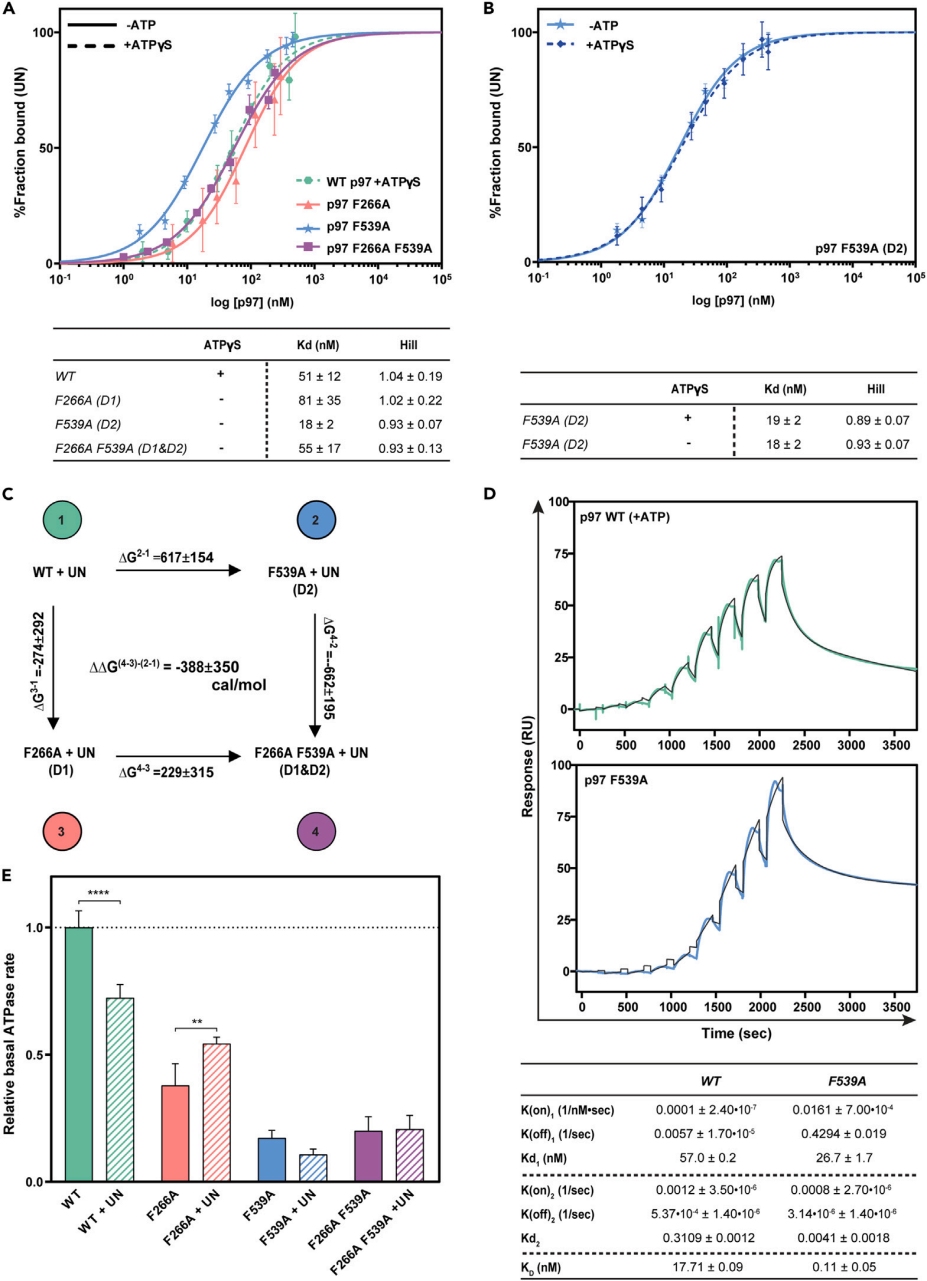
Wild type and phenylalanine p97 mutants affinity and cooperative binding of UN (A) In contrast to wild type p97 the phenylalanine mutants bind UN without the addition of exogenous ATPγS. Top: binding response curve of p97 deduced from UN ELISA binding assay. The fraction of each p97 variant bound to UN was normalized to 100% and the data were fitted using the Hill equation. Except for the wild type p97 that was assayed in the presence of ATPγS (dashed line), all p97 mutants were assayed in the absence of exogenous nucleotide. Bottom: calculated dissociation and Hill coefficients constants. The data are represented as mean ± SD. (B) p97 D2 mutant (F539A) binds UN with the highest affinity which is not further enhanced in the presence of ATPγS. Top: Binding curves for p97 F539A in the presence and absence of ATPγS. Bottom: calculated dissociation and Hill coefficients constants. The data are represented as mean ± SD. (C) The phenylalanine at positions 266 and 539 are allosterically associated in UN binding. Double mutant cycle analysis calculated from the disassociation constants (Kd), obtained in A. (D) The binding mechanism of UN is a two-state model. p97 F539A in the absence of exogenous nucleotide binds UN with higher affinity compared with wild type in the presence of ATP. Wild type p97 (top) and p97 F539A (bottom) binding response curves measured by SPR. Wild type p97 or p97 F539A were immobilized to the chip surface, whereas UN was used as analyte. The calculated rates of association and dissociation constants are presented below the response curves. See also Figure S4. (E) Upon UN binding D2 ring is activated while D1 is inhibited. p97 variants ATPase assay was performed in the presence or absence of UN. The data are represented as mean ± SD.

Traditionally, the structures of p97 and its homologues (in the absence of translocating substrates) were obtained in the presence of a specific nucleotide or an intermediate state analogue, resulting in uniform states and conformations (perhaps imposed) of all the subunits in a given ring (D1 or D2), often in both rings.^11^^,^^25^^,^^34^^,^^35^^,^^36^ Our findings raise the possibility that the phenylalanine mutants may stabilize intermediate states with physiological relevance, circumventing the need to study a uniform structure. One of the most prevalent cofactors responsible for substrate recruitment to p97 is the heterodimer Ufd1-Npl4 (UN), which binds p97 N-terminal domain (NTD) in an ATP dependent manner.^27^ Initially we measured the affinity of wild type p97 toward UN in the presence of ATPγS and the phenylalanine variants in the absence of nucleotide and fitted the results using the Hill equation. As seen in Figure 2A, the affinity of the D1 mutation (F266A) and the double mutant (F266A F539A) in the absence of a nucleotide toward UN was about 55 ± 20nM, similar to that of wild type p97 in the presence of ATPγS. Even in the absence of ATP, p97 F539A (D2) displayed the highest affinity toward UN (3-fold higher), with dissociation constant of about 18 ± 2nM. As wild type p97 binds UN with about 10-fold higher affinity in the presence of ATP,^28^^,^^37^ we tested if the presence of ATPγS would also elevate the affinity of p97 F539A toward UN. As shown in Figure 2B, no elevation in the affinity was observed upon ATPγS addition implying that p97 F539A adopts a conformation with an enhanced affinity toward UN independent of excess ATP. As expected, the Hill coefficient of the wild type p97 and the phenylalanine variants was about 1, in line with binding of a single heterodimer of UN to a p97 hexamer.^27^ To examine if residues F266 and F539 are mechanistically coordinated upon UN binding, a double mutant cycle was calculated^38^^,^^39^ (Figure 2C) using the dissociation constants described in Figure 2A. A calculated delta ΔG value different than zero would indicate a correlation (positive or negative) between two residues. The deduced Gibbs free energy of p97 F266A F539A upon UN binding ($\Delta {\Delta G}_{2-1}^{4-3}=-388\pm 350\frac{cal}{mol}$), represents the coupling energy between the two phenylalanine side chains and is in line with coordination during the structural transition associated with UN binding, indicative of cooperativity between the D1 and the D2 rings. To complement these findings SPR assays were used and enabled us to deduce kinetic parameters of immobilized wild type p97 or p97 F539A toward UN. While binding measurements of p97 wild type were performed in the presence of ATP, p97 F539A was tested in the absence of exogenous nucleotide, as was measured in the ELISA assay (Figure 2A). As shown in Figure 2D, the response curves could only be fitted to a two-step reaction model. This finding supports the notion that UN binding entails two discrete events: the initial UN association with p97, which ensues further conformation transition within p97. The rate limiting step of the initial binding is represented by the first association (K(on)_1_) and dissociation (K(off)_1_) constants (Figure S4), whereas the latter conformation transition is represented by the second association (K(on)_2_) and dissociation (K(off)_2_) constants (Figure S4). In line with the ELISA analysis (Figure 2A), p97 F539A has higher affinity toward UN (Kd_1_ = 26.7 ± 1.7nM) compared with wild type p97 (Kd1 = 57.0 ± 0.2nM). Notably, the latter conformation transition appears to be more dynamic in wild type p97 (Kd_2_ = 0.3109 ± 0.0012nM), presumably due to the elevated nucleotide turnover, in contrast to the more restrained p97 F539A (Kd_2_ = 0.0041 ± 0.0018nM) (Figure S4).

Next, we examined the ATPase activity of wild type p97 and each of the phenylalanine variants (F266A, F539A, and F266A F539A) in the presence and absence of UN (Figure 2E). In the absence of UN, all p97 phenylalanine derivatives had a significantly lower basal ATPase activity relative to the wild type (Figure 1D). Addition of UN to wild type p97 sample resulted in a significant reduction in its ATPase activity, as was previously reported.^12^ p97 F266A had an intermediate ATPase activity, attributed to its D2 active ring, that was further elevated upon UN binding (Figure 2E), suggesting that UN activates the ATPase activity of the D2 ring.^12^ F539A and the double mutant had the lowest ATPase in the absence or presence of UN, presumably as these mutants harbor a mutation in the D2 ring, which is considered to contribute most of the ATPase activity of p97 (Figure 1D). Taking together, UN binding reduces D1 ATP hydrolysis, preventing its premature release, while activating the ATPase activity of the D2 ring to promote substrate processing.^12^

### The F266A mutation traps the D1 ring in the ATP state, while the D2 ring is in the ADP conformation

**Figure 3:**
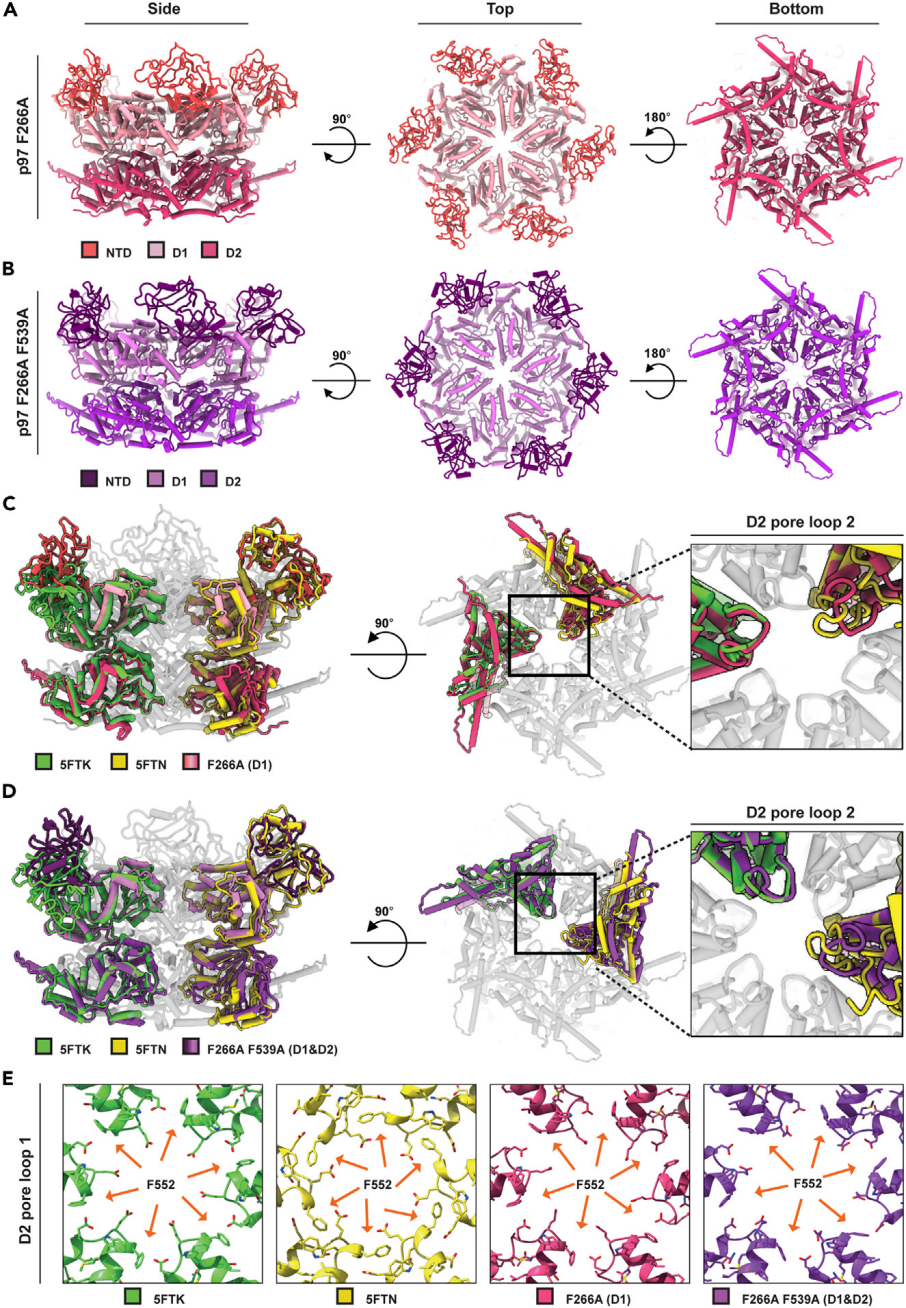
Cryo-EM structures of p97 F266A and F266A-F539A mutants Coordinates model shown from side (left), top (middle) and bottom (right) views of (A) p97 F266A at 2.7 Å resolution and (B) F266A F539A double mutant at 3.4 Å resolution. See also Figures S5‒S8. (C) and (D) Comparison of the NTDs of p97 F266A and p97 F266A F539A, respectively, to the NTDs of wild type p97 3D structure in the presence of ATPγS (PDB ID:5FTN) or ADP (PDB ID:5FTK). As shown in C and D middle and right panels, the D2 rings aligned with the ADP conformation. (E) The D2 ring pore loop 1 (P545-N558) of p97 F266A and p97 F266A F539A double mutant also resemble the ADP state, as evident from the pore dimension and the interactions between neighboring subunits constituting the pore.

To structurally study the effects of the D1/D2 mutations on p97 we used single particle cryo-EM on both the single (F266A, D1) and the double (F266A F539A, D1-D2) mutated proteins and obtained 3D reconstitutions at 2.7 Å and 3.4 Å, respectively (Figures 3A, 3B, and S5–S8). Overall, in both structures the D1 ring highly resembles the conformation of wild type p97 bound to ATPγS. Namely, all six NTD’s are pointing upwards (PDB ID: 5FTN).^11^ However, in both mutants, the D2 ring resembles the ADP-bound state of the wild type p97 (PDB ID:5FTK),^11^ as evident from the dilated D2 ring pore (Figures 3C‒3E). Given that the proteins were purified in the absence of exogenous nucleotides and both mutants present poor ATP hydrolyzing capacity, while retaining the ability to bind ATP (Figures 1B and D), we postulate that the obtained structures represent energetically stable conformations in the overexpressing bacteria. The strong structural similarity of these mutants is in line with the similar effect they had on the growth rates, pQC capacity and UN binding affinity (Figures 1 and 2). The reconstructions were obtained applying both C1 and C6 symmetries during data processing, and thus are not due to imposed symmetry operations (Figures S5 and S7).

### p97 F539A (D2 ring) stabilizes a conformation poised for Ufd1-Npl4 binding

**Figure 4:**
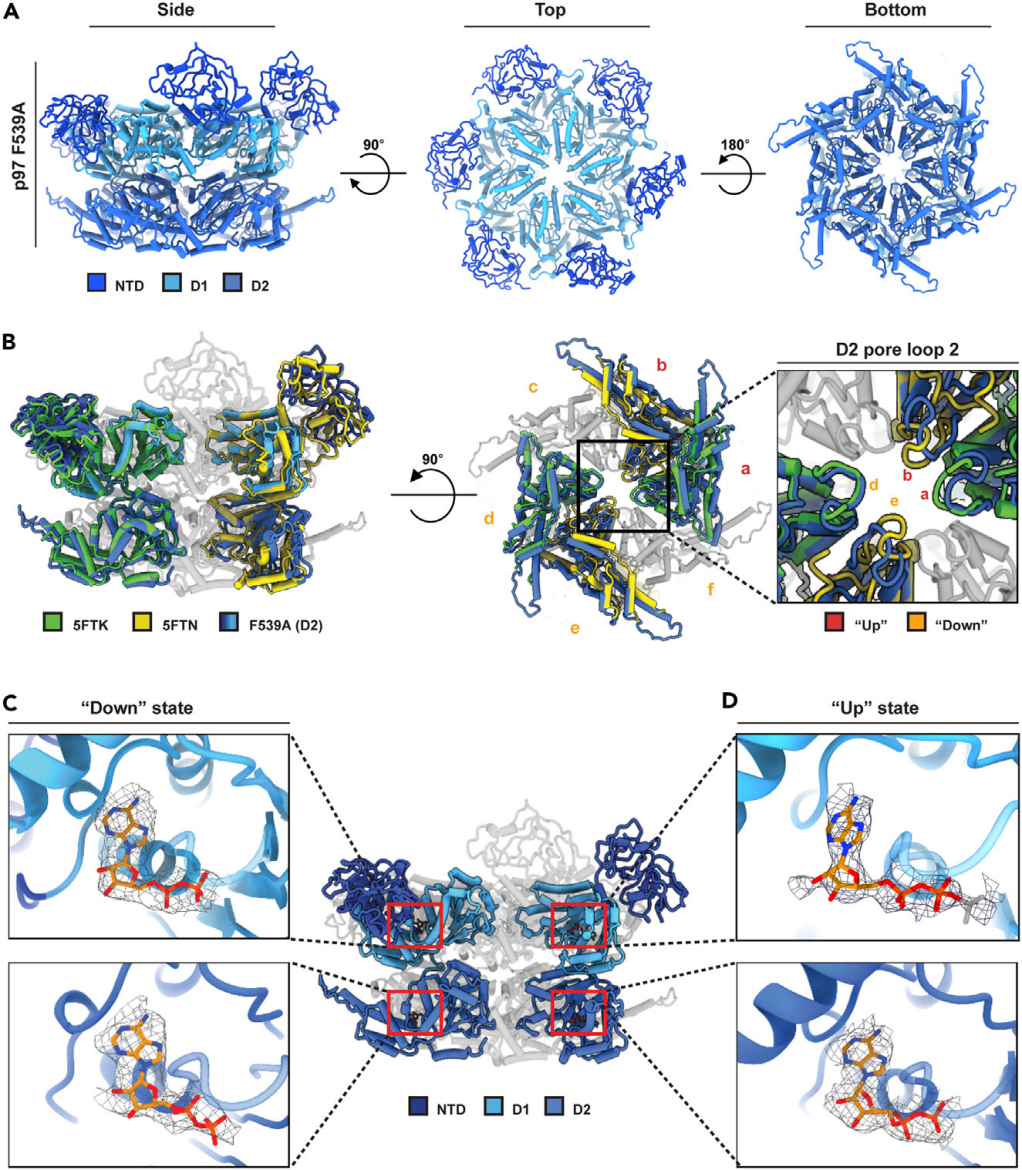
The structure of p97 F539A reveals an asymmetrical conformation within the D1 ring (A) Coordinates model of p97 F539A shown from side (left), top (middle) and bottom (right) views at 3.6 Å resolution. See also Figures S9 and S11. (B) Two of the D1 ring protomers are in the ATP state and four are ADP bound, while the D2 protomers are all in the ADP bound conformation. As shown in the left panel two of the D1 ring NTDs align with the ATPγS bound conformation (PDB ID:5FTN) and four of the NTDs align with the ADP structure (PDB ID:5FTK). In the middle and right panels, the D2 ring architecture aligns with the ADP conformation in agreement with the nucleotides assigned to the D2 protomers. (C and D) p97 F539A coordinates model focusing on the nucleotide pockets in D1 and D2 rings reveals that two of the D1 ring protomers align with the ATP state (D) and the additional four with the ADP bound state (C). All D2 protomers are bound to ADP (C and D).

The binding affinity assays demonstrated that p97 F539A binds the UN cofactor with elevated affinity even in the absence of ATP (Figures 2A and 2D). In fact, addition of nucleotides did not further increase the binding affinity (Figure 2B), suggesting that this mutation transitions p97 to a UN ultimate binding conformation. Unanticipated cryo-EM analysis of this mutant to 3.6 Å (Figure 4A) revealed a non-symmetrical conformation where two of the D1 ring protomers are occupying the wild type p97 ATP bound conformation (“up”; PDB ID:5FTN), while the remaining four correspond to the ADP bound state (“down”; PDB ID:5FTK) (Figures 4B and 4C). In contrast to D1, the D2 ring appeared symmetrical across all protomers and resembled the ADP-bound conformation, as illustrated by D2 ring pore loop 2 alignment (Figures 4B–4D). The replacement of the phenylalanine by an alanine in the D2 ring (F539A) did not inflict major structural perturbation to that ring (Figure S11). As seen in Figure 4D, for the two protomers with the NTDs in the “up” state, density that could accommodate an ATP molecule was observed. As the signal from the third phosphate is low, we could not confidently differentiate between an ATP and an ADP molecule based on density alone. However, as in previous studies the same conformation was observed for the ATPγS bound state (PDB ID: 5FTN), we postulate that the density corresponds to ATP, and thus agrees with the ATP bound state. Combined with our biochemical data indicating an enhanced affinity of UN to the F539A mutant in an ATP independent manner (Figure 2A), we propose that the non-symmetrical conformation of the mutant promotes UN binding through stabilization of a binding-favorable conformation^4^^,^^30^ (Figures 4A, S9, and S11). It is noteworthy that although all D2 protomers carry the same mutation, it populates a non-symmetrical conformation within the D1 ring, supporting the notion that the mutation merely subtilizes a functional intermediate (that has been detected before^18^) rather than an artificial conformation.

Taken together, these findings demonstrate, for the first time, a functional conformation of p97 in which protomers within the D1 ring are in a different nucleotide state even in the absence of an exogenous binder. Notably, to exclude the possibility that this conformation is assumed post purification and does not represent a stable conformation within the expressing bacteria, we determine the structure of p97 F539A in the presence of ATP and obtained similar structure to that presented in Figure 4A (Figures S10 and S11).

Three conclusions may be deduced from these findings: (1) The D1 (F266A) mutation is dominant over the D2 (F539) mutation and thus imposes on the double mutant the same conformation as was determined for the F266A mutant. This conclusion is in line with the very minor phenotype both the F266A mutation and the double mutation (F266A F539A) had on the functionality of these mutants in cells, as well as the lack of significant effect when substituting the wild type CDC48 in yeast (Figures 1E and 1F). (2) In accordance with the all-“up” conformation of the NTDs, the affinity toward UN of these mutants (F266A and F266A F539A) in the absence of ATP resembled that of the wild type p97 in the presence of ATPγS. Presumably, as the NTDs are already in the preferred UN binding conformation (Figure 2A). (3) UN binding by p97 favors a conformation in which the symmetry of the D1 ring is broken with a minimal requirement of at least two of the NTDs in the ATP conformation.^4^^,^^30^ In agreement, the affinity of p97 F539A toward UN is three times higher than that of the wild type p97 or the other mutants, even in the presence of ATPγS (Figures 2B and 2D).

### Binding of Ufd1-Npl4 to p97 F539A favors NTD’s transition to the “up” state

**Figure 5:**
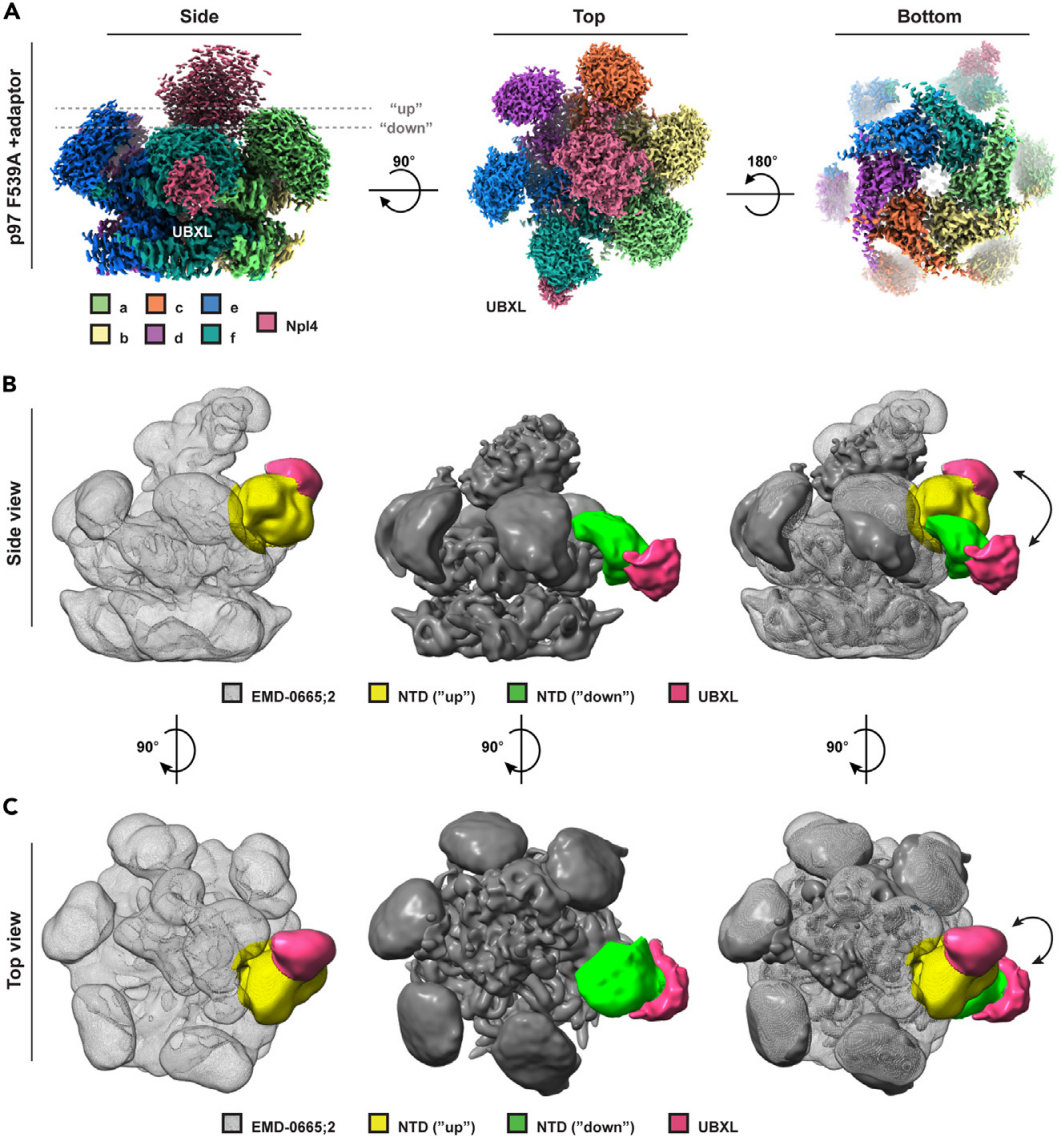
UN binding by p97 F539A favors transition of five NTDs to the "up" state even in the absence of exogenous nucleotide Upon binding of p97 F539A to the cofactor, five of its NTDs transition to the “up” state. (A) cryo-EM map of p97 F539A-adaptor complex at different views (3.0 Å resolution). See also Figures S12 and S13. p97’s six protomers (a-f) are presented in rainbow colors and Npl4 is shown in pink. The NTDs (unsharpened map) are shown at σ = 0.0029, D1 and D2 domains (sharpened map) are presented at σ = 0.0289 and Npl4 (unsharpened map) is presented at σ = 0.0289. Dashed lines (gray) indicate the “up” or “down” state. (B) Side and (C) Top views of (left) Cdc48-Ufd1/Npl4-substrate map (EMD-0665; additional map 2) and (middle) p97 F539A-adaptor map (from panel A), shown following Gaussian filter. The merged maps are presented on the right. The UBXL domains are shown in pink, while the NTDs marked in yellow are in the “up” state and in green are in the “down” state.

As mentioned previous, most structures of p97/Cdc48 and its cofactors were obtained in the presence of nonhydrolyzable nucleotides, transition state analogs or by using p97 mutans (such as walker B mutans). Presumably, these conditions favor a uniform symmetric, all-“up” ATP conformation of the NTDs. Albeit, in the obtained complexes Npl4 was detected associated with multiple NTD’s.^34^ We thus set to determine the structure of p97 F539A and the adaptor complex. Briefly, the adaptor was absorbed to a Ni-NTA column by virtue of the His-tag and the unbound materials were thoroughly washed. An excess bacterial lysate containing overexpressed untagged p97 F539A was passed through the column and was washed thoroughly. The complexes were eluted by an imidazole gradient and rapidly subjected to size exclusion chromatography. The purified complexes were analyzed by single particle cryo-EM analysis and the 3D reconstruction of the complex was solved to 3.0 Å (Figures 5A, S12, and S13).

Surprisingly, five NTDs adopted the “up” ATP conformation and one NTD appeared in the ADP “down” state (Figure 5A). Two additional densities were detected on the p97 D1 ring: one, located in the center of the D1 ring, depicted in pink (Figure 5A), aligns with the previously assigned C terminal domain of Npl4 (PDB ID:6JWJ). Notably, while the overall resolution of the structure was about 3.0 Å, the C terminal domain of Npl4 was observed at lower local resolution, and when setting a higher signal to noise ratio. We also observed a second density, associated with a downed NTD (depicted in pink), which presumably corresponds to the previously assigned UBXL domain of Npl4 (PDB ID:2PJH; Figures 5A and S13).^25^^,^^34^ These findings suggest that cofactor binding by itself promotes (and stabilizes) a conformation in which five of the NTDs are in the “up” ATP state, in line with the suppressive effect UN exerts on p97 ATPase activity in the absence of substrates (Figure 2E).^12^ Notably, previous low resolution cryo-EM analysis already suggested a possible conformation in which Npl4 UBXL domain is associated with an NTD in the ADP, “down” state.^40^ The density map of the adaptor in the complex appeared dynamic and did not permit the visualization of secondary structures. To corroborate our finding, we preformed mass spectrometry analysis of p97 crosslinked to its cofactor as performed in prior studies.^29^^,^^40^ As seen in supplementary Figures S13F and S13G, we detected cross linking pairs corroborating the positioning of Npl4 C-terminal globular domain above the center of the D1 ring. We also detected crosslinks that can only be accounted for given the Npl4 UBXL domain being bound to an NTD in the “down” state.

Three conclusions may be inferred from these findings: the association between p97 and UN follows a two-step reaction mechanism, in line with the SPR analysis (Figure 2D). Second, upon association UN promotes and stabilizes a conformation in which five of the NTDs transition into the ATP conformation and is poised to bind ATP. Third, one NTD, which is bound to Npl4 UBXL domain may remain in the “down” ADP state, perhaps allowing for directionality in substrate engagement, in agreement with the previously proposed substrate processing mechanism.^41^

### p97 F539A protomers are able to transition between nucleotide states

**Figure 6:**
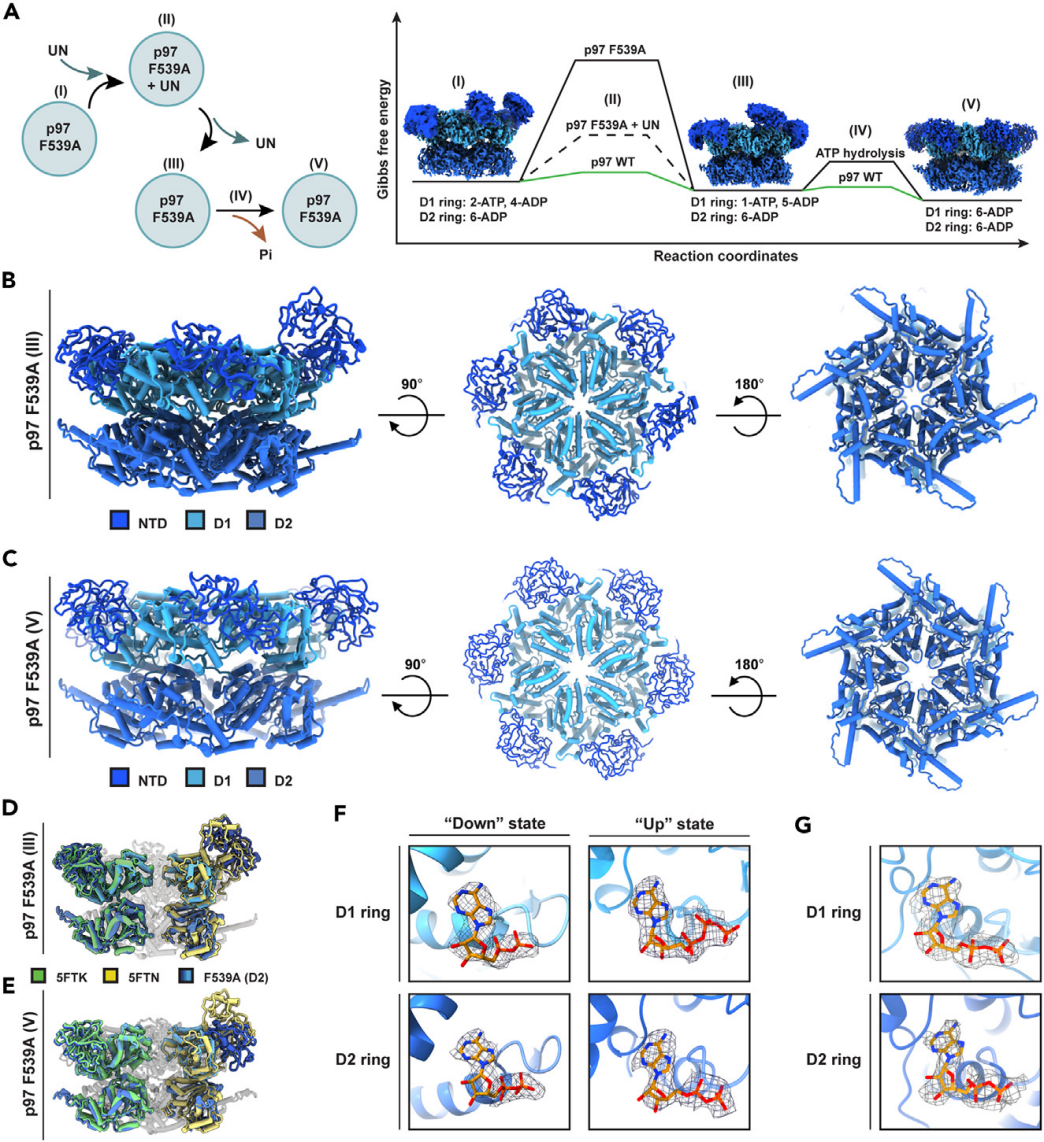
p97 F539A completes the nucleotide cycle upon UN binding and release (A) Left: an illustration of p97 F539A-UN binding and dissociation cycle. State I: Noncomplexed p97 F539A (D1: 2-ATP, 4-ADP). State II: Upon incubation of p97 F539A with UN @37°C. State III: p97 F539A following UN release (D1:1-ATP, 5-ADP). State IV: hydrolysis of the remaining ATP bound molecule. State V: p97 F539A followed ATP hydrolysis (D1: 6-ADP). Presumably, in the absence of ATP (*in vitro*), the nucleotide cycle of p97 F539A terminates at an ADP state (V), while in the presence of ATP an additional subunit would exchange for ATP to facilitate association with incoming UN-substrate complex. Right: Gibbs free energy diagram demonstrating the stabilizing effects of p97 F539A prior to UN binding (D1:2-ATP, 4-ADP) and following UN release (D1:1-ATP, 5-ADP) (black line) compared with WT p97 (green line). (B) Coordinates model of non-complexed p97 F539A (state III) obtained upon dissociation from UN, shown from side, top and bottom views at 3.3 Å. See also Figures S14 and S16. (C) Coordinates model of the ADP bound state p97 F539A (state V) obtained following UN incubation and ATP hydrolysis, shown from side, top and bottom views at 3.4 Å. See also Figures S15 and S16. (D) One of the D1 ring protomers are in the ATP state (PDB ID:5FTN, yellow) and five are ADP bound (PDB ID:5FTK, green), while the D2 protomers are all in the ADP bound conformation. (E) All six p97 protomers align with the ADP bound state (PDB ID:5FTK, green) assumed upon UN release and ATP hydrolysis. (F) Nucleotide pockets of p97 F539A state III: densities that accommodate ADP are detected in all D1 and D2 “down” protomers nucleotide binding pockets, in contrast to the “up” protomer where density that may accommodate ATP molecule was observed. D2 ring of this protomer is occupied by ADP. (G) Nucleotide pockets of p97 F539A state V: A representative “down” monomer encompassing both the D1 and D2 rings is shown.

To demonstrate that p97 F539A is not a trap mutant, and all its protomers are able to complete the nucleotide binding and hydrolysis cycle, we set to capture a conformation where all its NTDs are in the “down” ADP state. To this end p97 F539A and UN were mixed without the addition of exogenous nucleotides, and incubated for 5 or 30 min at 37°C. The samples were then subjected to size exclusion chromatography and the fractions corresponding to the highest molecular weight were used for single particle cryo-EM analysis. The shorter incubation yielded two populations of p97 F539A (Figure S14): a heterogeneous conformation within D1 ring, consisting of a single NTD in the “up” (ATP) state and second, a homogeneous conformation in which all the NTDs are in the “down” (ADP) state. Both 3D reconstructions were solved to high resolutions of 3.3 Å and 3.0 Å, respectively (Figures 6B–6D, 6F, S14, and S16). The longer incubation resulted in a single population in which all NTDs are in the “down” (ADP) state (Figures S15 and S16). This 3D reconstruction was solved to 3.4 Å resolution (Figures 6C–6E, 6G, S15, and S16). Notably, although no symmetry was imposed during processing of this structure, ADPs were detected in all protomers in both D1 and D2 rings (Figure 6G). These findings support a step-by-step (sequential hydrolysis) rather than synchronous mechanism in line with the proposed hand-over-hand mechanism.^41^

Taken together we propose that initially, p97 F539A adopts a two NTDs in the ATP (“up”) state (in D1 ring) (Figure 6A, state I), a conformation that is favorable for UN binding. The inability of this mutant to transition from state (I) to state (III) spontaneously might be attributed to the increased Gibbs free energy between the two states (Figure 6A), in line with its low basal ATPase activity (Figure 1D). We propose that UN binding reduces the energy barrier between the two states, enabling p97 F539A to progress through its catalytic cycle (state V).

## Discussion

Multiple crystal structures of p97 domains, as well as full-length structures were previously determined and were also complemented by high-resolution single-particle cryo-EM structures.^11^^,^^29^^,^^34^^,^^36^^,^^40^^,^^41^^,^^42^^,^^43^^,^^44^ Yet, the structural transitions that accompanied UN recruitment were not resolved. To detect and characterize structurally and mechanistically functional conformation related to the initial cofactor recruiting steps, we identified p97 mutants that do not render p97 inactive but rather stabilize intermediate states (Figure 1) and allow for detection of transient conformations in which the symmetry within the D1 ring is broken (Figure 4). Supporting this notion, we captured p97 F539A in multiple conformations along its catalytic path: by itself it assumes a conformation in which two of the D1 ring protomers are bound to ATP, while the remaining four are bound to ADP (Figure 4). In fact, we failed to detect a defined population of this mutant in an alternative conformation (Figure S9). We propose that the F539A mutation merely stabilizes one of the nucleotide states that are constantly sampled by the cellular p97 population. Presumably this fraction of p97 is poised to associate with UN. In support of this, the dynamic nature of the NTDs was previously reported.^18^ We propose that once p97 F539A is purified from the bacterial lysate, in the absence of excess cellular ATP, it decays to the stabilized conformation in which only two of the NTDs are in the “up” ATP state. In fact, incubating purified p97 F539A in the presence of cellular levels of ATP (2mM), prior to cryo-EM single particle analysis, revealed only two distinct populations. A population in which all D1 NTDs are in the “down” ADP state and a population in which only two of the NTDs are in the “up” ATP state (Figures S9 and S11). These results confirm that the conformation stabilized by p97 F539A in the absence of nucleotide represents the most energetically stable conformation *in vivo* even when ATP is present at high concentrations. As evident from our affinity and gel-filtration analyses, this mutant can directly engage with UN to form a stable complex, which dissociates upon ATP hydrolysis (Figure 6). Notably, this association is not altered by the presence of nucleotides (Figures 2A and 2B).

The presence of UN has been shown to exert repressive effect on the ATPase activity of p97. This is in line with the fact that cofactor binding shifts the NTD conformation to a five “up” (ATP) binding prone conformation (Figure 6A). Furthermore, as evident from the SPR analysis, the complex between UN and p97 F539A is long lived, as deduced from the low K_off_ (Figure 2D). Taken together, we propose that while the association of UN with the D1 ring NTDs promotes charging of five protomers with ATP, it prevents nucleotide hydrolysis (Figure 2E). Only once the substrate processing by the D2 ring is completed, can the D1 ring be signaled to hydrolyze the nucleotides and detach from the UN cofactor. Such a mechanism is advantageous, as it introduces hierarchy and coordination between the D1 and the D2 rings. While the D1 ring regulates substrate recruitment and positioning, the D2 ring translocates and unfolds the incoming substrate. The multiple nucleotides bound and hydrolyzed in the transitions of the D1 ring upon UN binding and release provide a tight control and perhaps increases the available mechanical force due to the multiple coordinated nucleotide hydrolyses. The sixth NTD detected in the current study in the ADP "down" state, bound to Npl4 UBXL domain, may account for the flexibility and dynamics needed during substrate translocation (Figures 5B and 5C).

Npl4 is composed of two globular domains connected by an unstructured region. While the C-terminal main globular domain was detected in multiple Cdc48/p97 structures above the entry channel of the D1 ring, Npl4 UBXL domain was observed bound to one of the NTD’s in the “up” state (see cryo-EM maps EMD-0665 for CDC48 and EMD-20730 for p97-A232E).^25^^,^^36^ Here, we detected Npl4 UBXL domain bound to an NTD in the ADP “down” conformation, a finding that is further supported by cross-linking analysis (Figures S13F and S13G). Super positioning of our current structure onto the previously resolved Cdc48/p97 UN complexes (Figure 5) reveals that Npl4 UBXL domain was detected bound to the same NTD, however, it was captured in the "down" conformation (Figures 5B and 5C). Alignment of the two maps illustrates the possible motion of Npl4 UBXL domain upon conformational transition of the cognate NTD from the “up” (ATP) state to the “down” (ADP) state and vice versa (Figures 5B and 5C, right). Taken together we propose that initially UN binds p97’s non-symmetrical conformation (two NTDs in an “up” state). This association promotes a structural transition upon which five NTDs shift to an “up” state and are poised to bind ATP. The UBXL domain of Npl4 selectively binds p97’s downed NTD, a conformation that may introduce directionality to incoming substrate and perhaps link its translocation to the nucleotide state. ATP binding in the downed NTD may occur, following the initiation of unfolding and staircase arrangement, as was previously reported.^25^^,^^34^^,^^41^^,^^43^^,^^45^ Notably, heterogeneous NTD conformations associated with specific adaptors have been reported in other type-II AAA ATPases,^46^ thus further evaluations are required to establish if these conformations may serve as preferential binding of specific adaptors.

Multiple biochemical and structural studies have shown that UN binding and substrate processing require the D1 ring to be in the ATP state, as well as at least some of the protomers (if not all) within the D2 ring.^25^^,^^34^ The current study illustrates an inter-ring communication and regulation whereby mutation in the D2 ring (F539A) promotes stabilization of a conformation in which two of the D1 ring NTDs are in the ATP state, while the remaining four are in the ADP state (Figure 4). Furthermore, we propose a functional significance to a previously detected and uncharacterized state, in which the D1 ring is in the ATP state while the D2 is in the ADP state (Figure 3).^47^

Additional insights inferred from our findings address the transition of the D2 ring between the ATPγS (PDB ID:5FTN) and the ADP (PDB ID:5FTK) states and the size of its central pore. The D2 orifice is composed of a two iris structures (Figure S17): the first is at the entry (pore loop 1: residues P545-N558) and the second is at the exit (pore loop 2: D580-I604). As seen in Figure S17, F552 and W551, two important residues that define the rim of the first iris, change their conformation upon transition from ADP to ATP. F552 of a given protomer interacts with W551 of the adjacent protomer (π-π interaction). Incidentally, these residues were defined as key elements in substrate engagement and translocation.^25^^,^^34^^,^^48^ Additionally, in the ATP state, F552 rotates to the plane of the rim and becomes sandwiched between E554 and E556 of the adjacent protomer, perhaps forming anion-π interactions.^49^ This network of contacts restricts F552 mobility, thus stabilizing protomer interactions (termed “Tight” state in Figure S17B). In contrast, in the ADP state, F552 side chain is no longer “trapped” in this interaction network and contacts between adjacent protomers in this region loosen, (termed “Relax” state in Figure S17B). As shown in Figure S17C, similar nucleotide dependent constriction and dilation also regulates the D2 pore loop 2, which is constricted in the ATP state (∼11 Å) and dilated in the ADP state (∼18 Å).

Collectively, our findings support the possibility that prior to UN recruitment, the D1 ring is initially at a partial ATP state (Figure 4). We hypothesize that initially the D2 ring would be in the ADP (open) state allowing the substrate to transverse and engage with the D2 ring (Figure S17). Only subsequently, does the D2 bind ATP and engages the “conveyor belt” translocation mechanism.^25^

**Figure 7:**
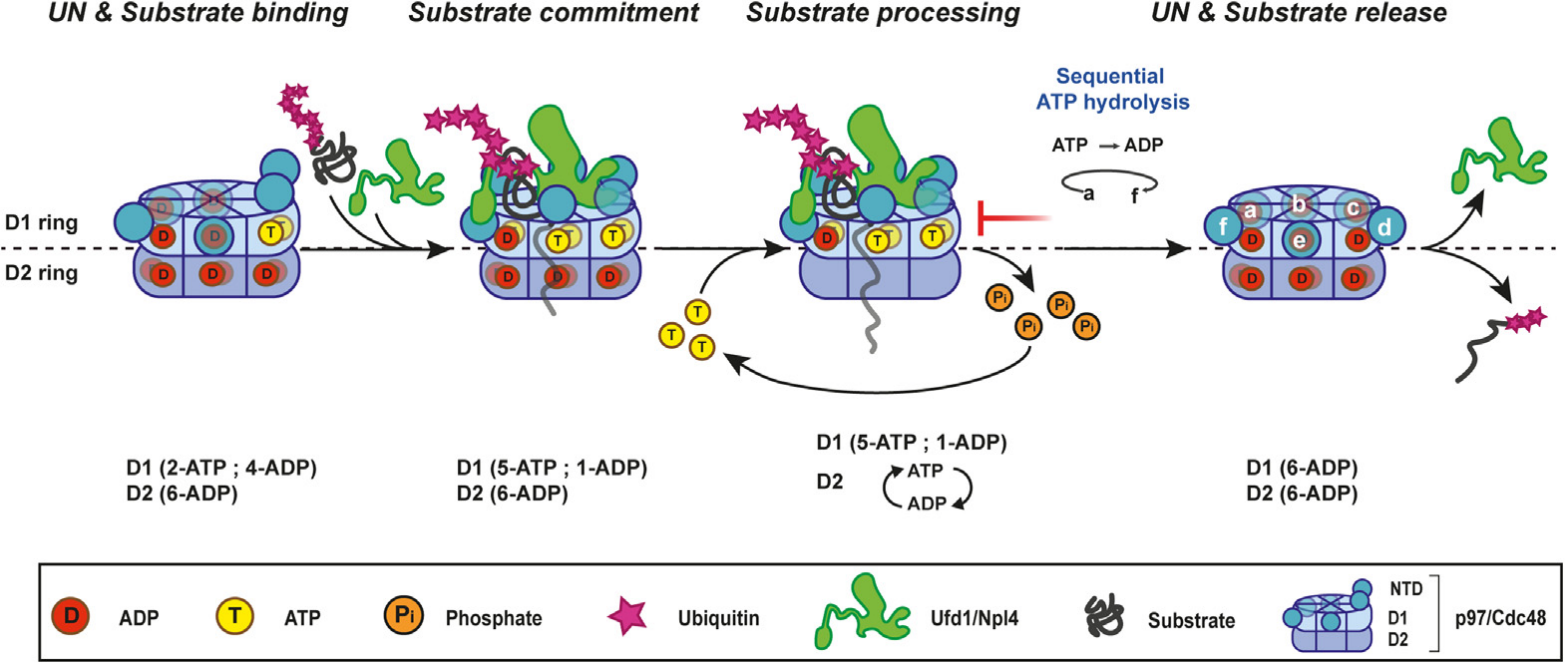
An updated model for UN-substrate recruitment and processing by p97/Cdc48 UN is recruited to p97 in which two of the D1 ring NTDs are in the ATP bound “up” conformation. Initially, upon association with UN the D2 ring protomers are ADP bound and the D2 ring pore is dilated, allowing the substrate to transverse and engage with the D2 ring. As UN binds p97, p97’s five NTDs shift to the “up” (ATP) state, while the “down” protomer associates with UBXL domain of Npl4, presumably directing substrate binding. Once ubiquitinated substrate is committed, D2 ring ATP binding and hydrolysis is activated, while the ATPase activity of D1 domain is suppressed, to avoid UN premature release. Multiple ATP binding and hydrolysis cycles promote substrate unfolding and translocation. Once substrate processing is completed, the D1 ring undergoes sequential ATP hydrolysis promoting substrate and UN release. p97 assumes its “ground” state in which two of the D1 ring protomers are in the ATP state, while the remaining four are ADP bound. In this state all six D2 ring protomers are ADP bound.

Our findings allow for the introduction of additional details to the previously proposed models for substrate processing by p97. As illustrated in Figure 7, we propose that p97 with two NTDs in the ATP “up” state engages with UN and poly-ubiquitinated substrates. The association entails interactions with multiple NTDs promoting a conformation in which five NTDs are in the “up” ATP state. Substrate recruitment is associated with extended D2 pore dimensions (D2 ring in the ADP state) presumably allowing for substrate entry.^25^^,^^34^ Substrate translocation is then promoted by ATP hydrolysis within the D2 ring, while ATP hydrolysis in the D1 ring is inhibited to prevent premature UN release. The reaction is terminated by substrate and cofactor (UN) release, which is mediated by ATP hydrolysis by D1 ring protomers (Figure 7).

The specific role of p97 in various cellular processes is defined by the assembly of many combinations of p97 with its numerous adaptors. Questions regarding the assembly of specific p97 complexes are still not resolved. In this regard UN is unique among the many p97 adaptors, as it is identified in combination with most p97 interactors and its binding to p97 is ATP dependent. Our findings favor a scenario in which the ATP dependent binding of UN to p97 mediates p97 nucleotide state to cofactors binding and function. This scenario would account for their indifference toward the nucleotide state and the presence of UN in most of p97 complexes.

### Limitations of the study

One limitation of the current study is the lack of a p97 substrate that would demonstrate the different steps in processing and unfolding. In fact, our p97 F539A behaves like a “cold sensitive” mutant as it becomes fully active only in elevated temperature (Figure 1F). This mutant may allow for the detection of individual steps along the substrate unfolding pathway, which can be achieved by performing cryo-EM analysis in the presence of the substrate at lower temperatures, using a substrate model system as was previously reported.^12^^,^^25^^,^^34^^,^^36^ Furthermore, as p97 F539A adopts a uniform conformation (two NTDs in the “up” and four NTDs in the “down” state, Figure 4) and has the capacity to complete its catalytic cycle upon incubation with its cofactor (UN) (Figure 6). It is appealing to use this property to synchronize substrate processing reactions and attempt to capture intermediate steps along the substrate processing pathway by p97. Notably, these research directions represent an important aspect in our future studies. An additional shortcoming of the current study is our inability to assign a specific density in our p97-UN complex to Ufd1, albeit rigorous attempts to detect the N-terminal domain of Ufd1, which is predicted to constitute a globular domain (Alphafold). These results are in alignment with previous reports that were able only to assign specific density to Npl4. In-light of these findings, an in-depth analysis of Ufd1 role in the p97-UN complex is appealing, as possible regulatory assembly cofactor role of Ufd1 may explain both the genetic and structural findings regarding Ufd1.

## STAR★Methods

### Key resources table

**Table:** 

REAGENT or RESOURCE	SOURCE	IDENTIFIER
**Antibodies**		

Rabbit polyclonal anti p97	Isakov et al.^50^	N/A
Mouse anti Actin	Abcam	Ab97023
Mouse monoclonal anti HA	Biolegend	Cat# MMS-101P (Clone 16B12)
Mouse monoclonal anti pGK	Abcam	Ab113687
Goat anti rabbit HRP conjugated	Jackson	RRID: AB_2313567
Goat anti mouse HRP conjugated	Jackson	RRID: AB_10015289

**Bacterial and virus strains**		

*Escherichia coli* M15	Qiagen	N/A
*Escherichia coli* BL21 (DE3)	Sigma-Aldrich	Cat# 69450
*Escherichia coli* TOP10	Thermo Fisher	Cat# C404010

**Chemicals, peptides, and recombinant proteins**		

Ampicillin	Sigma-Aldrich	Cat# A9393
Kanamycin sulfate	Sigma-Aldrich	Cat# K1876
Isopropyl-β-D-thiogalactopyranoside (IPTG)	Sigma-Aldrich	Cat# I1284
Lysozyme	Sigma-Aldrich	Cat# L6876
3,3'-dithiobis(sulfosuccinimidyl propionate (DTSSP)	Thermo Fisher	Cat# 21578
Β-mercaptoethanol	Thermo Fisher	Cat# 21985023
Dulbecco's Modified Eagle Medium (DMEM)	Sartorius	Cat# 01-055-1A
Fetal Bovine Serum (FBS) heat inactivated	Sartorius	Cat# 04-121-1A
Penicillin-streptomycin	Sartorius	Cat# 03-031-5B
L-glutamine	Sartorius	Cat# 03-020-1B
PEI MAX	Polysciences	Cat# 24766-1
Velcade (Bortezomib)	Selleck USA	Cat# S1013
CAM741	Besemer, J. et al.^1^	N/A
Phenylmethylsulfonyl fluoride (PMSF)	Sigma-Aldrich	Cat# P7626
Hygromycin B	Sigma-Aldrich	Cat# H0654
G418 sulfate	Sigma-Aldrich	Cat# G418-RO
Clarity Western ECL Substrate	BioRad	Cat# 1705061
Adenosine 5′-triphosphate disodium salt hydrate (ATP)	Sigma-Aldrich	Cat# A2383
Adenosine 5′-O-(3-Thiotriphosphate), Tetralithium Salt (ATPγS)	Sigma-Aldrich	Cat# 119120
3,3′,5,5′-Tetramethylbenzidine (TMB)	Thermo Fisher	Cat# N301
Recombinant p97 and variants	This paper	N/A
Recombinant Ufd1/Npl4	This paper	N/A
Recombinant Cdc48 and variants	This paper	N/A

**Critical commercial assays**		

Bicin Choninic Acid (BCA) Protein Assay Kit	Thermo Fisher	Cat# 23225

**Deposited data**		

p97 F266A coordinates and EM data	This paper	PDB: 8R0E; EMD-18790
p97 F539A coordinates and EM data	This paper	PDB: 8RSC; EMD-19476
p97 F266A F539A coordinates and EM data	This paper	PDB: 8RS9; EMD-19473
p97 F539A ADP state V coordinates and EM data	This paper	PDB: 8RSB; EMD-19475
p97 F539A state III coordinates and EM data	This paper	PDB: 8PQX; EMD-17827
p97 F539A-ATP EM data	This paper	EMD-17837
p97 F539A-adaptor EM data	This paper	EMD-18517
p97 F539A-adaptor cross-linking mass spectrometry data	This paper	PRIDE: PXD045179

**Experimental models: Cell lines**		

Human: HEK293T cells	ATCC	Cat# CRL-3216

**Experimental models: Organisms/strains**		

*Saccharomyces cerevisiae*: BY4742	GE Dharmacon	Cat# YSC1049
*Saccharomyces cerevisiae:* BY4742; *cdc48Δ*; pRS41H(HAX3)-Cdc48 and derivatives	This paper	N/A

**Oligonucleotides**		

Primer: Forward: KANMX insertion/CDC48 depletion: GTTGCATGACATCCCGGAC	Sigma-Aldrich	N/A
Primer: Reverse: KANMX insertion/CDC48 depletion: GGTCTGCGATTCCGACTC	Sigma-Aldrich	N/A
Primer: Forward: 266 Phe to Ala point mutation (p97): GAGCCTTCTTCTTTCTGATCAATGG	Sigma-Aldrich	N/A
Primer: Reverse: 266 Phe to Ala point mutation (p97): CAGTTTCATTTGCCACAGCTC	Sigma-Aldrich	N/A
Primer: Forward: 539 Phe to Ala point mutation (p97): CCAGGCTAACTTCATCTCCATCAAG	Sigma-Aldrich	N/A
Primer: Reverse: 539 Phe to Ala point mutation (p97): CATTCATTAGCAATGGCTTTG	Sigma-Aldrich	N/A
Primer: Forward: 276 Phe to Ala point mutation (Cdc48): CGGTGCCTTTTTTTTCTTAATTAATGG	Sigma-Aldrich	N/A
Primer: Reverse: 276 Phe to Ala point mutation (Cdc48): GTCTCATTAGCAACAGCTCTTGCC	Sigma-Aldrich	N/A
Primer: Forward: 549 Phe to Ala point mutation (Cdc48): TTTATCTCAGTCAAAGGTCCAG	Sigma-Aldrich	N/A
Primer: Reverse: 549 Phe to Ala point mutation (Cdc48): GTTGGCAGAAACCTCAGTAGCTAC	Sigma-Aldrich	N/A

**Recombinant DNA**		

Plasmid: pQE9-His-p97(QQ)	Ye et al.^2^	Addgene #14667
Plasmid: pQE9-His-p97(WT)	Meyer et al.^3^	Addgene #14666
Plasmid: pCDNA3.1-p97 and derivatives	This paper	N/A
Plasmid: pUG6SP-tTA'	Zilio et al.^4^	Addgene #41030
Plasmid: pRS41H-HA (X3)-cdc48 and derivatives	This paper	N/A
Plasmid: pET26-Ufd1-His	Bruderer et al.^5^	Addgene #21266
Plasmid: pET30-Npl4	Bruderer et al.^5^	Addgene #21267

**Software and algorithms**		

UCSF ChimeraX	Meng et al.^6^	https://www.cgl.ucsf.edu/chimerax/
Coot	Emsley and Cowtan^7^	https://www2.mrc-lmb.cam.ac.uk/personal/pemsley/coot/
Phenix	Afonine et al.^8^	https://phenix-online.org/documentation/reference/validation_cryo_em.html
RELION	Sjors H.W. Scheres^9^	https://www3.mrc-lmb.cam.ac.uk/relion//index.php/Main_Page
CRYOSPARC	Punjani et al.^10^	https://cryosparc.com/
PRISM	GraphPad Software	Version 8.4.2
Byonic	Protein Metrices	Version 4.6.1
MetaMorpheus	Lloyd M. Smith	https://bio.tools/MetaMorpheus
CRIMP2	Crowder et al.^11^	https://www.msstudio.ca/
Consurf	Landau et al.^12^	https://consurf.tau.ac.il/consurf_index.php
Biacore S200 Evaluation software	Cytiva	Version 1.1.1

**Other**		

Sensor chip CM5-Series S	Cytiva	Cat# BR100530

### Resource availability

#### Lead contact

Further information and requests for resources and reagents should be directed to and will be fulfilled by the lead contact, Prof. Ami Navon (ami.navon@weizmann.ac.il).

#### Materials availability

All Unique reagents generated in this study are available from the lead contact with a completed Materials Transfer Agreement.

#### Data and code availability

• The cross-linking MS data have been deposited to the ProteomeXchange Consortium via the PRIDE^51^ partner repository and are publicly available as of the data of publication. The dataset identifier is listed in the key resources table. EM maps and PDB coordinates have been deposited to the Protein Data Bank (PDB) repository and are publicly available as of the data of publication. Accession numbers are listed in the key resources table.
• This paper does not report original code.
• Any additional information required to reanalyze the data reported in this paper is available from the lead contact upon request.

### Experimental model and study participant details

#### Bacteria cultures

p97 phenylalanine mutants were generated by site-directed mutagenesis using the wild type His-p97/VCP as a template. In F266A or F539A mutant, a phenylalanine to alanine mutation was introduced in residues 266 in D1 or 539 in D2, respectively. For F266A F539A mutant with double mutations, F266A was used as a template in the site-directed mutagenesis system. All p97 constructs were cloned in a His-fusion expression vector, pQE9, and expressed as His-fusion protein in *Escherichia coli* strain M15 [pREP4] (QIAexpress system, Qiagen). The transformed cells were grown in 1L LB medium complemented with 100μg Ampicillin/ml and 100μg Kanamycin/ml for about 6 h at 37°C until OD_600nm_ reached 0.8. Protein expression was induced by addition of IPTG (0.5mM) and further grown for 5 h. The bacteria were then harvested by centrifugation (20,000 rpm, 10 min at 4°C). pET26-Ufd1 and pET30-Npl4 plasmids were kindly provided by Dr. Ariel Stanhill (Department of Natural and Life Sciences, The Open University of Israel) and confirmed by sequencing. Both Ufd1, a His-fusion protein (Ufd1-His), and Npl4, an untagged protein, were expressed in *Escherichia coli* BL21 (DE3) strain. The transformed cells were grown in 1L LB medium complemented with 100μg Kanamycin for about 5 h at 37°C until OD_600nm_ reached 0.8. Protein expression was induced by addition of IPTG (0.5mM) and further grown for 12 h at 16°C. The bacteria were then harvested by centrifugation (20,000 rpm, 10 min at 4°C).

#### Yeast strains and cultures

Yeast cells were grown in YPD (1% yeast extract, 2% peptone, 2% glucose) media and the indicated antibiotics were added: Hygromycin B (300 μg/ml) and G418 sulfate (300 μg/ml). The haploid yeast strain BY4742α was transfected with the yeast expression vector pRS41H, including the *CDC48* wild type or mutant (F276A, F549A F276A F549A) gene, *CDC48* promoter and terminator. Next, the *CDC48* gene on the chromosome of the haploid strain BY4742α, containing the pRS41H plasmid, was replaced by homologous recombination with the *KanMX* module, yielding the *cdc48Δ* strain with Hygromycin and G418 resistance. In this haploid strain, Cdc48 (wild type or mutant) is expressed under the control of the *CDC48* promoter. The insertion of *KANMX* cassette instead of *CDC48* gene was verified by colony PCR (Figure S3; Table S3). The list of the yeast strains used in this study is presented in Table S2.

#### Cell culture

HEK293T cells were grown in DMEM supplemented with 10% heat-inactivated fetal bovine serum, 1% penicillin-streptomycin and 1% L-glutamine at 37°C. Cells were grown up to 70–80% confluence for transient transfection. The cells were co-transfected with wild type His-p97/VCP or mutant (QQ, F266A, F539A and F266A F539A) and VCAM-1, which contains C’-terminal HA tag^22^ in a ratio of 1:2, respectively. The transient transfections were performed using the jetPEI reagent method. Where indicated, cells were treated with velcade (100nM) and/or CAM741 (250nM) overnight.

### Method details

#### Yeast plasmids and cloning

The *CDC48* gene including regions 250bp upstream and 250bp downstream was cloned from *S. cerevisiae* genomic DNA and inserted into yeast expression vector pRS41H. Plasmids expressing mutant forms of Cdc48, with an N’-terminal HAX3 tag, were made by site-directed mutagenesis. Additionally, a DNA cassette of *KANMX*, including the *TEF* promoter and terminator, was retrieved from multi-copy plasmid pUG6.

#### Purification of p97/VCP variants and its co-factor Ufd1/Npl4

The bacterial pellet was resuspended in lysis buffer (20mM HEPES pH 7.5, 300mM KCl, 10mM Imidazole), followed by lysozyme (1 mg/ml) incubation for 30 min at 4°C. The lysed cells were disrupted by sonication (100% amplitude, 30 sec on/off for 3 min), centrifuged at 20,000 rpm for 10 min at 4°C and filtered (0.45μm). The supernatant was purified on homemade Ni-affinity column using the Bio Logic Duo Flow (Bio-Rad). The protein was eluted with 20mM HEPES pH 7.5, 300mM KCl, 350mM Imidazole, and dialyzed against 20mM HEPES pH 7.5, 30mM KCl overnight at 4°C. The protein sample was then concentrated using a centrifugal filter device of 300KDa cutoff (Pall Corporation) and separated on size-exclusion chromatography (Superose 6 HR, Amersham Pharmacia) in 20mM HEPES pH 7.5, 30mM KCl, 1mM DTT. The purity of the eluted fractions was determined by SDS gel (10%) electrophoresis and Coomassie blue staining. Protein concentration was determined using absorbance spectroscopy at 280 nm. Aliquots of the protein were prepared and stored at −80°C. For the heterodimer complex Ufd1/Npl4, lysate containing a large excess of untagged Npl4 was mixed with lysate containing the His-fusion protein Ufd1 (Ufd1-His) in a ratio of 4:1, respectively. The complex was purified as described previously on Ni-affinity column, concentrated using a centrifugal filter device of 30KDa cutoff (Pall Corporation) and analyzed by SDS gel (10%) electrophoresis and Coomassie blue staining. Protein concentration was determined using absorbance spectroscopy at 280 nm. Aliquots of the protein were prepared and stored at −80°C. For the complex p97 F539A-UN, lysates of untagged p97 F539A and Npl4 were mixed with lysate containing the His-fusion protein Ufd1 (Ufd1-His). The complex was purified as described previously on Ni-affinity column followed by size exclusion chromatography (Superose 6 HR, Amersham Pharmacia) in 20mM HEPES pH 7.5, 30mM KCl, 1mM DTT. The purity of the eluted fractions was determined by SDS gel (10%) electrophoresis and Coomassie blue staining. Protein concentration was determined using absorbance spectroscopy at 280 nm.

#### ATPase activity assay

ATPase activity was measured by incubation of the different p97 variants (0.3μg for each variant) in p97 activity buffer (50mM HEPES pH 7.5, 5mM MgCl2, 30mM KCl, 2mM ATP, 1mM DTT) in the presence (1.2μg) and absence of Ufd1/Npl4 at 37°C for 20, 40, 60, 80 or 100 min. Ufd1/Npl4 complex was pre-incubated with p97 variants for 10 min prior the addition of 2mM ATP. p97 ATP hydrolysis was assayed by production of inorganic phosphate as described by Chifflet et al*.*^52^ The data is shown as mean ± S.D. of *n* ≥ 3 technical replicates. Significance was determined using the statistical ANOVA multiple comparisons analysis (F_(381)_ = 570, *p* value < 0.0001, α = 0.05: two-sided; For UN reactions: F_(8,130)_ = 329.6, *p* value < 0.0001, α = 0.05: two-sided).

#### Cryo-EM analysis

##### Sample preparation

A 3.5 μL of p97 phenylalanine mutant (F266A, F539A and F266A F539A) at 7 mg/ml in 50mM HEPES pH 7.5, 30mM KCl and 1mM DTT was applied on a freshly glow discharged Quntifiol holy carbon grids (R2/2, 200-mesh Cu grid; Protochips), blotted using a Vitrobot Mark IV (FEI company) for 3.5 seconds with 100% humidity at 4°C. The grids were plunged frozen in liquid ethane and stored in liquid nitrogen.

For the p97 F539A-UN complex samples: p97 F539A-UN complex (3.5 mg/ml), in 50mM HEPES pH 7.5, 30mM KCl and 1mM DTT, plunged immediately after size exclusion chromatography or incubated for 5 or 30 min at 37°C and centrifuged at 21,000Xg for 5 min to remove aggregates. Samples (3.5μL) freezing were carried out as p97 mutants.

##### Data acquisition

Data was acquired with a FEI Titan Krios transmission electron microscope (Thermofischer Scientific- FEI), operated at 300kV, using Gatan Microscopy Suite software (GMS). Movie frames were recorded at a magnification of 105,000X, corresponding to a magnified pixel size of 0.86 Å or 0.793 Å in Fringeless mode, using a K3 Summit direct electron detector camera (Gatan). BioQuantum energy filter (Gatan) was operated with an energy slit width of 20 eV. The exposure rate was in a range of ∼25–28 electrons/Å^2^/sec and the total exposure time was 1.49 sec while intermediate frames were recorded in 0.033 sec intervals resulting in an accumulated dose of ∼38–42 electrons per Å^2^ and a total of 45 frames per micrograph with defocus values ranging from −0.5 μm to −2.0μm (Table S1). Automatic data acquisition was done using EPU (Thermofischer Scientific - FEI).

##### Image processing, classification and refinement

Cryo-EM micrograph frames were aligned using beam-induced motion correction (MotionCor2) and the contrast transfer function (CTF) for the aligned frames was determined with CTFFIND4.^53^ Micrographs with non-decent FFT thon rings were discarded. All subsequent processing was performed using RELION 3.1.2.^54^ Particle picking was performed by manually choosing ∼2,000 particles and generating templates through reference-free two-dimensional (2D) classification, followed by automatic template-based picking. False-positive particles or particles classified in poorly defined classes were discarded after multiple iterations of 2D classification. Multiple rounds of 3D classifications were performed on a binned dataset (factor 2: pixel size of 1.72 Å or 1.586 Å), with different number of classes, with the previously reported p97 structure as the reference model (PDB ID:5FTN).^11^ For p97 F539A-adaptor complex the reference model used for 3D classification was EMD-20730. In the initial 3D classification C1 symmetry was applied and particles classified into classes with high symmetry were then refined with C6. Particles from homogeneous groups showing similar architecture with well-resolved features were unbinned (pixel size 0.86 Å or 0.793 Å) and subjected to 3D refinement. For the selection of a better conformational homogeneous population, further 3D sorting was performed. Finally, the sorted particles were used for final map reconstruction, where 3D refinement process was followed by-particle CTF refinement and Bayesian polishing implemented in RELION 3.1.2 or in cryoSPARC 3.2.^55^ The detailed data processing workflows are shown in Supplementary Figures S5, S7, S9, S10, S12, S14 and S15. Reported resolutions are based on Fourier shell correlation (FSC) using the gold standard FSC = 0.143 criterion. Figures of the 3D maps were prepared using USCF ChimeraX.

##### Model building, refinement and validation

Model building was based on the existing cryo-EM structure of human p97 (PDB ID:7BPA). The models were docked into the EM density maps using UCSF Chimera followed by further adjustment using Coot.^56^ Sharpened maps were used for well-resolved regions while individual residues were manually adjusted and fit into the density, in oppose to flexible regions whereas the unsharpened maps were used. The final model was subjected to global refinement and minimization in real space using phenix.real_space_refine module implemented in Phenix.^57^^,^^58^ Model geometry validation was performed using Molprobity analysis tool.^59^ The parameters of the models refinement are provided in Table S1.

#### Affinity assay (ELISA)

NUNC Maxisorp flat bottom, non-tissue culture 96-well plates were coated with 100ng/well of Ufd1/Npl4 complex in PBS buffer (20mM sodium phosphate pH 7.4, 150mM NaCl) and incubated overnight at 4°C. The wells were washed three times with TBST buffer (50mM Tris-HCl pH 7.5, 100mM NaCl, 0.005% Tween-20) and blocked for 3 hours at room temperature with 5% milk in TBST. Next, the wells were washed again with TBST (X3) and p97 variants were added at different concentrations (0, 2,5,10,30,50,100,200,400 and 500nM) in a total volume of 100μL/well in binding buffer (25mM HEPES pH 7.5, 100mM KCl, 3mM MgCl_2_, 1mM DTT) in the absence or presence of ATPγS (2mM). The plate was incubated at room temperature for 30 min, followed by TBST washes (X5). Primary antibody against p97 was added (100μL/well, 1:5,000) and the plate was incubated for 1 h in room temperature, followed by TBST (X5) washes. Thereafter, secondary antibody conjugated to horseradish peroxidase (HRP) (100μL /well, 1:10,000) was applied in each well and the plate was incubated for 1 h in room temperature. After washing with TBST (x5), 50μL of the substrate TMB (Thermo-Fisher) was added and color development was measured at 370 nm and 652 nm at intervals of 60 sec for 15–20 min. The data is shown as mean ± S.D. of *n* ≥ 3 technical replicates. The data was fitted using the Hill equation and the goodness of the fit was evaluated by R^2^ (Table S4).

#### SPR binding assay

The SPR assays were performed at 25°C on a Biacore S200 instrument (GE HealthCare). p97 WT (in 50mM HEPES pH 7.5, 150mM KCl, 2mM MgCl_2_, 2mM ATP and 0.005% (v/v) Tween 20) or p97 F539A (in 50mM HEPES pH 7.5, 150mM KCl and 0.005% (v/v) Tween 20) were immobilized via amine coupling to the carboxy-methyl dextran surface of a Biacore CM5 sensor chip (Series S, cytiva). Surfaces were activated for 7.5 min with a mixture of 0.5M 1-ethyl-3-(3-dimethylpropyl)-carbodiimide & 0.1M N-hydroxysuccinimide to convert surface carboxyl groups into an amine reactive ester before the injection of the ligand (p97 WT or mutant) to be immobilized. Immobilization was performed with mixture of 0.15M Sodium Acetate pH 4.5 and p97 protein (0.06 mg/mL) using a flow-rate of 10 μL/min, whereas the surface was exposed until the signal was about 1200 RU. The control channel was treated in an identical way, omitting the injection of the ligand (p97). Following ligand immobilization, surfaces were blocked using 1M ethanolamine pH 8.0 for 5 min with a flow-rate of 10 μL/min. Next, UN (analyte) in different concentrations (1, 2.5, 5, 10, 20, 40, 60, 80, and 100 nM) was injected (30 μL/min) over the reference and immobilized p97 (WT or mutant) in a single cycle method (overall 5 cycles), while each concentration was exposed to the surfaces for 120 sec and allowed to dissociate for 1500 sec after the injection of the highest concentration. Sensorgrams obtained from the experiments were referenced with the in-line reference cell, to subtract bulk effects and non-specific interactions and with buffer injections, and were analyzed using the Biacore S200 Evaluation Software 1.1.1 (GE HealthCare). The data was fitted using two state reaction model implemented in the Biacore S200 Evaluation Software 1.1.1 (GE HealthCare) and the goodness of the fit was evaluated by χ^2^ (p97 wild type: χ^2^ = 2.01, p97 F539A: χ^2^ = 5.24).

#### Stability assay (Thermal Shift Assay)

The stability of p97 variants was measured by Tycho (nanoTEMPER) instrument with a temperature range of 35°C–95°C. Prior to capillary loading, 10 μL of each variant (1 mg/ml) was pre-incubated for 15 min in the presence (2mM) or absence of ATPγS in 50mM HEPES pH 7.5 and 30mM KCl. The data is shown as mean ± S.D. of *n* ≥ 3 technical replicates. Significance was determined using the statistical ANOVA multiple comparisons analysis (F_(7,16)_ = 250.7, *p* value < 0.0001, α = 0.05: two-sided).

#### Western Blot analysis

Protein samples were boiled for 5 min in 1X Laemmli sample buffer (10.4mM Tris-HCl pH 6.8, 10% glycerol, 0.02% bromophenol blue, 1mM DTT) separated on a 10% reducing SDS polyacrylamide gel and transferred onto PVDF membrane (0.45μm). The membrane was blocked for 20 min at room temperature in 5% milk in TBST (50mM Tris-HCl pH 7.5, 100mM NaCl, 0.005% Tween-20), washed four times with TBST (4X5 min) and incubated for 2 h with the indicated antibody (anti HA, anti p97, anti Actin and anti pGK). Thereafter, the membrane was washed again with TBST (4X5 min) and incubated for 1 h with a secondary antibody conjugated to horseradish peroxidase (HRP). The membrane was developed using homemade chemiluminescence reagent and the signal was acquired by Bio-Rad ChemiDoc XRS while verifying that none of the quantified bands were in saturation. Image Lab software (Biorad laboratories) was used for quantitative analysis.

#### Cell culture DNA constructs, mutagenesis, lysis and protein purification

The wild-type His-p97/VCP, QQ mutant and VCAM-1 plasmids were previously described.^22^ All plasmids were confirmed by sequencing. The phenylalanine mutants were generated as previously described for p97 bacterial expression vectors using pcDNA3.1-His-p97/VCP as a template. The mutations were verified by DNA sequencing. The cells were lysed in cold TNH buffer (20mM HEPES pH 7.5, 100mM NaCl, 1%Triton X-100, 1mM EDTA, 1.5mM MgCl2, 1mM DTT, 1:1,000 protease inhibitors), incubated on ice for 15 min and clarified by centrifugation (14,000 rpm, 15 min at 4°C). Protein concentration of the cell lysates was determined using Bicin Choninic Acid protein assay reagents (Pierce, Rockford, IL).

#### Yeast protein extraction and western blots

1.5mL of cell cultures at OD_600nm_ 1.0–1.5 were collected and harvested by centrifugation (1500Xg, 3 min). The pellet was snapped freeze in liquid nitrogen, followed by resuspension with 300μL buffer A (1.85M NaOH, 7% β-mercaptoethanol) and vortexed for 1 min. Next, 150μL of 100% TCA was added and the tubes were inverted twice and incubated on ice for 5 min. The samples were centrifuged (10 min at 4°C) and the pellet was resuspended with 450μL non-titrated Tris 1M. Thereafter, the cells were centrifuged (10 min at 4°C) again and the pellet was resuspended with 150μL buffer B (2.5% SDS, 5mM EDTA pH 8.0, 1mM PMSF). The suspended cells were frozen at −20°C for 30min, followed by 5 min incubation at 65°C. The samples were centrifuged (5 min, 14,000rpm) and the supernatants were used as cell lysates. Protein concentrations were determined using Bicin Choninic Acid protein assay reagents (Pierce, Rockford, IL) and adjusted to the same concentrations with buffer B. 80μg of total proteins in the lysates were resolved by 10% SDS-PAGE and proteins were transferred to PVDF membranes for western blotting with anti-HA and anti-pGK antibodies.

#### Yeast growth assay

For YPD medium growth assay, 2mL of cell cultures at OD_600nm_ 0.6–0.8 were collected and diluted to OD_600nm_ 0.1 in a total volume of 150μL into a tissue culture treated 96-well plate with flat bottom and lid. Specific wells were inoculated with medium only for background correction purposes. OD_600nm_ measurements were taken by plate reader (Electron Coeporation, Thermo) at intervals of 30 min for 15–20 h at the indicated temperatures (25°C, 30°C and 37°C), while the plate was shaken between those intervals (speed 180 spm, diameter 25 mm). The data is shown as mean ± S.D. of *n* ≥ 3 technical replicates. The data was fitted using logistic model and the goodness of the fit was evaluated by R^2^ (Table S5).

#### Double mutant cycle

For double mutant cycle calculations, the formula of Gibbs free energy was used: $\Delta G=-RTln\left(K_{d}\right)$, where R (cal/K∗mol) is the gas constant, T is the temperature (K) and Kd is the disassociation constant from binding affinity assay (Figure 2A). The effect of each point mutation, associated with structural or functional property, in the presence of UN was calculated by the difference between wild type’s and mutant’s Gibbs free energy: ${\Delta \Delta G}^{mut-wt\;}=\left(-RTln\left(K_{d\;\left[mut\right]}\right)\right)-\left(-RTln\left(K_{d\;\left[wt\right]}\right)\right)=RTln\left(\frac{K_{d\;\left[wt\right]}}{K_{d\;\left[mut\right]}}\right)$.^38^^,^^39^

#### Cross-linking and LC-MS/MS analysis

##### Sample preparation

Purified p97 F539A (1.26 mg/mL) and its adaptor (0.66 mg/mL) in 50mM HEPES pH 7.5 and 30mM KCl were cross-linked with 10, 50 or 100 μM of DTSSP for 30 min at room temperature. The reactions were quenched by addition of Tris-HCl pH 7.5 to a final concentration of 50 mM, following incubation for 15 min at room temperature. Samples were incubated in 8M urea for 30 min at 25°C, followed by a dilution to 1.6M urea with 50mM ammonium bicarbonate and subjected to digestion with trypsin (Promega; Madison, WI, USA) overnight at 37°C at 50:1 protein:trypsin ratio, followed by a second trypsin digestion for 4 h. The digestions were stopped by addition of trifluroacetic acid (1% final concentration). Following digestion, peptides were desalted using Oasis HLB, μElution format (Waters, Milford, MA, USA). The samples were vacuum dried and stored in −20°C until further analysis.

##### Liquid chromatography

ULC/MS grade solvents were used for all chromatographic steps. Each sample was loaded using split-less nano-Ultra Performance Liquid Chromatography (10 kpsi nanoAcquity; Waters, Milford, MA, USA). The mobile phase was: A) H2O + 0.1% formic acid and B) acetonitrile +0.1% formic acid. Desalting of the samples was performed online using a reversed-phase Symmetry C18 trapping column (180 μm internal diameter, 20 mm length, 5 μm particle size; Waters). The peptides were then separated using a T3 HSS nano-column (75 μm internal diameter, 250 mm length, 1.8 μm particle size; Waters) at 0.35 μL/min. Peptides were eluted from the column into the mass spectrometer using the following gradient: 4% to 40%B in 55 min, 40% to 90%B in 5 min, maintained at 90% for 5 min and then back to initial conditions.

##### Mass spectroscopy

The nanoUPLC was coupled online through a nanoESI emitter (10 μm tip; New Objective; Woburn, MA, USA) to a quadrupole orbitrap mass spectrometer (Q Exactive HF, Thermo Scientific) using a FlexIon nanospray apparatus (Proxeon). Data was acquired in data dependent acquisition (DDA) mode, using a Top10 method. MS1 resolution was set to 120,000 (at 200m/z), mass range of 375-1650m/z, AGC of 3e6 and maximum injection time was set to 60msec. MS2 resolution was set to 15,000, quadrupole isolation 1.7m/z, NCE 27 or 35, AGC of 1e5, dynamic exclusion of 20sec and maximum injection time of 60 msec.

##### Data analysis

The data was searched against the *E. coli* SwissProt proteome (January 2023 version, 4448 entries) appended with a list of common lab contaminants, human p97 and rat Npl4 in Byonic (ProteinMetrices, version 4.6.1). A focused database was created and used for further analysis with the crosslinker DTSSP was defined on K. Only crosslinked peptides with a positive xlinkx score were considered. Additional analysis was conducted using MetaMorpheus v1.0.2, where the crosslinker was allowed to bind to K, S or T. The data was filtered to keep peptide pairs that had an XL total score>10. Analysis in CRIMP2 was done using a database of the 10 proteins with the highest PSM number of the focused database and linker sites of K, S and T. All the candidate identifications were manually inspected before accepting the identification as valid.

### Quantification and statistical analysis

All statistical analyses were performed in Prism (GraphPad) software (version 8.4.2). The statistical test for each experiment is described in details in the methods details section including number of replicates, mean ± STD and the goodness of the fit value. The significance was defined as follows: P-values higher than 0.05 (*p* > 0.05) are summarized withs "ns", P-values smaller than 0.05 (*p* ≤ 0.05) are summarized with one asterisk, P-values smaller than 0.01 (*p* ≤ 0.01) are summarized with two asterisks, P-values smaller than 0.001 (*p* ≤ 0.001) are summarized with three asterisks and P-values smaller than 0.0001 (*p* ≤ 0.0001) are summarized with four asterisks.

## Supplemental information

Document S1. Figures S1‒S17 and Tables S1‒S5

## References

[1] Xia D., Tang W.K., Ye Y. (2016). Structure and function of the AAA+ ATPase p97/Cdc48p. Gene.

[2] Ye Y., Meyer H.H., Rapoport T.A. (2001). The AAA ATPase Cdc48/p97 and its partners transport proteins from the ER into the cytosol. Nature.

[3] Dai R.M., Li C.C. (2001). Valosin-containing protein is a multi-ubiquitin chain-targeting factor required in ubiquitin-proteasome degradation. Nat. Cell Biol..

[4] Bodnar N., Rapoport T. (2017). Toward an understanding of the Cdc48/p97 ATPase. F1000Res..

[5] Davies J.M., Brunger A.T., Weis W.I. (2008). Improved structures of full-length p97, an AAA ATPase: implications for mechanisms of nucleotide-dependent conformational change. Structure.

[6] Meyer H., Bug M., Bremer S. (2012). Emerging functions of the VCP/p97 AAA-ATPase in the ubiquitin system. Nat. Cell Biol..

[7] Stolz A., Hilt W., Buchberger A., Wolf D.H. (2011). Cdc48: a power machine in protein degradation. Trends Biochem. Sci..

[8] Yeung H.O., Kloppsteck P., Niwa H., Isaacson R.L., Matthews S., Zhang X., Freemont P.S. (2008). Insights into adaptor binding to the AAA protein p97. Biochem. Soc. Trans..

[9] Hanzelmann P., Schindelin H. (2017). The Interplay of Cofactor Interactions and Post-translational Modifications in the Regulation of the AAA+ ATPase p97. Front. Mol. Biosci..

[10] Baek G.H., Cheng H., Choe V., Bao X., Shao J., Luo S., Rao H. (2013). Cdc48: a swiss army knife of cell biology. J. Amino Acids.

[11] Banerjee S., Bartesaghi A., Merk A., Rao P., Bulfer S.L., Yan Y., Green N., Mroczkowski B., Neitz R.J., Wipf P. (2016). 2.3 Å resolution cryo-EM structure of human p97 and mechanism of allosteric inhibition. Science.

[12] Bodnar N.O., Rapoport T.A. (2017). Molecular Mechanism of Substrate Processing by the Cdc48 ATPase Complex. Cell.

[13] Song C., Wang Q., Li C.C.H. (2003). ATPase activity of p97-valosin-containing protein (VCP). D2 mediates the major enzyme activity, and D1 contributes to the heat-induced activity. J. Biol. Chem..

[14] Ye Y., Meyer H.H., Rapoport T.A. (2003). Function of the p97-Ufd1-Npl4 complex in retrotranslocation from the ER to the cytosol: dual recognition of nonubiquitinated polypeptide segments and polyubiquitin chains. J. Cell Biol..

[15] Wang Q., Song C., Li C.C.H. (2003). Hexamerization of p97-VCP is promoted by ATP binding to the D1 domain and required for ATPase and biological activities. Biochem. Biophys. Res. Commun..

[16] Majumder P., Rudack T., Beck F., Danev R., Pfeifer G., Nagy I., Baumeister W. (2019). Cryo-EM structures of the archaeal PAN-proteasome reveal an around-the-ring ATPase cycle. Proc. Natl. Acad. Sci. USA.

[17] de la Pena A.H., Goodall E.A., Gates S.N., Lander G.C., Martin A. (2018). Substrate-engaged 26S proteasome structures reveal mechanisms for ATP-hydrolysis-driven translocation. Science.

[18] Huang R., Ripstein Z.A., Rubinstein J.L., Kay L.E. (2020). Probing Cooperativity of N-Terminal Domain Orientations in the p97 Molecular Machine: Synergy Between NMR Spectroscopy and Cryo-EM. Angew. Chem. Int. Ed. Engl..

[19] Cayli S., Klug J., Chapiro J., Fröhlich S., Krasteva G., Orel L., Meinhardt A. (2009). COP9 signalosome interacts ATP-dependently with p97/valosin-containing protein (VCP) and controls the ubiquitination status of proteins bound to p97/VCP. J. Biol. Chem..

[20] Wang H.F., Shih Y.T., Chen C.Y., Chao H.W., Lee M.J., Hsueh Y.P. (2011). Valosin-containing protein and neurofibromin interact to regulate dendritic spine density. J. Clin. Invest..

[21] Lee J.H., Kwon J.H., Jeon Y.H., Ko K.Y., Lee S.R., Kim I.Y. (2014). Pro178 and Pro183 of selenoprotein S are essential residues for interaction with p97(VCP) during endoplasmic reticulum-associated degradation. J. Biol. Chem..

[22] Braunstein I., Zach L., Allan S., Kalies K.U., Stanhill A. (2015). Proteasomal degradation of preemptive quality control (pQC) substrates is mediated by an AIRAPL-p97 complex. Mol. Biol. Cell.

[23] Xue L., Blythe E.E., Freiberger E.C., Mamrosh J.L., Hebert A.S., Reitsma J.M., Hess S., Coon J.J., Deshaies R.J. (2016). Valosin-containing protein (VCP)-Adaptor Interactions are Exceptionally Dynamic and Subject to Differential Modulation by a VCP Inhibitor. Mol. Cell. Proteomics.

[24] Vaz B., Halder S., Ramadan K. (2013). Role of p97/VCP (Cdc48) in genome stability. Front. Genet..

[25] Twomey E.C., Ji Z., Wales T.E., Bodnar N.O., Ficarro S.B., Marto J.A., Engen J.R., Rapoport T.A. (2019). Substrate processing by the Cdc48 ATPase complex is initiated by ubiquitin unfolding. Science.

[26] Sato Y., Tsuchiya H., Yamagata A., Okatsu K., Tanaka K., Saeki Y., Fukai S. (2019). Structural insights into ubiquitin recognition and Ufd1 interaction of Npl4. Nat. Commun..

[27] Pye V.E., Beuron F., Keetch C.A., McKeown C., Robinson C.V., Meyer H.H., Zhang X., Freemont P.S. (2007). Structural insights into the p97-Ufd1-Npl4 complex. Proc. Natl. Acad. Sci. USA.

[28] Chia W.S., Chia D.X., Rao F., Bar Nun S., Geifman Shochat S. (2012). ATP binding to p97/VCP D1 domain regulates selective recruitment of adaptors to its proximal N-domain. PLoS One.

[29] Bodnar N.O., Kim K.H., Ji Z., Wales T.E., Svetlov V., Nudler E., Engen J.R., Walz T., Rapoport T.A. (2018). Structure of the Cdc48 ATPase with its ubiquitin-binding cofactor Ufd1-Npl4. Nat. Struct. Mol. Biol..

[30] Hanzelmann P., Schindelin H. (2016). Characterization of an Additional Binding Surface on the p97 N-Terminal Domain Involved in Bipartite Cofactor Interactions. Structure.

[31] Huang R., Ripstein Z.A., Rubinstein J.L., Kay L.E. (2019). Cooperative subunit dynamics modulate p97 function. Proc. Natl. Acad. Sci. USA.

[32] Ashkenazy H., Abadi S., Martz E., Chay O., Mayrose I., Pupko T., Ben-Tal N. (2016). ConSurf 2016: an improved methodology to estimate and visualize evolutionary conservation in macromolecules. Nucleic Acids Res..

[33] Besemer J., Harant H., Wang S., Oberhauser B., Marquardt K., Foster C.A., Schreiner E.P., de Vries J.E., Dascher-Nadel C., Lindley I.J.D. (2005). Selective inhibition of cotranslational translocation of vascular cell adhesion molecule 1. Nature.

[34] Pan M., Yu Y., Ai H., Zheng Q., Xie Y., Liu L., Zhao M. (2021). Mechanistic insight into substrate processing and allosteric inhibition of human p97. Nat. Struct. Mol. Biol..

[35] Schuller J.M., Beck F., Lössl P., Heck A.J.R., Förster F. (2016). Nucleotide-dependent conformational changes of the AAA+ ATPase p97 revisited. FEBS Lett..

[36] Blythe E.E., Gates S.N., Deshaies R.J., Martin A. (2019). Multisystem Proteinopathy Mutations in VCP/p97 Increase NPLOC4.UFD1L Binding and Substrate Processing. Structure.

[37] Hanzelmann P., Buchberger A., Schindelin H. (2011). Hierarchical binding of cofactors to the AAA ATPase p97. Structure.

[38] Horovitz A. (1996). Double-mutant cycles: a powerful tool for analyzing protein structure and function. Fold. Des..

[39] Horovitz A., Fleisher R.C., Mondal T. (2019). Double-mutant cycles: new directions and applications. Curr. Opin. Struct. Biol..

[40] Bebeacua C., Förster A., McKeown C., Meyer H.H., Zhang X., Freemont P.S. (2012). Distinct conformations of the protein complex p97-Ufd1-Npl4 revealed by electron cryomicroscopy. Proc. Natl. Acad. Sci. USA.

[41] Cooney I., Han H., Stewart M.G., Carson R.H., Hansen D.T., Iwasa J.H., Price J.C., Hill C.P., Shen P.S. (2019). Structure of the Cdc48 segregase in the act of unfolding an authentic substrate. Science.

[42] Zhang X., Shaw A., Bates P.A., Newman R.H., Gowen B., Orlova E., Gorman M.A., Kondo H., Dokurno P., Lally J. (2000). Structure of the AAA ATPase p97. Mol. Cell.

[43] van den Boom J., Marini G., Meyer H., Saibil H.R. (2023). Structural basis of ubiquitin-independent PP1 complex disassembly by p97. EMBO J..

[44] Pan M., Zheng Q., Yu Y., Ai H., Xie Y., Zeng X., Wang C., Liu L., Zhao M. (2021). Seesaw conformations of Npl4 in the human p97 complex and the inhibitory mechanism of a disulfiram derivative. Nat. Commun..

[45] Twomey E.C. (2021). Aboard the ISS: intersubunit signaling revealed in the p97 ATPase. Nat. Struct. Mol. Biol..

[46] Zhao M., Wu S., Zhou Q., Vivona S., Cipriano D.J., Cheng Y., Brunger A.T. (2015). Mechanistic insights into the recycling machine of the SNARE complex. Nature.

[47] Gao H., Li F., Ji Z., Shi Z., Li Y., Yu H. (2022). Cryo-EM structures of human p97 double hexamer capture potentiated ATPase-competent state. Cell Discov..

[48] Xu Y., Han H., Cooney I., Guo Y., Moran N.G., Zuniga N.R., Price J.C., Hill C.P., Shen P.S. (2022). Active conformation of the p97-p47 unfoldase complex. Nat. Commun..

[49] Philip V., Harris J., Adams R., Nguyen D., Spiers J., Baudry J., Howell E.E., Hinde R.J. (2011). A survey of aspartate-phenylalanine and glutamate-phenylalanine interactions in the protein data bank: searching for anion-pi pairs. Biochemistry.

[50] Isakov E., Stanhill A. (2011). Stalled proteasomes are directly relieved by P97 recruitment. J. Biol. Chem..

[51] Perez-Riverol Y., Bai J., Bandla C., García-Seisdedos D., Hewapathirana S., Kamatchinathan S., Kundu D.J., Prakash A., Frericks-Zipper A., Eisenacher M. (2022). The PRIDE database resources in 2022: a hub for mass spectrometry-based proteomics evidences. Nucleic Acids Res..

[52] Chifflet S., Torriglia A., Chiesa R., Tolosa S. (1988). A method for the determination of inorganic phosphate in the presence of labile organic phosphate and high concentrations of protein: application to lens ATPases. Anal. Biochem..

[53] Rohou A., Grigorieff N. (2015). CTFFIND4: Fast and accurate defocus estimation from electron micrographs. J. Struct. Biol..

[54] Kimanius D., Dong L., Sharov G., Nakane T., Scheres S.H.W. (2021). New tools for automated cryo-EM single-particle analysis in RELION-4.0. Biochem. J..

[55] Punjani A., Rubinstein J.L., Fleet D.J., Brubaker M.A. (2017). cryoSPARC: algorithms for rapid unsupervised cryo-EM structure determination. Nat. Methods.

[56] Emsley P., Cowtan K. (2004). Coot: model-building tools for molecular graphics. Acta Crystallogr. D Biol. Crystallogr..

[57] Liebschner D., Afonine P.V., Baker M.L., Bunkóczi G., Chen V.B., Croll T.I., Hintze B., Hung L.W., Jain S., McCoy A.J. (2019). Macromolecular structure determination using X-rays, neutrons and electrons: recent developments in Phenix. Acta Crystallogr. D Struct. Biol..

[58] Afonine P.V., Poon B.K., Read R.J., Sobolev O.V., Terwilliger T.C., Urzhumtsev A., Adams P.D. (2018). Real-space refinement in PHENIX for cryo-EM and crystallography. Acta Crystallogr. D Struct. Biol..

[59] Chen V.B., Arendall W.B., Headd J.J., Keedy D.A., Immormino R.M., Kapral G.J., Murray L.W., Richardson J.S., Richardson D.C. (2010). MolProbity: all-atom structure validation for macromolecular crystallography. Acta Crystallogr. D Biol. Crystallogr..

