# Supplementary material for: A non-symmetrical p97 conformation initiates a multistep recruitment of Ufd1/Npl4
Source: iScience. 2024 May 21;27(6):110061. doi: 10.1016/j.isci.2024.110061 (PMC11214410; doi:10.1016/j.isci.2024.110061)
Supplement: Document S1. Figures S1‒S17 and Tables S1‒S5 [file mmc1.pdf]

iScience, Volume *27*

Supplemental information

A non-symmetrical p97 conformation initiates a multistep recruitment of Ufd1/Npl4

Michal Arie, Donna Matzov, Rotem Karmona, Natalia Szenkier, Ariel Stanhill, and Ami Navon

Supplemental Information

Fig. S1.

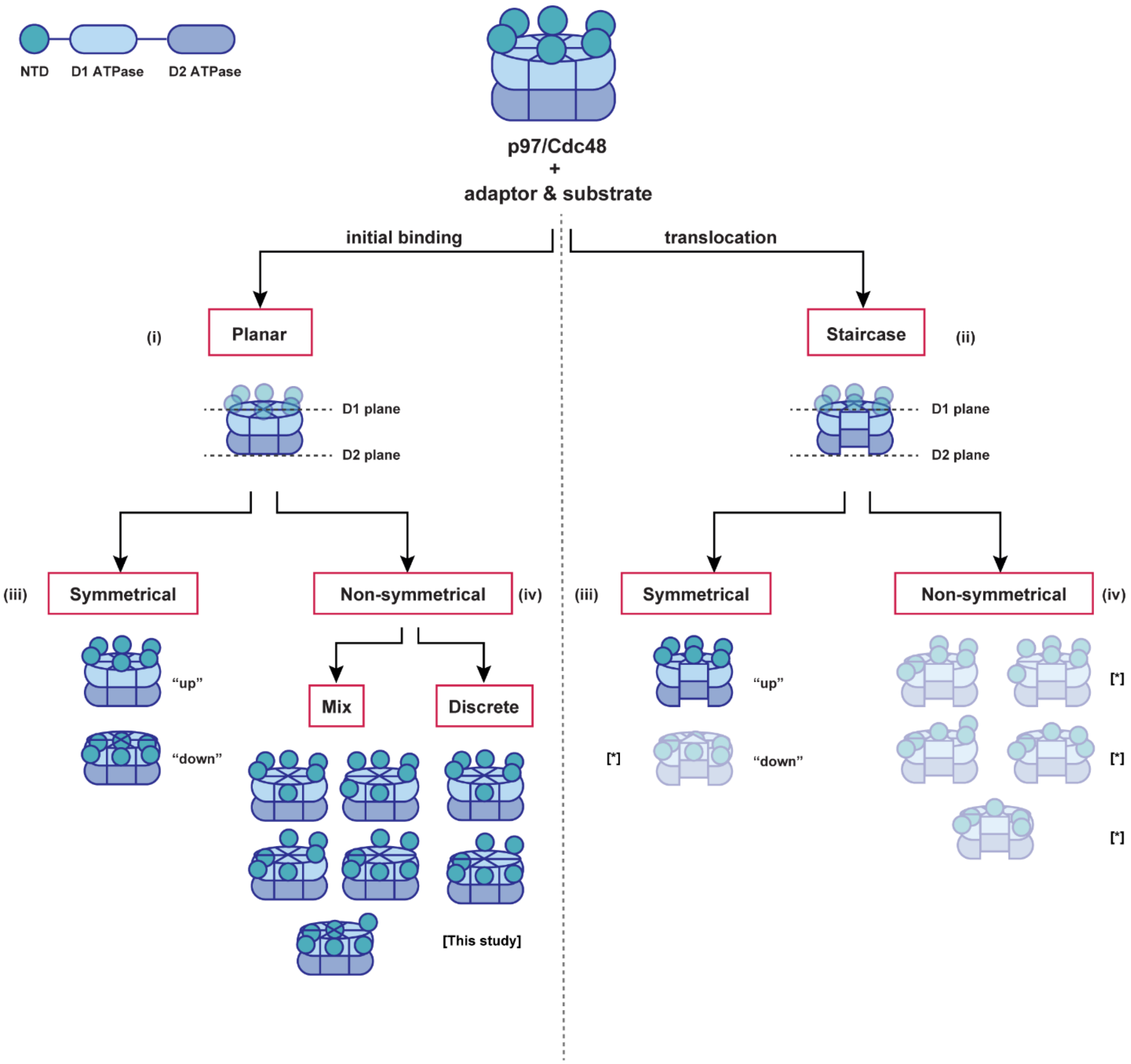

<u>Figure S1:</u> p97/Cdc48 arrangement in the presence and absence of substrate, related to Fig. 3-4. The different domains of p97/Cdc48 are marked as follows: NTD in blue, D1 ATPase ring in light blue and the D2 ATPase ring in purple. Prior to substrate translocation, p97/Cdc48 by itself or in the presence of adaptor/substrate, assumes a planar configuration **(i)** with respect to the central channel (all six protomers are in the same plane). p97/Cdc48's NTDs can adopt symmetrical conformation **(iii)** of either "up" (ATP) state or "down" (ADP) state [1-4]. Alternatively, a non-symmetrical conformation **(iv)** is composed from multiple combinations of "up" and "down" states. The different non-symmetrical conformations were described previously [5] and were obtained concurrently, while originating from the same cryo-EM dataset, demonstrating the non-uniformity of p97/Cdc48 particles (mixture of sub-populations). In contrast, each of the two sub-populations: two NTDs in the "up" state, as well as the five NTDs in the "up" state were obtained as a homogeneous population, stabilized by p97 F539A mutant in the absence or presence of its adaptor

respectively, capturing discrete states of p97/Cdc48. When substrate is translocated, p97/Cdc48 adopts the staircase configuration **(ii)**, in which one of the protomers is shifted from the other protomers' plane. In this scenario the NTDs of p97/Cdc48 can be arranged in symmetrical [6-11] **(iii)** or non-symmetrical **(iv)** conformation. Sub-populations that have not yet been structurally determined are depicted as translucent and denoted by an asterisk (*).

Fig. S2.

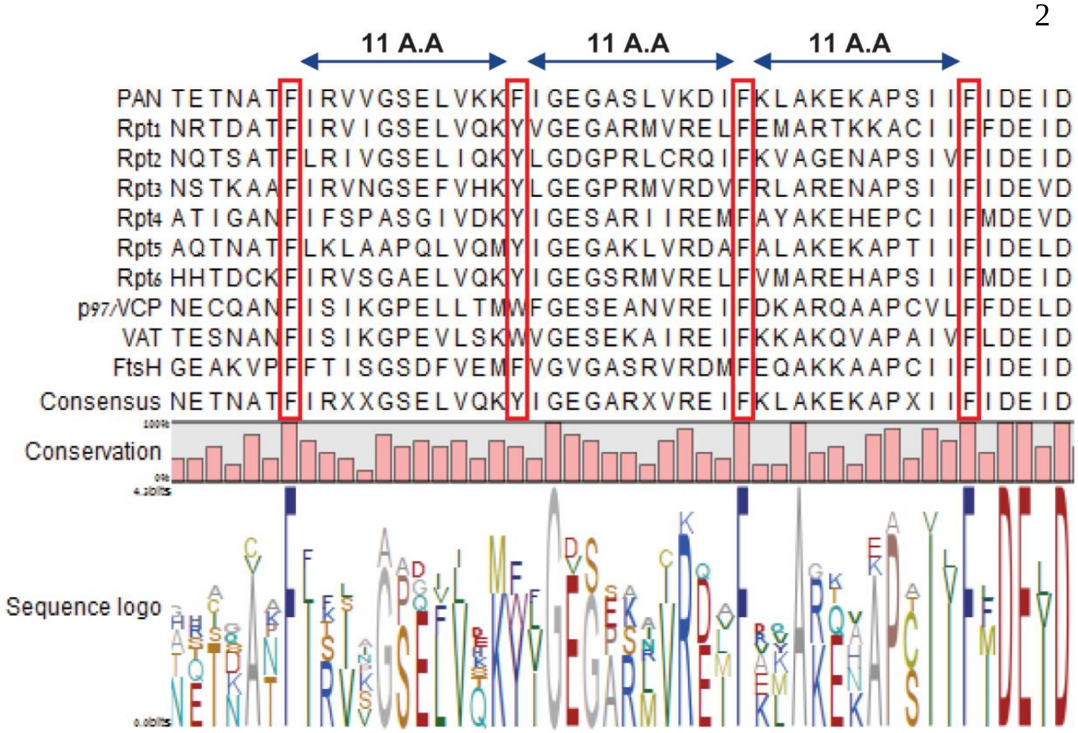

Figure S2: **Sequence alignment of AAA ATPase proteins, from bacteria to mammalian, underlining the conserved phenylalanine signature, related to Fig. 1.** Four consecutive phenylalanine residues, separated by 11 residues, define a unique motif (marked in red), illustrated on representative AAA ATPase complexes: PAN (*Methanocaldococcus jannaschii),* the six proteasomal Rpts 1-6 *(Saccharomyces cerevisiae),* p97/VCP *(Homo sapiens),* VAT *(Escherichia coli)* and FtsH *(Escherichia coli).* The conservation level of each amino acid is shown by graph bar, colored in pink and scaled from 0-100%, as well as sequence logo.

Fig. S3.

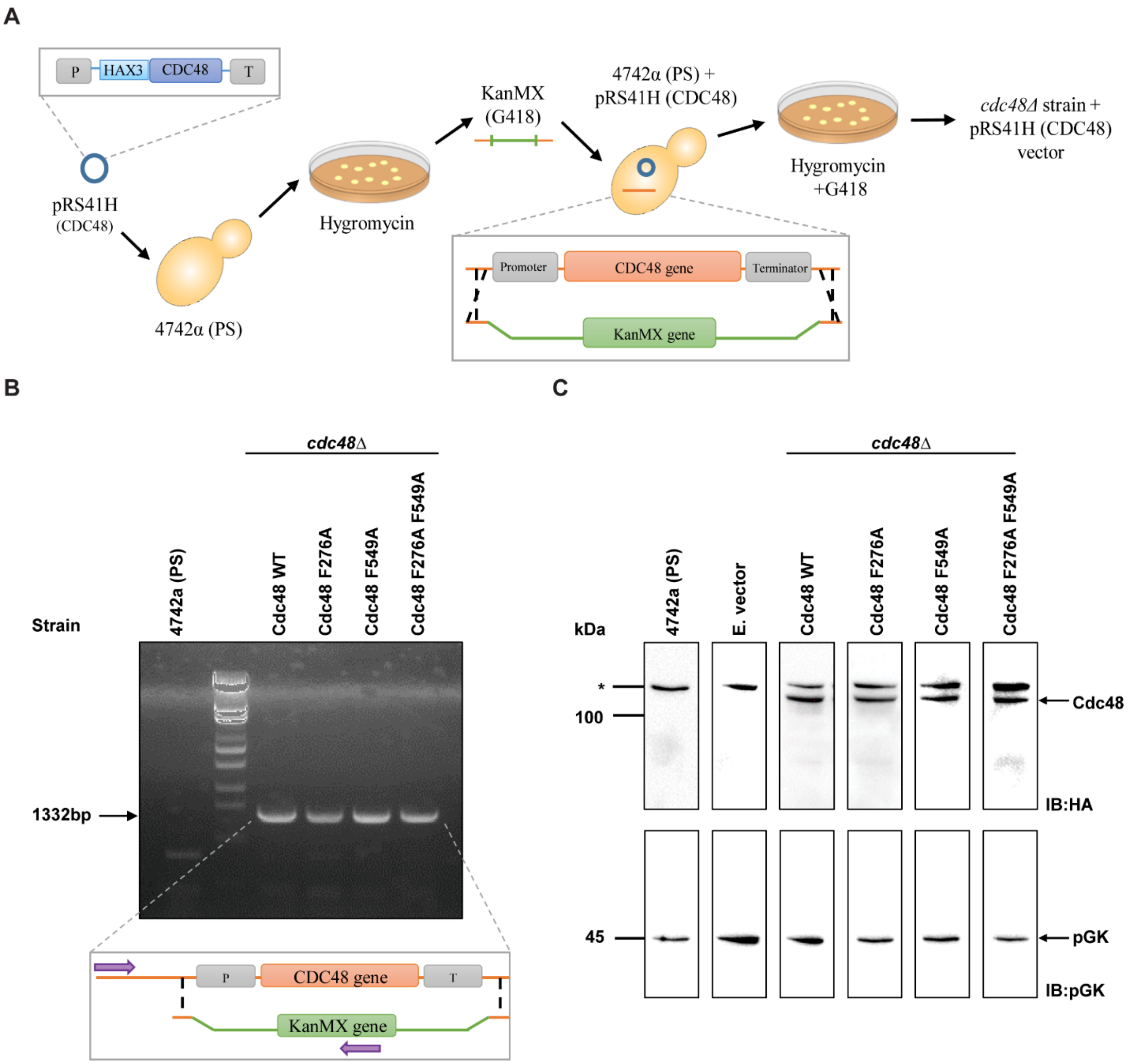

D

	K (1/hour)		
	25°C	30°C	37°C
BY4742a	0.66 ± 0.01	0.76 ± 0.02	0.89 ± 0.03
cdc48Δ *Cdc48 WT*	0.59 ± 0.01	0.72 ± 0.02	0.87 ± 0.06
cdc48Δ *Cdc48 F276A*	0.55 ± 0.01	0.73 ± 0.02	0.88 ± 0.02
cdc48Δ *Cdc48 F549A*	0.20 ± 0.01	0.32 ± 0.02	0.80 ± 0.03
cdc48Δ *Cdc48 F276A F549A*	0.52 ± 0.01	0.84 ± 0.03	0.90 ± 0.02

<u>Figure S3:</u> Yeast model design, related to Fig. 1F. (A) Cloning of *cdc48Δ* phenylalanine mutant strains. An illustration of the steps involved in the preparation of the indicated strains. The parental strain (PS) 4742 was transfected with pRS41H plasmid containing CDC48 gene (blue) tagged with HAx3 (light blue) in its N terminal tail (wild type or mutant: F276A, F549A F276A F549A), or with an empty vector (pRS41H plasmid without any gene), which provides Hygromycin resistance; (P) the endogenous promotor of CDC48 (gray); (T) the endogenous terminator of CDC48 (gray). Next, Hygromycin resistant yeast colonies were transfected with a linear fragment of the KanMX cassette that harbors homologous termini to the *CDC48* promoter and terminator, homologous recombination occurred, and they were seeded on Hygromycin and G418 plates. Hygromycin and G418 resistant colonies are presumably the desired strains of *cdc48Δ* + pRS41H (CDC48). **(B)** KanMX gene insertion validation. Colony PCR with yeast genome and KanMX primers (purple), see illustration below, representing the insertion of KanMX gene (1332bp) instead of CDC48 gene in the yeast genome. CDC48 gene colored in orange, KanMX gene colored in green, the promotor and terminator of CDC48 gene are colored in gray and marked as P and T, respectively. From left to right: the PS 4742α, *cdc48Δ* strains with pRS41H (CDC48) vector: wild type, F276A (D1), F549A (D2) and F276A F549A (D1&D2). **(C)** Western blot analysis of Cdc48 expression in the different *cdc48Δ* strains. From left to right: the PS 4742α, the PS 4742α with an empty vector, *cdc48Δ* strains with pRS41H (CDC48) vector: wild type, F276A (D1), F549A (D2) and F276A F549A (D1&D2). The detection of CDC48 gene expression by pRS41H plasmid was performed by immunoblotting against HA. Cellular levels of pGK served as loading controls. The bands marked as (*) are nonspecific targets of the HA antibody appearing in all samples. **(D)** Summary table of the growth rate values, calculated from the logistic curve in Fig. 1F, for all yeast strains mentioned above. While D1 (p97 F276A) and D1&D2 (p97 F276A F549A) display similar growth rates to the wild type and the PS 4742α, D2 (p97 F549A) mutant shows significantly ($p<0.0001$) slower growth rate at low temperatures, while @ 37°C all strains grow in similar rates.

Fig. S4.

A

A	analyte
B	ligand
AB	complex
AB▪	conformational change
tc	flow rate-independent component of the mass transfer constant
R_{max}	analyte binding capacity of the surface (RU)
f	flow rate (μl/min)
R_I	bulk refractive index contribution in the sample
$K(on)_1$	association rate constant for analyte binding (1/M•sec)
$K(off)_1$	dissociation rate constant for ligand-analyte complex (1/sec)
$K(on)_2$	association rate constant for the stabilizing change (1/sec)
$K(off)_2$	reverse rate constant for the stabilizing change (1/sec)
Concentration	analyte concentration (M)

B

(a) $A + B \rightleftharpoons AB$

(b) $AB \rightleftharpoons AB^{\blacksquare}$

(c) $AB + AB^{\blacksquare} + R_I$

C

(d) $A_{(solution)} = Concentration$

$$A_{[0]} = 0$$

$$\frac{dA}{dt} = tc * f^{\frac{1}{3}} * (Concentration - A) - (K(on)_1 * A * B - K(off)_1 * AB)$$

(e) $B_{[0]} = R_{max}$

$$\frac{dB}{dt} = -(K(on)_1 * A * B - K(off)_1 * AB)$$

(f) $AB_{[0]} = 0$

$$\frac{dAB}{dt} = (K(on)_1 * A * B - K(off)_1 * AB) - (K(on)_2 * AB - K(off)_2 * AB^{\blacksquare})$$

(g) $AB^{\blacksquare}{}_{[0]} = 0$

$$\frac{dAB^{\blacksquare}}{dt} = (K(on)_2 * AB - K(off)_2 * AB^{\blacksquare})$$

D

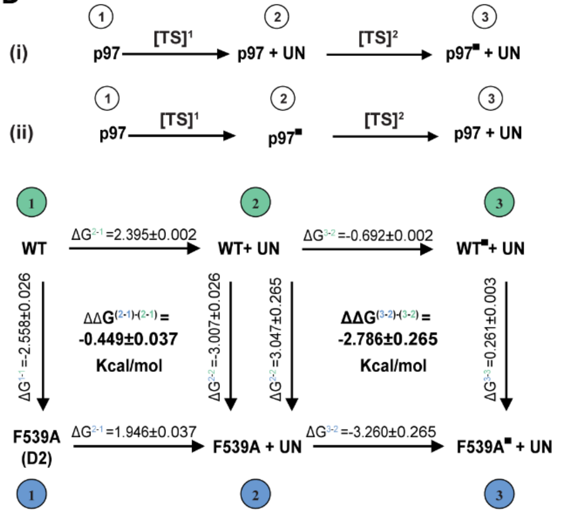

E

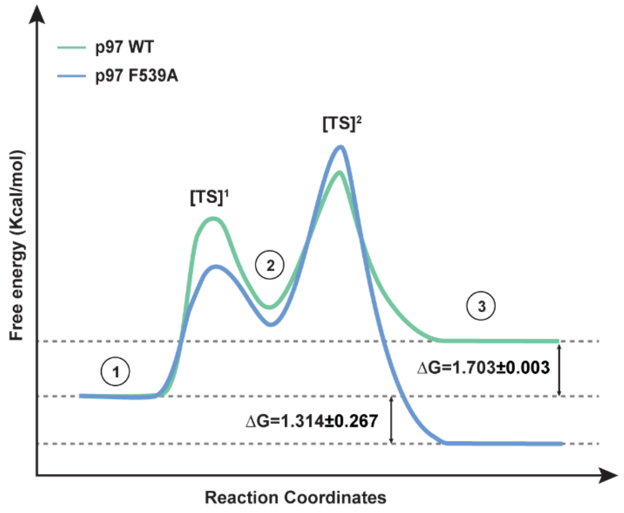

Figure S4: Two state reaction rate equations, related to Fig. 2D. (A) Parameters description. **(B)** The reaction equations of Analyte which binds to ligand (a). Complex AB transforms to AB■ which cannot dissociate directly to A+B (b). The total response in SPR measurements is described in equation (c). **(C)** The rate equations of the reactants and products (d-g). **(D)** Top: two possible scenarios for p97-UN complex association: (i) p97 binds UN, followed by a conformational transition in p97. (ii) Structural transition occurs within p97, in response to UN binding. Bottom: double mutant cycle analysis calculated for scenario (i) from the association and dissociation constants deduced from SPR analysis (Figure 2D). Both cycles demonstrate the high coupling effect of F539A mutation on UN binding**. (E)** Gibbs free energy diagram illustrating the two-state model of p97 wild type (green) and p97 F539A (blue). p97 F539A-UN complex formation is a highly favorable reaction, in line with the high affinity observed in Figure 2A.

Fig. S5.

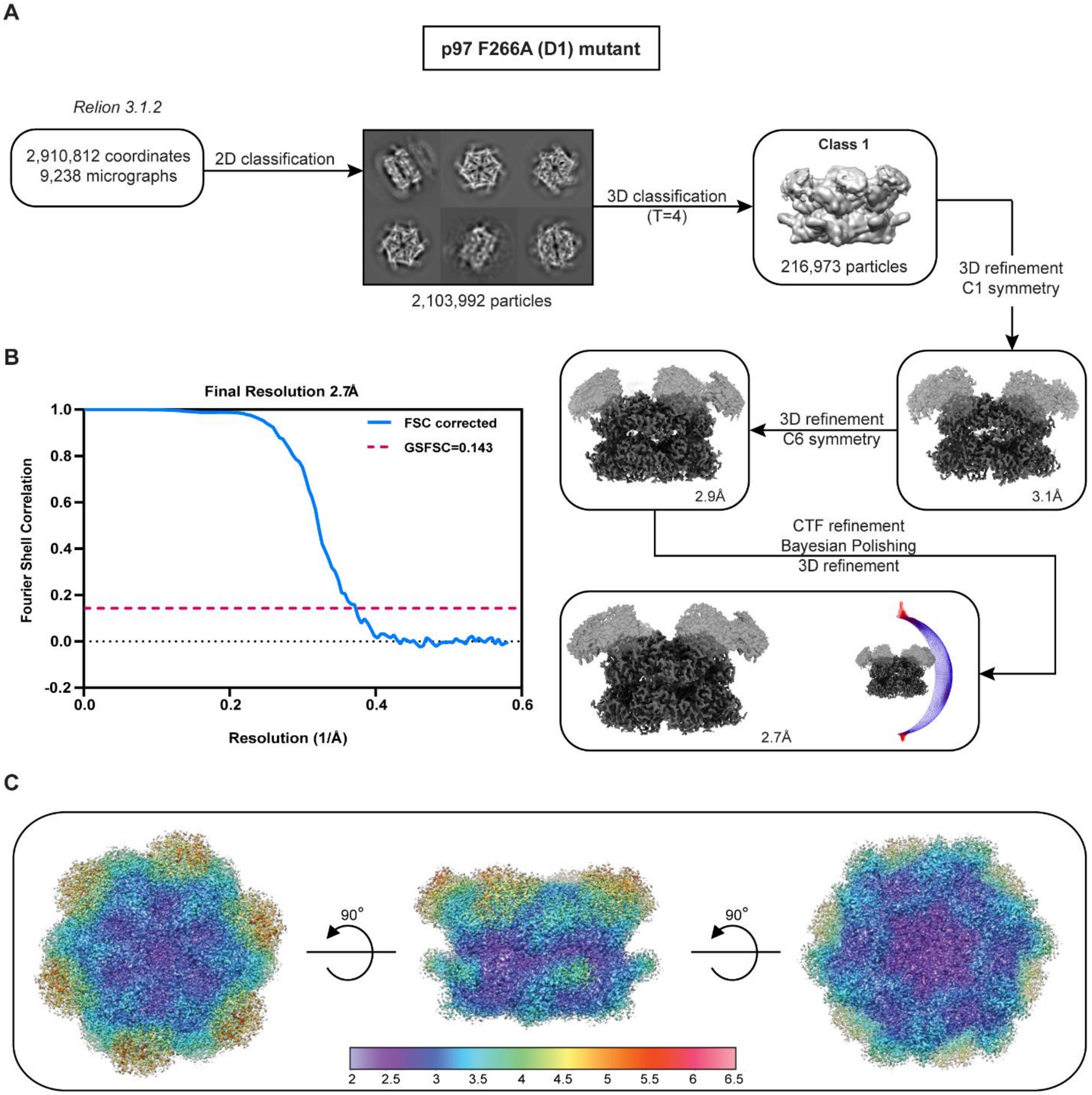

<u>Figure S5:</u> Cryo-EM data processing of p97 F266A (D1), related to Fig. 3A. (A) Data processing workflow analyzed by RELION 3.1.2. Processing flow chart of p97 F266A cryo-EM data, including particle selection, 2D and 3D classifications, particle sorting, masking and final map reconstruction. The resolved structure and its Euler angles distribution are shown side-by-side. In all maps the NTDs are presented in light gray (unsharpened map), while the D1 and D2 domains are shown in dark gray (sharpened map). The maps sigma values are as follows (unsharpened: NTDs; sharpened: D1-D2 domains): C1 symmetry (σ=0.0033; σ=0.0273), C6 symmetry (σ=0.0022; σ=0.0203) and the final reconstruction (σ=0.0029; σ=0.0289). **(B)** FSC curve. FSC curve of the corrected map (blue) is shown following RELION post-processing. The resolution was determined by the FSC=0.143 criterion (pink). **(C)** Local resolution map. Local resolution of the reconstruction map from top, side and bottom views. The map was calculated using RELION. Resolution scale bar is presented below the different views scaling from 2-6.5Å (purple to pink).

Fig. S6.

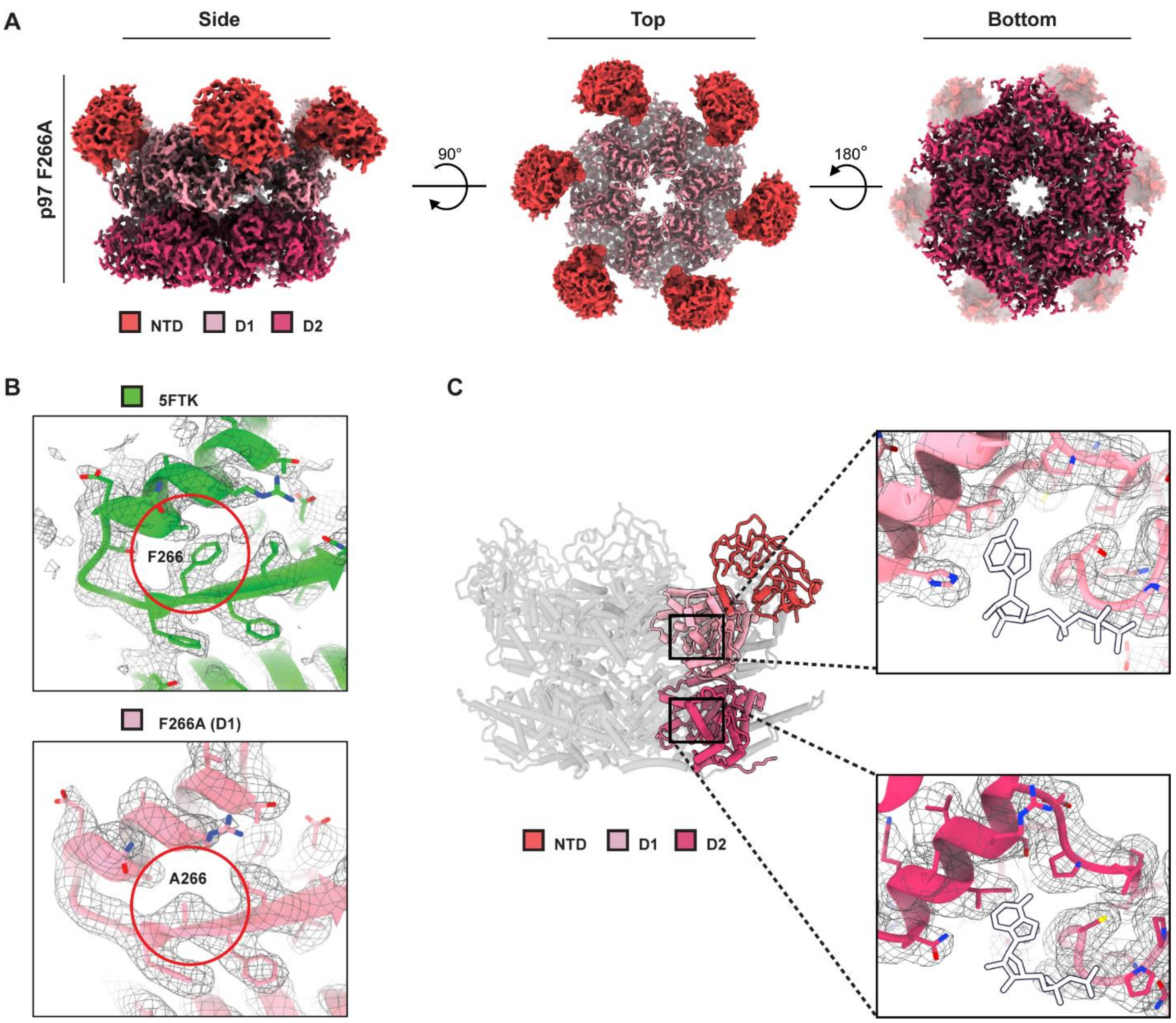

<u>Figure S6:</u> p97 F266A (D1) map characterization, related to Fig. 3A. (A) Cryo-EM map of p97 F266A at different views. The three main domains are marked by (NTD) light coral, (D1), light pink and (D2) pink. The NTDs (unsharpened map) are shown at σ=0.0029, while D1 and D2 domains (sharpened map) are presented at σ=0.0289. **(B)** Replacement of phenylalanine to alanine is confirmed by the cryo-EM map. The volume map of wild type p97 (EMD-3296, PDB:5FTK, green) at position 266 shows a density corresponding to phenylalanine residue, in contrast to p97 F266A (D1, pink), in which the observed density at this location corresponds to alanine side chain. **(C)** Nucleotide binding pocket within the p97 F266A structure. p97 F266A (D1) coordinates model focusing on the highlighted monomers nucleotide binding pockets: D1 (light pink) and D2 (pink), magnified and fitted into the EM map. The theoretical positions of the nucleotides are presented as ADP/ATP molecules (white). While no volume map is observed at D1 ring, D2 ring shows a partial density corresponding to the base and the phosphorus groups.

Fig. S7.

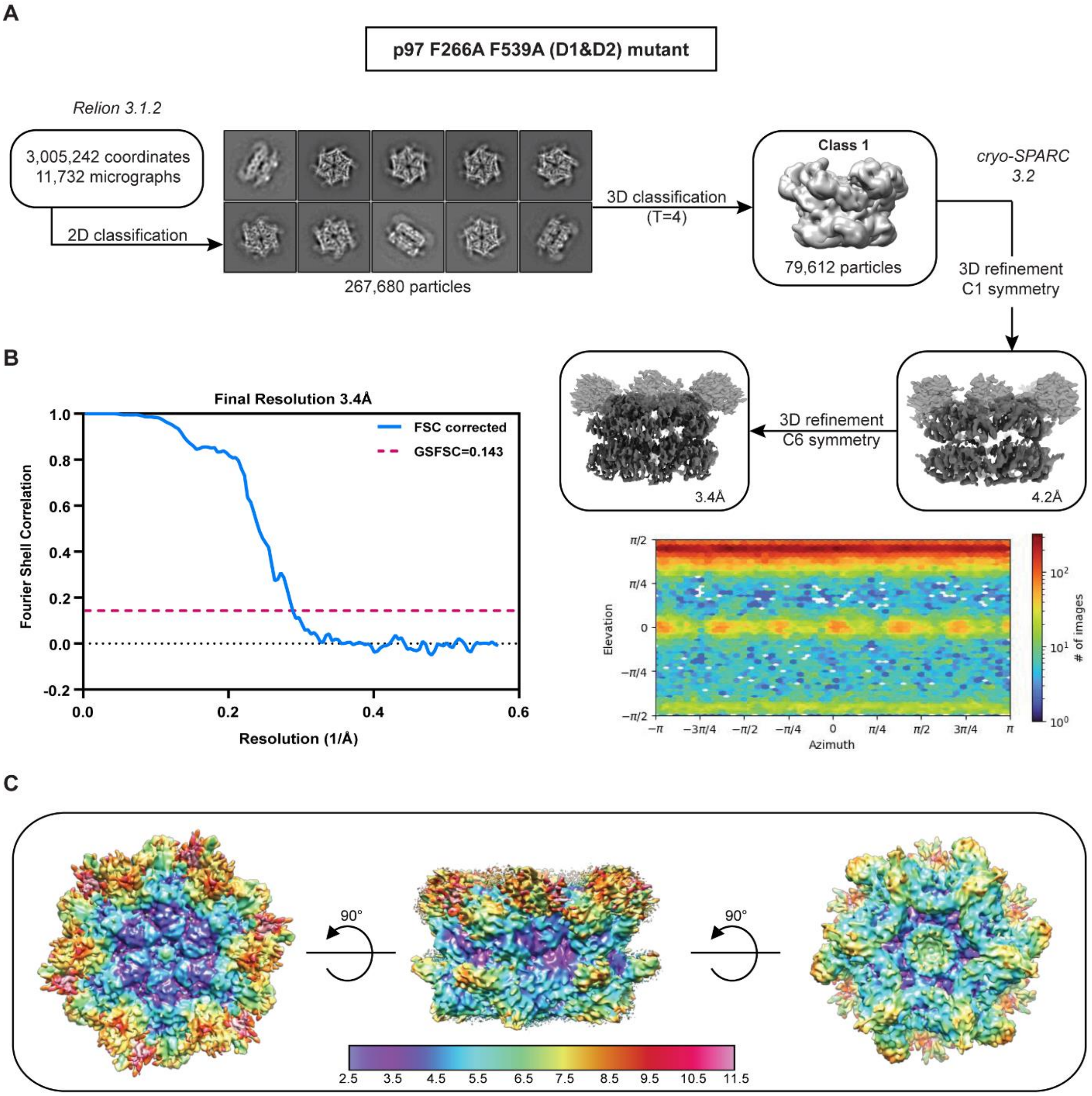

<u>Figure S7:</u> Cryo-EM data processing of p97 F266A F539A (D1&D2), related to Fig. 3B. (A) Data processing workflow analyzed by RELION 3.1.2 and cryoSPARC 3.2. Processing flow chart of p97 F266A F539A cryo-EM data, including particle selection, 2D and 3D classifications, particle sorting, masking and final map reconstruction. In all maps the NTDs are presented in light gray (unsharpened map), while the D1 and D2 domains are shown in dark gray (sharpened map). The maps sigma values are as follows (unsharpened: NTDs; sharpened: D1-D2 domains): C1 symmetry (σ=0.0642; σ=0.2440) and the final reconstruction (σ=0.0631; σ=0.3520). The distribution of Euler angles is presented below the resolved structure. **(B)** FSC curve. FSC curve of the corrected map (blue) is shown following cryoSPARC homogeneous refinement and post-processing. The resolution was determined by the FSC=0.143 criterion (pink). **(C)** Local resolution map. Local resolution of the reconstruction map from top, side and bottom views. The map was calculated using cryo-SPARC. Resolution scale bar is presented below the different views scaling range: 2.5-11.5Å (purple to pink).

Fig. S8.

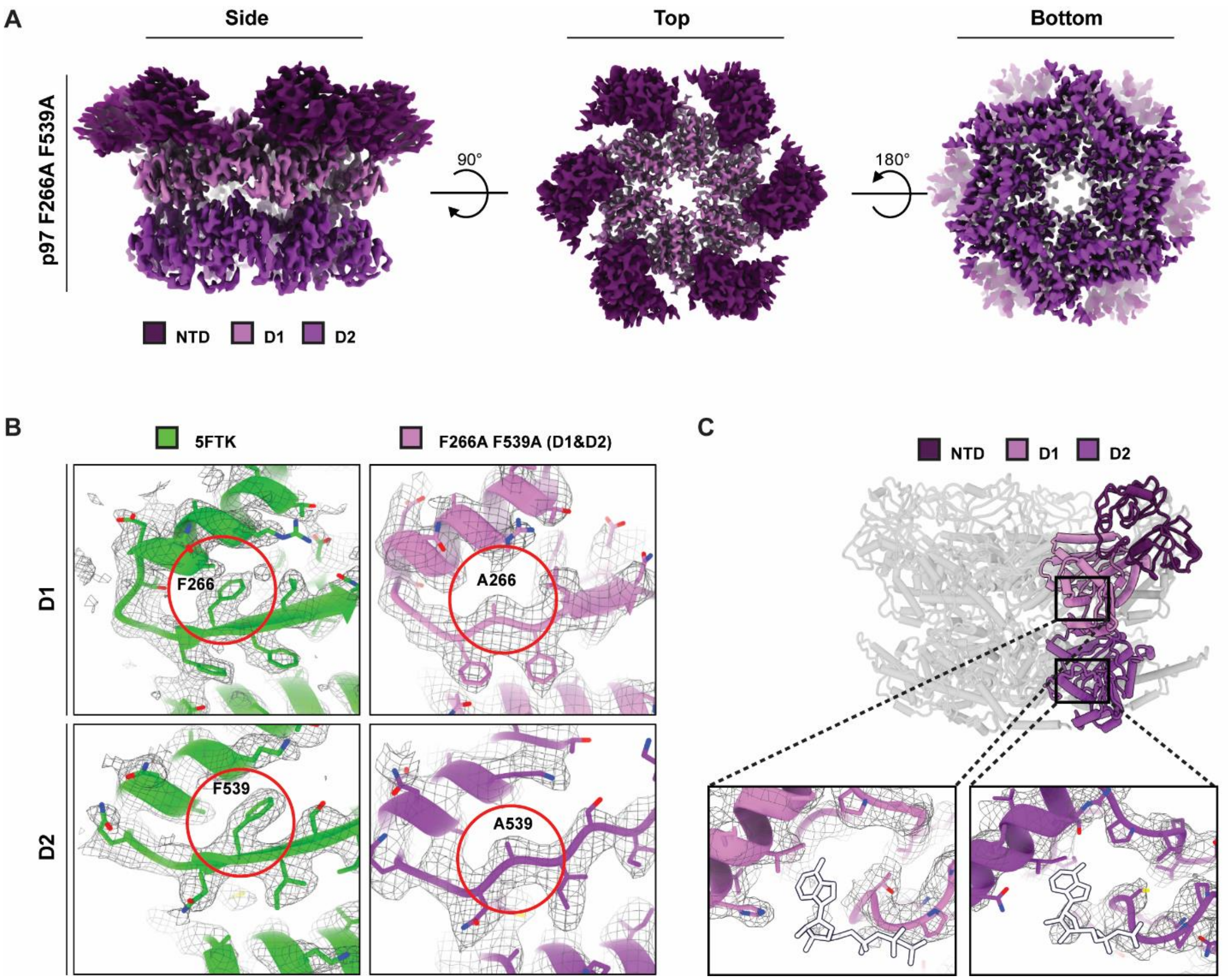

Figure S8: p97 F266A F539A (D1&D2) map characterization, related to Fig. 3B. (A) Cryo-EM sharpened map of p97 F266A F539A. The three main domains are marked: (NTD) purple, (D1) plum and (D2) orchid. The NTDs (unsharpened map) are shown at σ=0.0631, while D1 and D2 domains (sharpened map) are presented at σ=0.3520. **(B)** Replacement of phenylalanine to alanine is confirmed by the EM map. The volume map of wild type p97 (EMD-3296, PDB ID:5FTK, green) at positions 266 and 539 show densities corresponding to phenylalanine resides, in contrast to p97 F266A F539A (purple), in which the observed densities at these positions correspond to alanine side chain. **(C)** Nucleotide binding pocket within the p97 F266A F539A structure. p97 F266A F539A (D1&D2) coordinates model focusing on the highlighted monomers D1 (plum) and D2 (orchid) rings at nucleotides positions, magnified and fitted into the EM map. The expected positions of the nucleotides are presented as ADP/ATP molecules (white). Partial densities corresponding to the sugar and the phosphorus groups are observed in both domains.

Fig. S9.

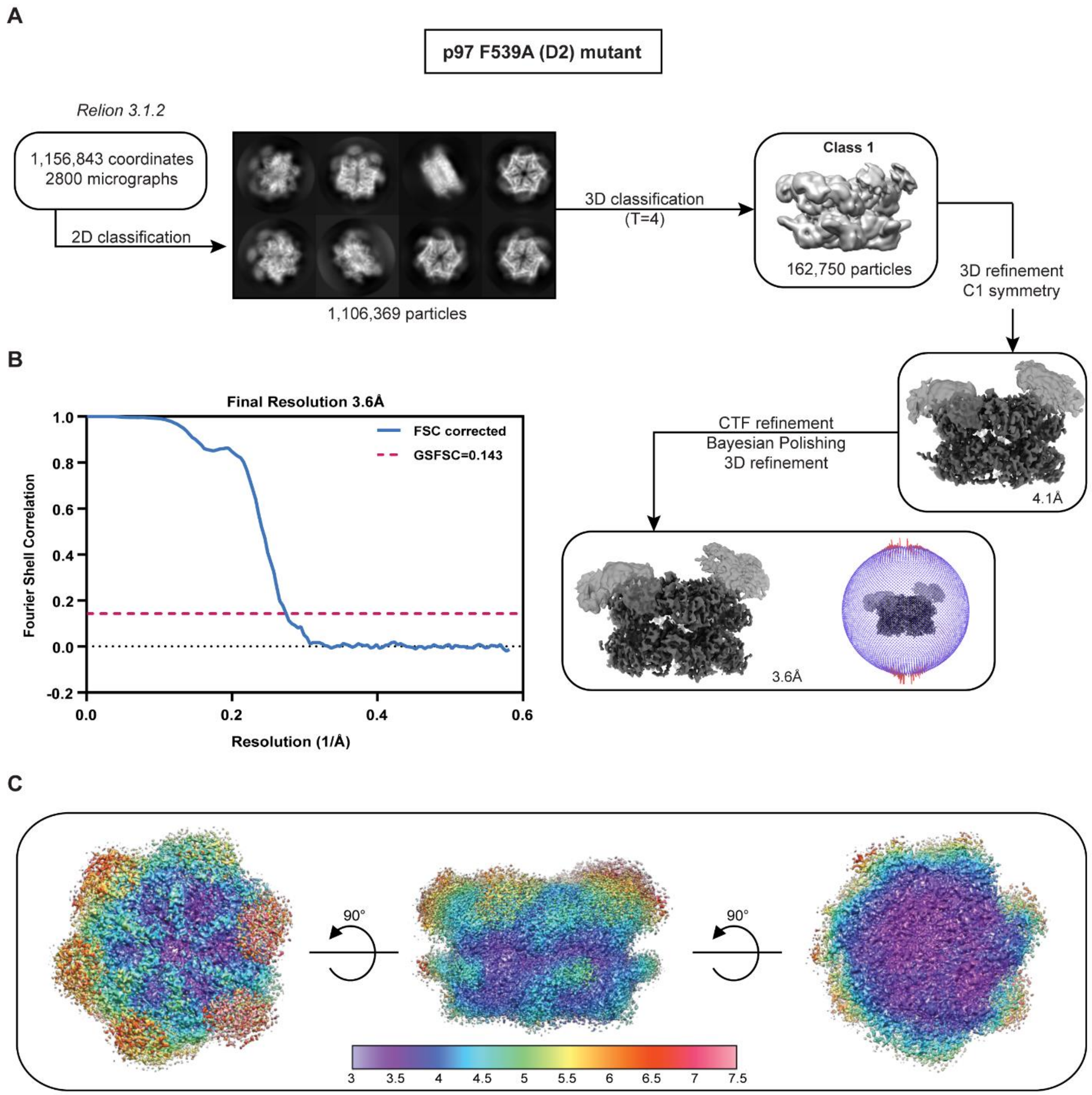

Figure S9: Cryo-EM data processing of p97 F539A (D2), related to Fig. 4A. (A) Data processing workflow analyzed by RELION 3.1.2. Processing flow chart of p97 F539A cryo-EM data, including particle selection, 2D and 3D classifications, particle sorting, masking and final map reconstruction. The resolved structure and its Euler angles distribution are shown side-by-side. In all maps the NTDs are presented in light gray (unsharpened map), while the D1 and D2 domains are shown in dark gray (sharpened map). The maps' sigma values are as follows (unsharpened: NTDs; sharpened: D1-D2 domains): C1 symmetry (σ=0.0040; σ=0.0107) and the final reconstruction (σ=0.0046; σ=0.0207). **(B)** FSC curve. FSC curve of the corrected map (blue) is shown following RELION post-processing. The resolution was determined by the FSC=0.143 criterion (pink). **(C)** Local resolution map. Local resolution of the reconstruction map from top, side and bottom views. The map was calculated using RELION. Resolution scale bar is presented below the different views scaling range: 3-7.5Å (purple to pink).

Fig. S10.

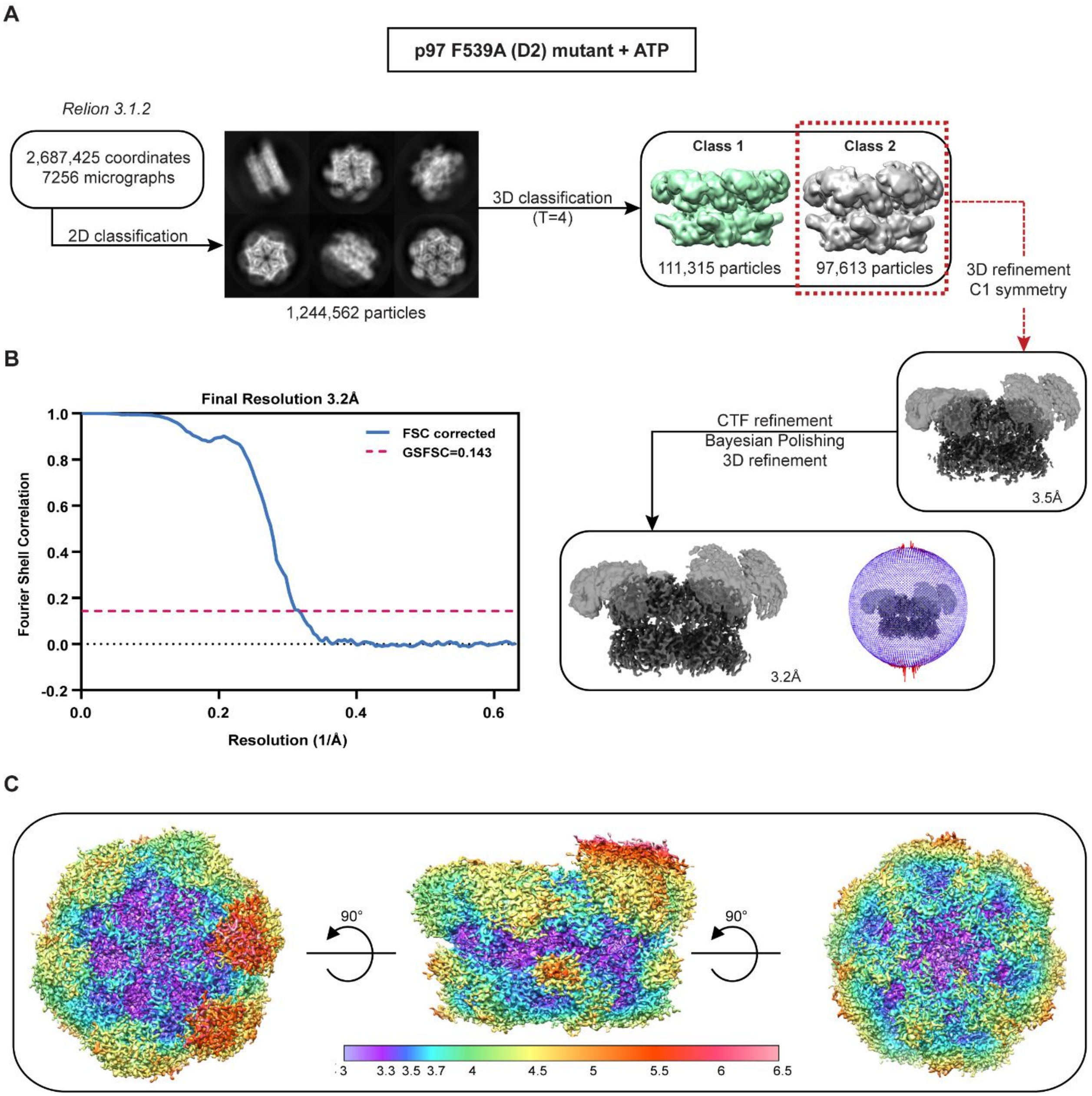

<u>Figure S10:</u> Cryo-EM data processing of p97 F539A (D2) in the presence of ATP, related to Fig. 4. (A) Data processing workflow analyzed by RELION 3.1.2. Processing flow chart of p97 F539A (in the presence of ATP) cryo-EM data, including particle selection, 2D and 3D classifications, particle sorting, masking and final map reconstruction. The resolved structure and its Euler angles distribution are shown side-by-side. In all maps the NTDs are presented in light gray (unsharpened map), while the D1 and D2 domains are shown in dark gray (sharpened map). The maps sigma values are as follows (unsharpened: NTDs; sharpened: D1-D2 domains): C1 symmetry (σ=0.00262; σ=0.0211) and the final reconstruction (σ=0.0027; σ=0.0295). **(B)** FSC curve. FSC curve of the corrected map (blue) is shown after RELION post-processing. The resolution was determined by the FSC=0.143 criterion (pink). **(C)** Local resolution map. Local resolution of the reconstruction map from top, side and bottom views. The map was calculated using RELION. Resolution scale bar is presented below the different views scaling range: 3-6.5Å (purple to pink).

Fig. S11.

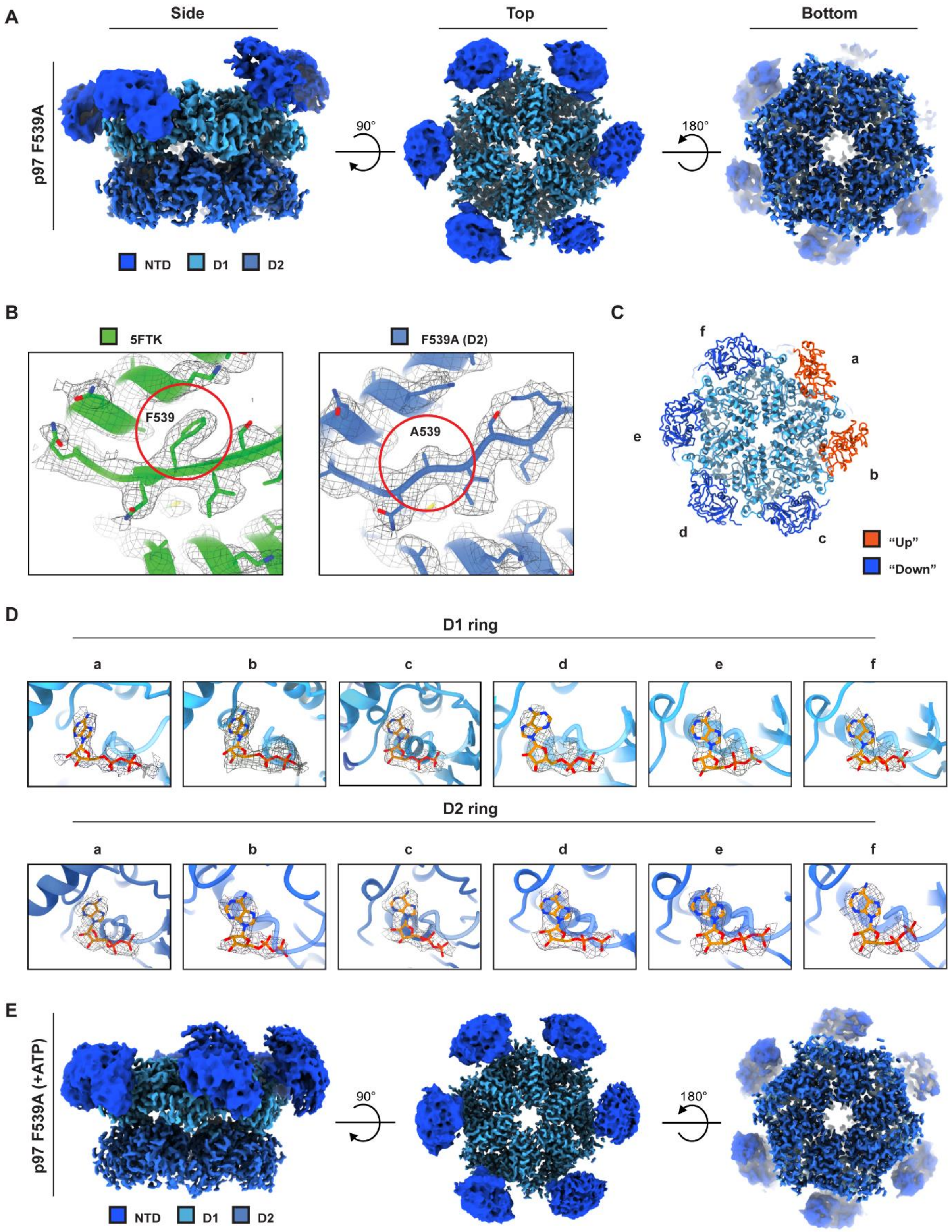
A
Side
Top
Bottom
p97 F539A
90°
180°
NTD
D1
D2
B
5FTK
F539A (D2)
F539
A539
C
a
b
c
d
e
f
"Up"
"Down"
D
D1 ring
a
b
c
d
e
f
D2 ring
a
b
c
d
e
f
E
p97 F539A (+ATP)
90°
180°
NTD
D1
D2

Figure S11: p97 F539A (D2) map characterization, related to Fig. 4. (A) Cryo-EM sharpened map of p97 F539A. The three main domains are marked: (NTD) royal blue, (D1) sky blue and (D2) cornflower blue. The NTDs (unsharpened map) are shown at σ=0.0046, while D1 and D2 domains (sharpened map) are presented at σ=0.0207. **(B)** Replacement of phenylalanine to alanine is confirmed by the EM map. The volume map of wild type p97 (EMD-3296, PDB ID:5FTK, green) at position 539 shows a density corresponding to a phenylalanine reside, in contrast to p97 F539A (blue) in which the observed density at this location corresponds to alanine side chain. **(C)** Top view of p97 F539A NTDs and D1 ring. Protomers with NTDs in the "up" state (a-b) are colored orange, while protomers with NTDs in the "down" state (c-f) are colored blue. **(D)** The nucleotide pockets of each protomer, as depicted in panel C, in the D1 and D2 rings. **(E)** Cryo-EM sharpened map of p97 F539A in the presence of ATP. The three main domains are marked by (NTD) royal blue, (D1) sky blue and (D2) cornflower blue. The NTDs (unsharpened map) are shown at σ=0.00406, while D1 and D2 domains (sharpened map) are presented at σ=0.0245.

Fig. S12.

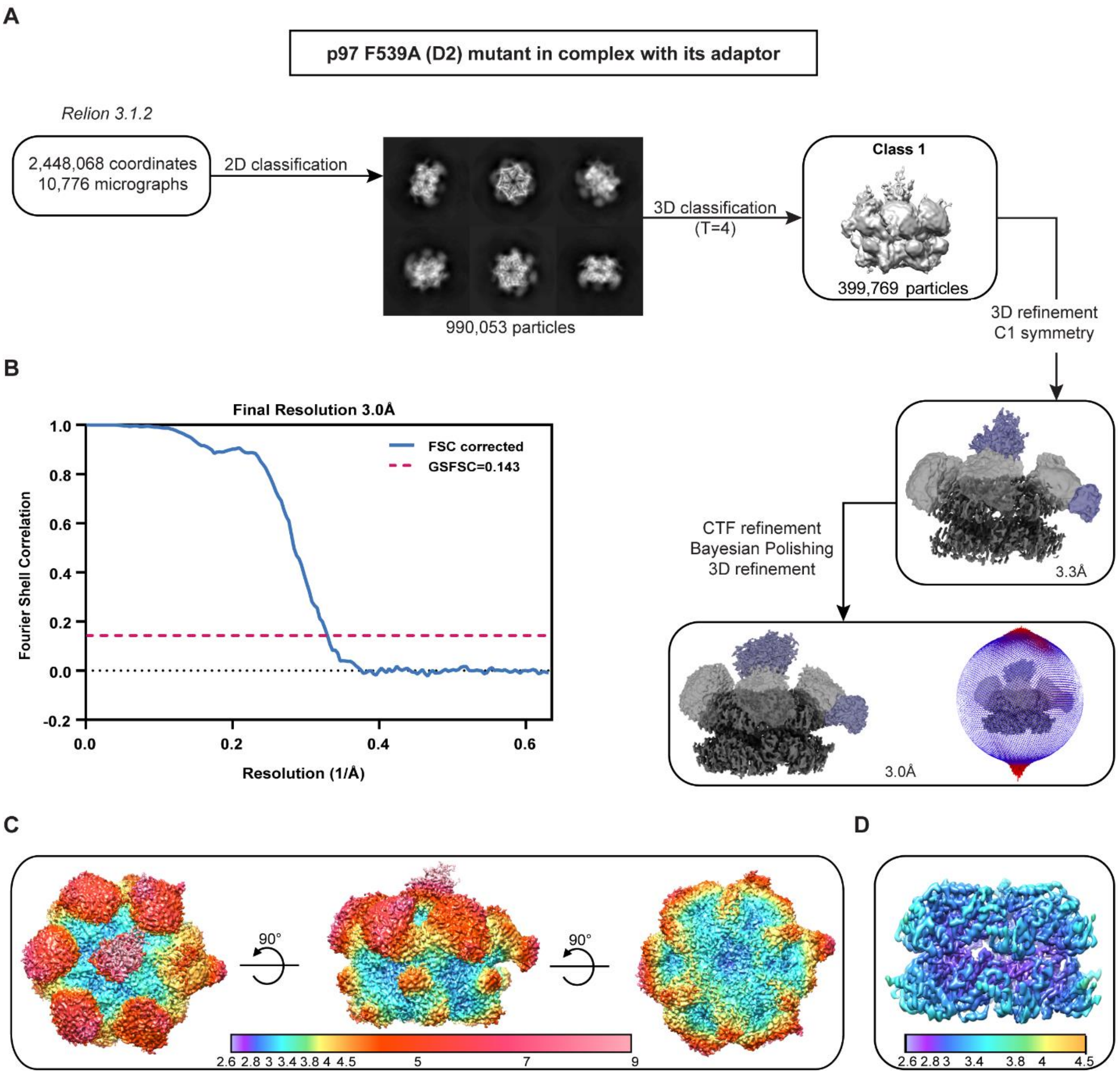

__Figure S12:__ **Cryo-EM data processing of p97 D2 (F539A) mutant-adaptor complex, related to Fig. 5. (A)** Data processing workflow analyzed by RELION 3.1.2. Processing flow chart of p97 F539A-adaptor complex cryo-EM data, including particle selection, 2D and 3D classifications, particle sorting, masking and final maps reconstruction. Each resolved structure and its Euler angles distribution are shown side-by-side. In all maps the NTDs are presented in light gray (unsharpened map), while the D1 and D2 domains are shown in dark gray (sharpened map). Npl4 and its UBXL domain are marked in purple (unsharpened map). The maps sigma values are as follows (unsharpened: NTDs/ Npl4; sharpened: D1-D2 domains): C1 symmetry (σ=0.0019/ σ=0.00053; σ=0.0173) and the final reconstruction (σ=0.0019/ σ=0.0053; σ=0.0141). **(B)** FSC curves. FSC curve of the corrected map (blue) is shown following RELION post-processing. The resolution was determined by the FSC=0.143 criterion (pink). **(C)** Local resolution map. Local resolution of the reconstruction map from top, side and bottom views. The map was calculated using RELION. Resolution scale bar is presented below the different views scaling from 2.6-9.0Å (purple to pink). **(D)** Local resolution map of the reconstruction map focusing on the D1/D2 domains. Resolution scale bar is presented below scaling from 2.6-4.5Å (purple to yellow).

1 **Fig. S13.**

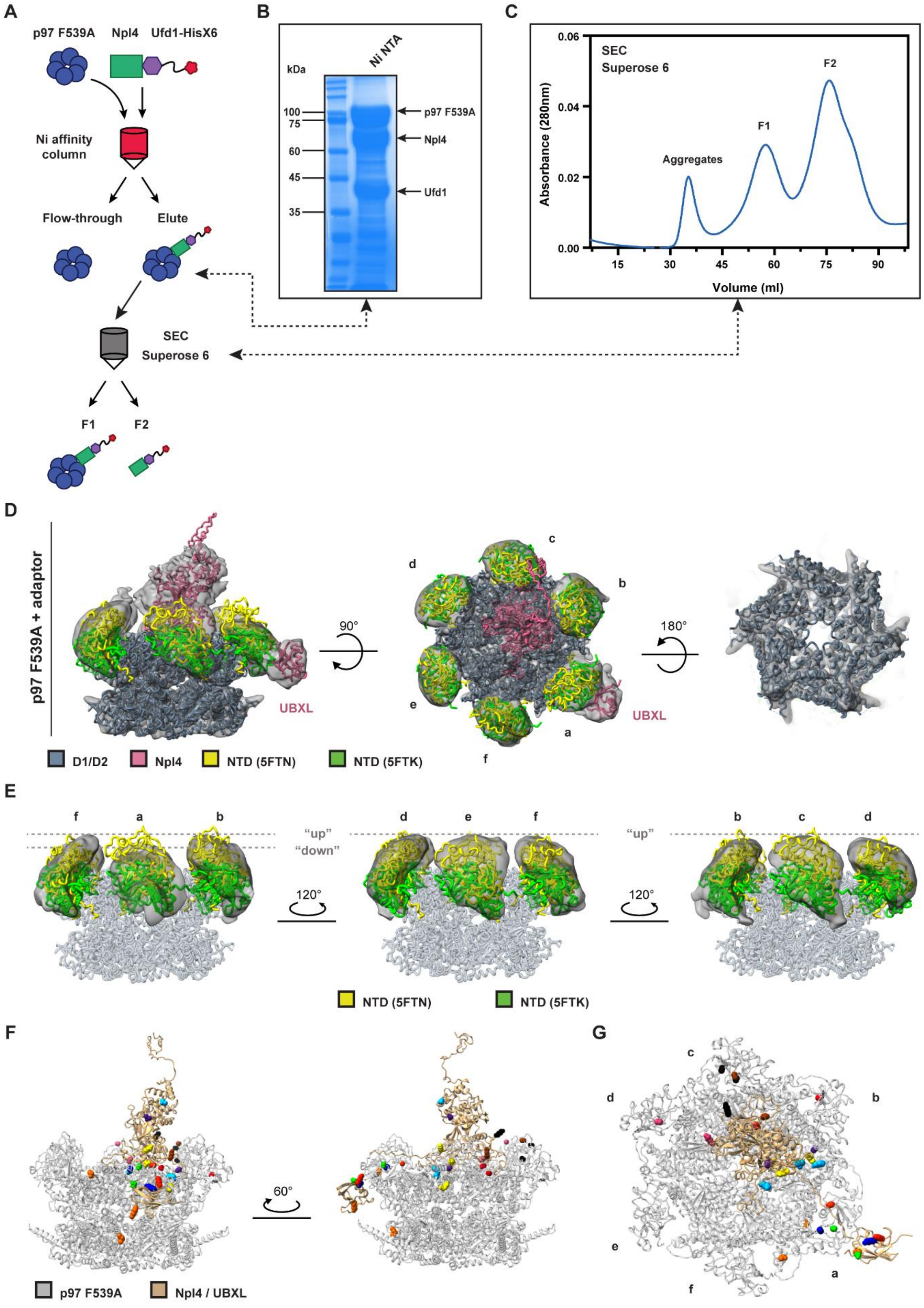

A
p97 F539A
Npl4
Ufd1-HisX6
Ni affinity column
Flow-through
Elute
SEC
Superose 6
F1
F2
B
Ni NTA
kDa
100
75
60
45
35
p97 F539A
Npl4
Ufd1
C
SEC
Superose 6
Absorbance (280nm)
Volume (ml)
Aggregates
F1
F2
0.06
0.04
0.02
0.00
15
30
45
60
75
90
D
p97 F539A + adaptor
90°
180°
UBXL
UBXL
a
b
c
d
e
f
D1/D2
Npl4
NTD (5FTN)
NTD (5FTK)
E
f
a
b
d
e
f
b
c
d
"up"
"down"
"up"
120°
120°
NTD (5FTN)
NTD (5FTK)
F
60°
p97 F539A
Npl4 / UBXL
G
a
b
c
d
e
f

<u>Figure S13:</u> p97 F539A-adaptor complex map characterization, related to Fig. 5. (A) p97 F539A-UN complex purification workflow. **(B)** Coomassie stained SDS-PAGE following Ni affinity column (Ni NTA) presenting the complex p97 F539A-UN (Ufd1/Npl4). **(C)** Size exclusion chromatography (SEC: Superose 6) presenting the fractionation pattern: aggregates, p97 F539A-UN complex (F1) and UN (F2). **(D)** Cryo-EM map of p97 F539A-adaptor complex is presented following gaussian filter from side, top and bottom views (light gray). PDBs of wild type p97 "up" (PDB: 5FTN, yellow) and "down" (PDB: 5FTK, green) states are aligned to the map, as well as the C terminal and UBXL domains of Npl4 (PDB: 6JWJ and 2PJH, pink). **(E)** p97 F539A NTDs conformations upon UN binding. Gaussian filtered map of p97 NTDs from p97 F539A-adaptor complex map, which are aligned with the wild type p97 "up" (PDB: 5FTN, yellow) and "down" (PDB:5FTK, green) conformations. The six NTDs of p97 are presented from different angles demonstrating that protomers (b-f) are in the "up" state, while protomer (a) is in the "down" state. **(F)** and **(G)** **I**llustration of cross-linked p97 F539A-adaptor complex following mass spectrometry analysis. Pairs of residues involved in crosslinking between p97 F539A (gray) and Npl4 (tan) are marked in the matching color and are shown as spheres.

Fig. S14.

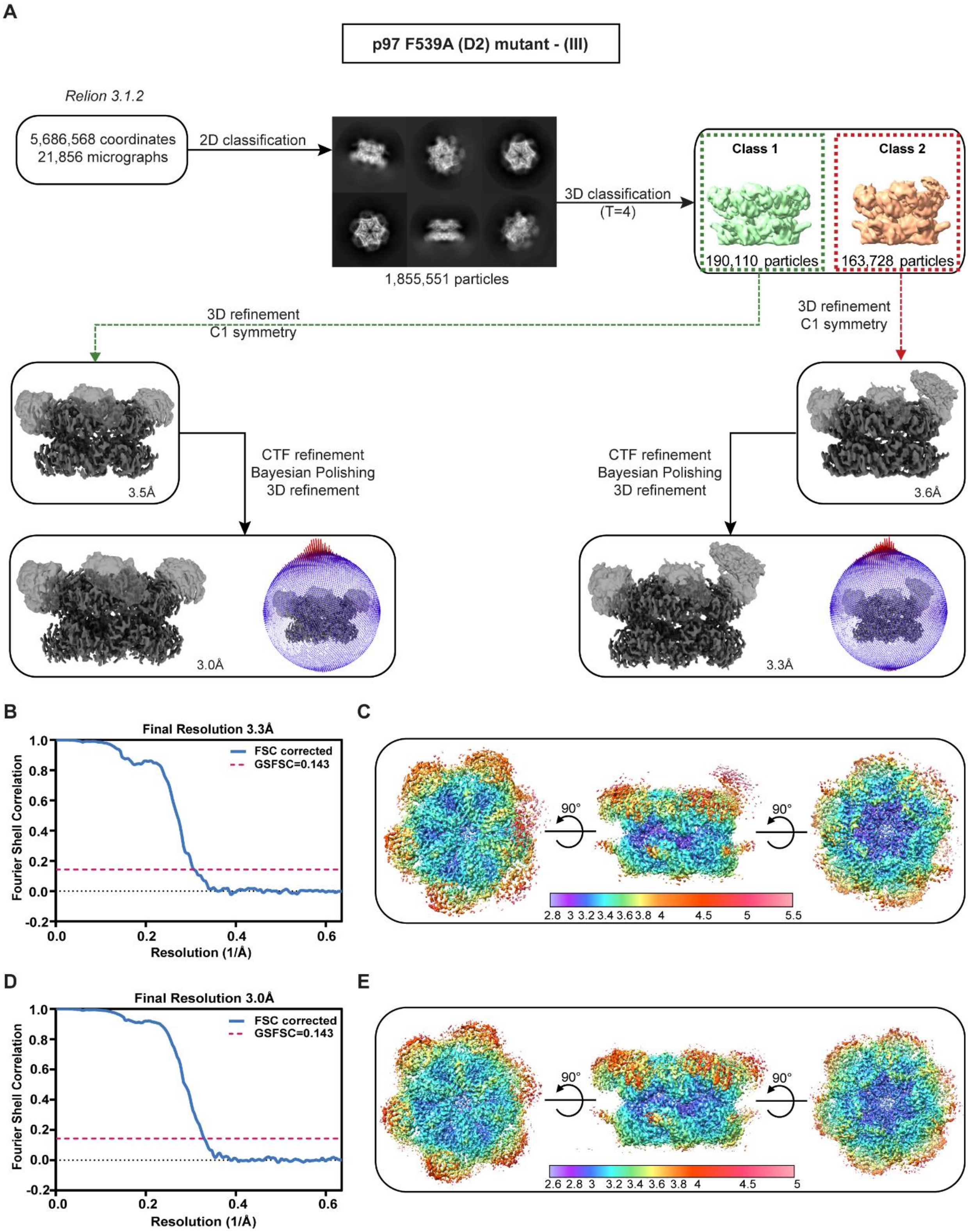
A
p97 F539A (D2) mutant - (III)
Relion 3.1.2
5,686,568 coordinates
21,856 micrographs
2D classification
1,855,551 particles
3D classification
(T=4)
Class 1
Class 2
190,110 particles
163,728 particles
3D refinement
C1 symmetry
3D refinement
C1 symmetry
3.5Å
3.6Å
CTF refinement
Bayesian Polishing
3D refinement
CTF refinement
Bayesian Polishing
3D refinement
3.0Å
3.3Å
B
Final Resolution 3.3Å
FSC corrected
GSFSC=0.143
Fourier Shell Correlation
Resolution (1/Å)
C
90°
90°
2.8 3 3.2 3.4 3.6 3.8 4 4.5 5 5.5
D
Final Resolution 3.0Å
FSC corrected
GSFSC=0.143
Fourier Shell Correlation
Resolution (1/Å)
E
90°
90°
2.6 2.8 3 3.2 3.4 3.6 3.8 4 4.5 5

Figure S14: Cryo-EM data processing of p97 D2 (F539A) mutant after UN release (short incubation), related to Fig. 6. (A) Data processing workflow analyzed by RELION 3.1.2. Processing flow chart of p97 F539A cryo-EM data following UN incubation, including particle selection, 2D and 3D classifications, particle sorting, masking and final maps reconstruction. The resolved structure and its Euler angles distribution are shown side-by-side. In all maps the NTDs are presented in light gray (unsharpened map), while the D1 and D2 domains are shown in dark gray (sharpened map). The maps sigma values are as follows (unsharpened: NTDs; sharpened: D1-D2 domains): For p97 F539A class 2: C1 symmetry (σ=0.0043; σ=0.0120) and the final reconstruction (σ=0.0043; σ=0.0220). For p97 F539A class 1 (ADP state): C1 symmetry (σ=0.0043; σ=0.0142) and the final reconstruction (σ=0.0043; σ=0.0234). **(B)** p97 F539A state III; FSC curves. FSC curve of the corrected map (blue) is shown after RELION post-processing. The resolution was determined by the FSC=0.143 criterion (pink). **(C)** p97 F539A state III; Local resolution map. Local resolution of the reconstruction map from top, side and bottom views. The maps were calculated using RELION. Resolution scale bar is presented below the different views scaling from 2.8-5.5Å (purple to pink). **(D)** p97 F539A ADP state; FSC curves. FSC curve of the corrected map (blue) is shown following RELION post-processing. The resolution was determined by the FSC=0.143 criterion (pink). **(E)** p97 F539A ADP state; Local resolution map. Local resolution of the reconstruction map from top, side and bottom views. The maps were calculated using RELION. Resolution scale bar of the is presented below the different views scaling from 2.6-5.0Å (purple to pink).

Fig. S15.

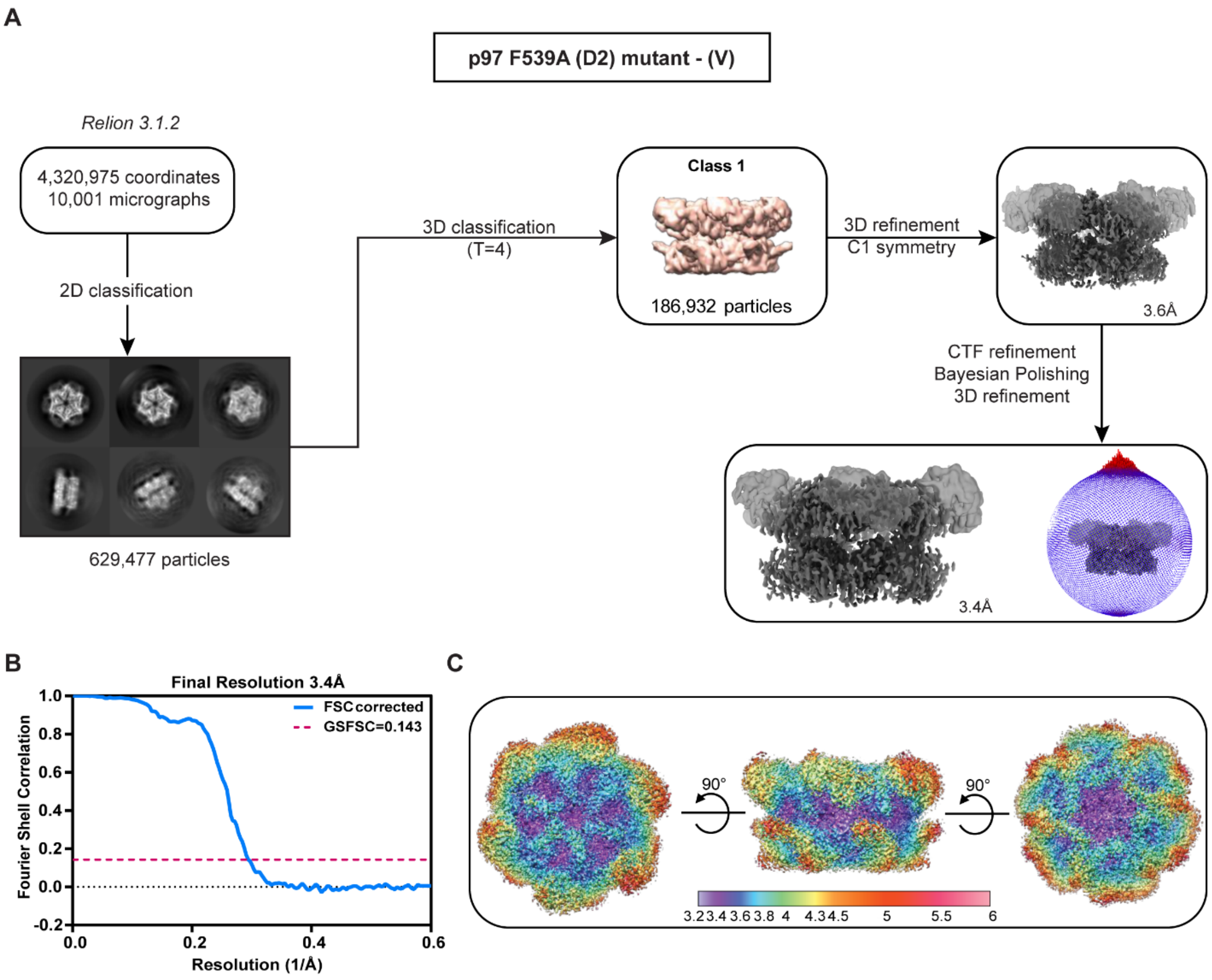

Figure S15: Cryo-EM data processing of p97 D2 (F539A) mutant upon UN release (extended incubation), related to Fig. 6. (A) Data processing workflow analyzed by RELION 3.1.2. Processing flow chart of p97 F539A cryo-EM data following UN incubation, including particle selection, 2D and 3D classifications, particle sorting, masking and final maps reconstruction. The resolved structure and its Euler angles distribution are shown side-by-side. In all maps the NTDs are presented in light gray (unsharpened map), while the D1 and D2 domains are shown in dark gray (sharpened map). The maps' sigma values are as follows (unsharpened: NTDs; sharpened: D1-D2 domains): C1 symmetry (σ=0.0040; σ=0.0170) and the final reconstruction (σ=0.0045; σ=0.0205). **(B)** FSC curves. FSC curve of the corrected map (blue) is shown following RELION post-processing. The resolution was determined by the FSC=0.143 criterion (pink). **(C)** Local resolution map. Local resolution of the reconstruction map from top, side and bottom views. The maps were calculated using RELION. Resolution scale bar is presented below the different views scaling from 3.2-6.0Å (purple to pink).

Fig. S16.

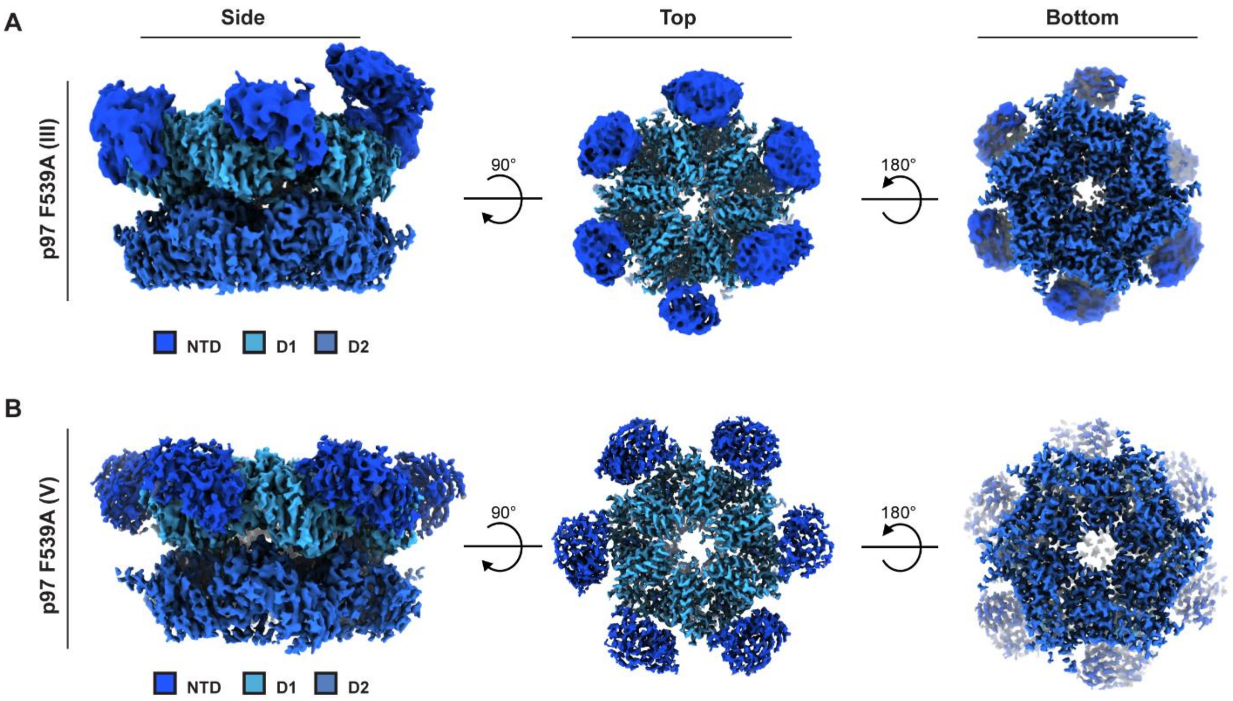

Figure S16: Single NTD in the "up" state (III) and all NTDs in the ADP state (IV) of p97 F539A maps characterization, related to Fig. 6. (A) Cryo-EM sharpened map of the single NTD in the "up" state (III) of p97 F539A. The three main domains are marked: (NTD) royal blue, (D1) sky blue and (D2) cornflower blue. The NTDs (unsharpened map) are shown at σ=0.0043, while D1 and D2 domains (sharpened map) are presented at σ=0.0150. **(B)** Cryo-EM sharpened map of all NTDs in the "down" state (IV) of p97 F539A. The three main domains are marked: (NTD) royal blue, (D1) sky blue and (D2) cornflower blue. The NTDs (sharpened map) are shown at σ=0.0045, while D1 and D2 domains (sharpened map) are presented at σ=0.0205.

Fig. S17.

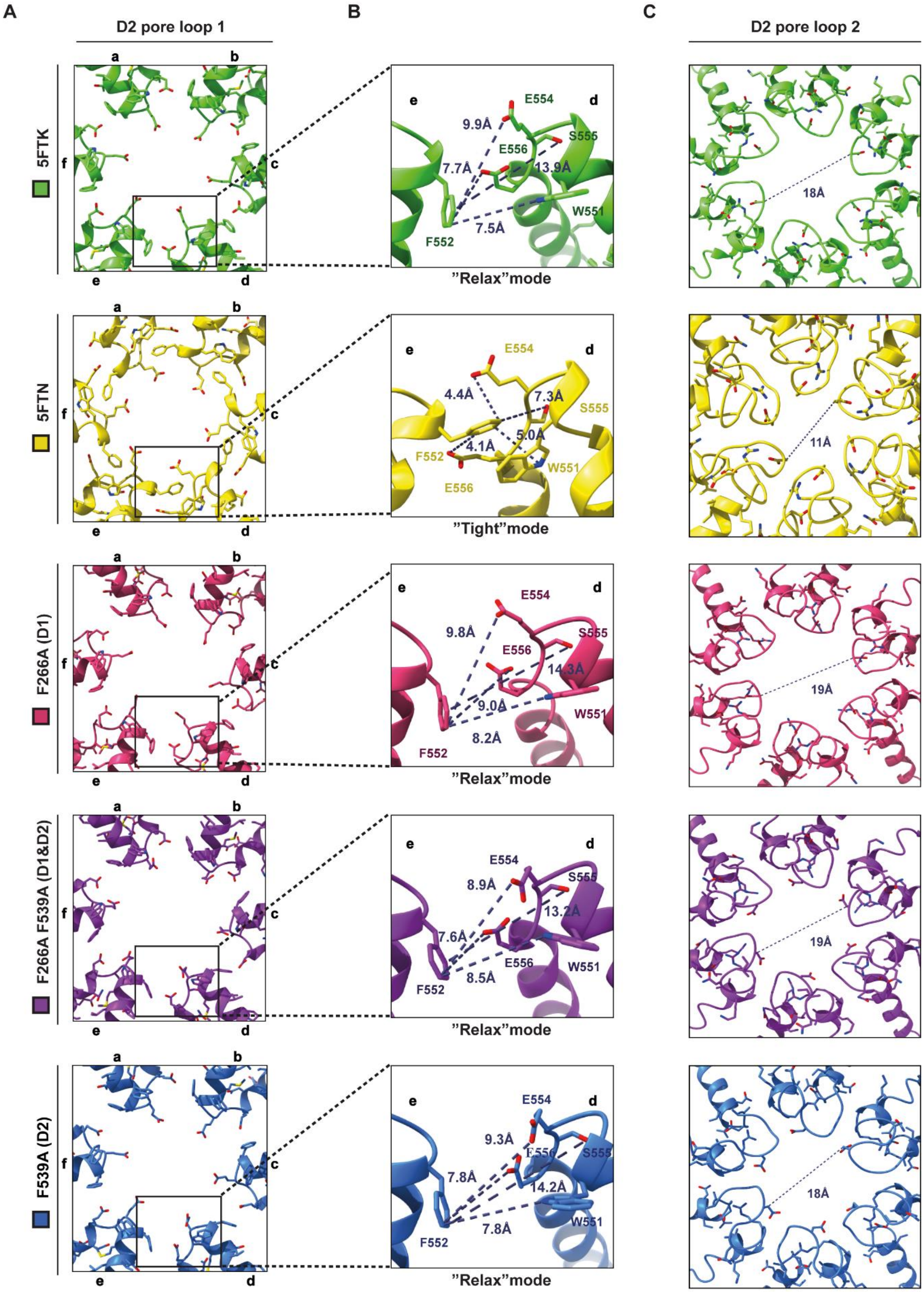
A
B
C
D2 pore loop 1
D2 pore loop 2
5FTK
5FTN
F266A (D1)
F266A F539A (D1&D2)
F539A (D2)
a
b
c
d
e
f
E554
S555
E556
W551
F552
9.9Å
7.7Å
13.9Å
7.5Å
"Relax"mode
18Å
4.4Å
7.3Å
5.0Å
4.1Å
"Tight"mode
11Å
9.8Å
14.3Å
9.0Å
8.2Å
19Å
8.9Å
13.2Å
7.6Å
8.5Å
9.3Å
7.8Å
14.2Å

Figure S17: ADP state in D2 ring induces the "Relax" mode, which is necessary for substrate entry and commitment, related to Fig. 7. (A) D2 pore loop 1 of p97 phenylalanine mutants is similar to that of wild type in the ADP state. Comparison between the pore loop 1 in D2 ring (P545-N558) of the wild type p97 different nucleotide states: ADP (green) and ATPγS (yellow), modified from the PDBs 5FTK and 5FTN respectively, and F266A (D1, pink), F266A F539A (D1&D2, purple) and F539A (D2, blue). **(B)** F552 mobility is nucleotide dependent. Magnification of D2 pore loop 1; focusing on F552 whose mobility is regulated by the nucleotide state, as well as its interaction with the following residues: E554, S555, E556 and W551. The ATPγS state (5FTN) corresponds to a "Tight" conformation for F552, whereas its motion is restricted by the indicated side chains. In contrast, the ADP state (5FTK) displays the "Relax" conformation allowing the mobility of F552 side chain. p97 variants display similar "Relax" mode. **(C)** D2 pore loop 2 is dilated in the ADP state. Comparison between the pore loop 2 in D2 ring (D580-I604) of the wild type p97 different nucleotide states: wild type ADP (5FTK), wild type ATPγS (5FTN), p97 F266A (D1) and p97 F266A F539A (D1&D2). The pore loop in ATPγS state is narrower (~11Å) compared with ADP states presented by wild type p97 (5FTK), p97 F266A (D1), p97 F266A F539A (D1&D2) and p97 F539A (D2) (~19Å).

Table S1.

Cryo-EM data collection, refinement, and validation statistics for the p97 phenylalanine mutants' datasets, related to Fig. 3-6.

	p97 F266A (D1) (EMD-18790) (PDB: 8R0E)	p97 F539A (D2) (EMD-19476) (PDB: 8RSC)	p97 F266A F539A (D1&D2) (EMD-19473) (PDB: 8RS9)	p97 F539A-ADP state (V) (EMD-19475) (PDB: 8RSB)	* p97 F539A-(III) (EMD-17827) (PDB: 8PQX)	p97 F539A (D2) +ATP (EMD-17837)	p97 F539A-adaptor (EMD-18517)
Data collection and processing							
Magnification	96,000	96,000	96,000	96,000	96,000	96,000	96,000
Voltage (kV)	300	300	300	300	300	300	300
Electron exposure (e^{-}/Å^2)	~0.9	~0.9	~0.85	~0.85	~0.9	~0.9	~1.0
Defocus range (μm)	-0.5-(-1.5)	-0.5-(-1.5)	-0.8-(-2.0)	-0.5-(-1.5)	-0.8-(-2.0)	-0.8-(-2.0)	-0.8-(-1.8)
Pixel size (Å)	0.86	0.86	0.86	0.86	0.793	0.793	0.793
Symmetry imposed	C6	C1	C6	C1	C1	C1	C1
# Micrographs used	9,238	2,800	11,732	10,001	21,856	7,256	10,776
Initial particle images (no.)	2,910,812	1,156,843	3,005,242	4,320,975	5,686,568	2,687,425	2,448,068
Final particle images (no.)	216,973	162,750	79,612	186,932	163,728	97,613	399,769
Map resolution (Å)	2.7	3.6	3.4	3.4	3.3	3.2	3.0
FSC threshold	0.143	0.143	0.143	0.143	0.143	0.143	0.143
Model refinement							
Initial model used (PDB code)	5FTN	5FTN	5FTN	EMD-20730	EMD-20730	5FTN	EMD-20730
Model resolution (Å)	3.3	3.3	3.3	4.26	4.26	3.3	4.26
FSC threshold	0.143	0.143	0.143	0.143	0.143	0.143	0.143
Map sharpening B factor (Å^2)	-102	-151	-111	-105	-102	-100	-104
Model composition							

Nonhydrogen atoms	32,056	31,874	31,359	31,884	26728	-	-
Protein residues	4524	4457	4458	4452	3402	-	-
Ligands	0	10	0	12	12	-	-
B factors (Å^2)							
Protein	46.27	47.17	117.27	36.10	133.67	-	-
Ligand	-	33.20	-	18.38	66.29	-	-
R.M.S.D							
Bond lengths (Å)	0.012	0.003	0.003	0.003	0.003	-	-
Bond angles (°)	1.207	0.670	0.685	0.738	0.577	-	-
Validation							
MolProbity score	1.86	1.78	1.99	1.23	1.95	-	-
Clashscore	5.05	5.97	8.36	5.68	11.88	-	-
Poor rotamers (%)	0.07	0.11	0.15	0.21	0.00	-	-
Ramachandran plot							
Favored (%)	88.12	92.94	90.44	95.56	94.69	-	-
Allowed (%)	11.88	7.06	9.56	4.44	5.31	-	-
Disallowed (%)	0	0	0	0	0	-	-

***The model composition refinement data does not include the NTDs.

Table S2.

Summarized table of yeast strains used in this study, related to Fig. 1F.

Strain	Vector	Genotype	Reference
BY4742α	-	*MAT α; his3Δ1; leu2Δ0; lys2Δ0; ura3Δ0*	[12]
	- (Empty vector)	*MAT α; his3Δ1; leu2Δ0; lys2Δ0; ura3Δ0; pRS41H (hph)*	This study
	CDC48 WT	*MAT α; his3Δ1; leu2Δ0; lys2Δ0; ura3Δ0; pRS41H-CDC48 (hph)*	This study
cdc48Δ	*CDC48 WT*	*MAT α; his3Δ1; leu2Δ0; lys2Δ0; ura3Δ0; cdc48::KanMX; pRS41H-CDC48 WT (hph);*	This study
	CDC48 F276A	*MAT α; his3Δ1; leu2Δ0; lys2Δ0; ura3Δ0; cdc48::KanMX; pRS41H-CDC48 F276A (hph);*	This study
	CDC48 F549A	*MAT α; his3Δ1; leu2Δ0; lys2Δ0; ura3Δ0; cdc48::KanMX; pRS41H-CDC48 F549A (hph);*	This study
	CDC48 F276A F549A	*MAT α; his3Δ1; leu2Δ0; lys2Δ0; ura3Δ0; cdc48::KanMX; pRS41H-CDC48 F276A F549A (hph);*	This study

Table S3.

Summarized table of the primers used for colony PCR, related to Fig. 1F.

Primer sequence (5'-3')	Position	Fragment size
(F): GTTGCATGACATCCCGGAC	360bp upstream to *CDC48* gene	~1300bp
(R): GGTCTGCGATTCCGACTC	*KANMX* gene	

(F): forward, (R): reverse.

Table S4.

Summarized table of the R^2 values from Hill equation fit, related to Fig. 2A-B.

p97 WT	p97 F266A (D1)	p97 F539A (D2)	p97 F266A F539A (D1&D2)	p97 F539A (D2)
+ATPγS	-	-	-	+ATPγS
R^2= 0.9841	R^2= 0.9854	R^2= 0.9959	R^2= 0.9928	R^2= 0.9965

Table S5.

Summarized table of the R^2 values from logistic curve fit, related to Fig. 1F.

Temperature	BY4742α (PS)	p97 WT	p97 F266A (D1)	p97 F539A (D2)	p97 F266A F539A (D1&D2)
25	R^2= 0.9985	R^2= 0.9939	R^2= 0.9918	R^2= 0.9783	R^2= 0.9946
30	R^2= 0.9900	R^2= 0.9839	R^2= 0.9862	R^2= 0.9523	R^2= 0.9834
37	R^2= 0.9974	R^2= 0.9980	R^2= 0.9955	R^2= 0.9670	R^2= 0.9613

References

1. Banerjee, S., Bartesaghi, A., Merk, A., Rao, P., Bulfer, S.L., Yan, Y., Green, N., Mroczkowski, B., Neitz, R.J., Wipf, P., et al. (2016). 2.3 A resolution cryo-EM structure of human p97 and mechanism of allosteric inhibition. Science *351*, 871-875. 10.1126/science.aad7974.
2. Bodnar, N.O., Kim, K.H., Ji, Z., Wales, T.E., Svetlov, V., Nudler, E., Engen, J.R., Walz, T., and Rapoport, T.A. (2018). Structure of the Cdc48 ATPase with its ubiquitin-binding cofactor Ufd1-Npl4. Nat Struct Mol Biol *25*, 616-622. 10.1038/s41594-018-0085-x.
3. Pan, M., Zheng, Q., Yu, Y., Ai, H., Xie, Y., Zeng, X., Wang, C., Liu, L., and Zhao, M. (2021). Seesaw conformations of Npl4 in the human p97 complex and the inhibitory mechanism of a disulfiram derivative. Nat Commun *12*, 121. 10.1038/s41467-020-20359-x.
4. Blythe, E.E., Gates, S.N., Deshaies, R.J., and Martin, A. (2019). Multisystem Proteinopathy Mutations in VCP/p97 Increase NPLOC4.UFD1L Binding and Substrate Processing. Structure *27*, 1820-1829 e1824. 10.1016/j.str.2019.09.011.
5. Huang, R., Ripstein, Z.A., Rubinstein, J.L., and Kay, L.E. (2020). Probing Cooperativity of N-Terminal Domain Orientations in the p97 Molecular Machine: Synergy Between NMR Spectroscopy and Cryo-EM. Angew Chem Int Ed Engl *59*, 22423-22426. 10.1002/anie.202009767.
6. Pan, M., Yu, Y., Ai, H., Zheng, Q., Xie, Y., Liu, L., and Zhao, M. (2021). Mechanistic insight into substrate processing and allosteric inhibition of human p97. Nat Struct Mol Biol *28*, 614-625. 10.1038/s41594-021-00617-2.
7. van den Boom, J., Marini, G., Meyer, H., and Saibil, H.R. (2023). Structural basis of ubiquitin-independent PP1 complex disassembly by p97. EMBO J *42*, e113110. 10.15252/embj.2022113110.
8. Twomey, E.C. (2021). Aboard the ISS: intersubunit signaling revealed in the p97 ATPase. Nat Struct Mol Biol *28*, 538-539. 10.1038/s41594-021-00622-5.
9. Twomey, E.C., Ji, Z., Wales, T.E., Bodnar, N.O., Ficarro, S.B., Marto, J.A., Engen, J.R., and Rapoport, T.A. (2019). Substrate processing by the Cdc48 ATPase complex is initiated by ubiquitin unfolding. Science *365*. 10.1126/science.aax1033.
10. Cooney, I., Han, H., Stewart, M.G., Carson, R.H., Hansen, D.T., Iwasa, J.H., Price, J.C., Hill, C.P., and Shen, P.S. (2019). Structure of the Cdc48 segregase in the act of unfolding an authentic substrate. Science *365*, 502-505. 10.1126/science.aax0486.
11. Xu, Y., Han, H., Cooney, I., Guo, Y., Moran, N.G., Zuniga, N.R., Price, J.C., Hill, C.P., and Shen, P.S. (2022). Active conformation of the p97-p47 unfoldase complex. Nat Commun *13*, 2640. 10.1038/s41467-022-30318-3.
12. Brachmann, C.B., Davies, A., Cost, G.J., Caputo, E., Li, J., Hieter, P., and Boeke, J.D. (1998). Designer deletion strains derived from Saccharomyces cerevisiae S288C: a useful set of strains and plasmids for PCR-mediated gene disruption and other applications. Yeast *14*, 115-132. 10.1002/(SICI)1097-0061(19980130)14:2<115::AID-YEA204>3.0.CO;2-2.
